# An enzyme-centric approach for modelling non-linear biological complexity

**DOI:** 10.1186/1752-0509-2-70

**Published:** 2008-08-01

**Authors:** Chin-Rang Yang

**Affiliations:** 1 Harold C. Simmons Comprehensive Cancer Center, University of Texas Southwestern Medical Center at Dallas, Dallas, Texas, USA

## Abstract

**Background:**

The current challenge of Systems Biology is to integrate high throughput data sets for simulating the complexity of biological networks, exploit the evolution of nature-designed networks that maintain the robustness of a biological system, and thereby generate novel, experimentally testable hypotheses. In order to simulate non-linear biological complexities, we have previously developed an Enzyme-Centric mechanistic modeling approach and validated it using metabolic network in *E. coli*. The idea is to use prior knowledge of catalytic and regulatory mechanisms of each enzyme within the metabolic network to build a dynamic model for investigating the network level regulation and thus understand the nature design principle behind the network.

**Results:**

In this paper, we further demonstrate the application of complex enzyme catalytic and regulatory modules to simulate nonlinear network regulatory patterns vs. simple linear conversion model. We learned and validated that it is essential to incorporate prior knowledge from the literature to simulate non-linear biological complexities. The network expandability is demonstrated and validated with the complex amino acid biosynthetic network with multi-regulations. Also, we demonstrated the compatibility of mechanistic models within close species. Furthermore, the eukaryotic protein factory model for insuring steady mRNA production is simulated and the coupling of RNA transcription and splicing is validated by both mathematical simulation and experimental analysis.

**Conclusion:**

We demonstrated the importance of modeling complex enzyme catalytic and regulatory mechanisms to further understand nonlinear network regulatory patterns. The simulations presented in this paper reveal how a living system maintains homeostasis and its robustness to continue functioning while facing environmental stresses or genetic mutations.

## Background

Remarkable advances in the high throughput technologies enable researchers to broaden their research focuses from a single gene/protein to global gene/protein expression profiles. The daunting challenge is how to turn these overwhelmingly data into real information and gain meaningful insights of how the information is processed in a living system. The goal of Systems Biology is to integrate these high throughput data sets through mathematical models that can computationally simulate the complexity of a biological network, explore the design principles during the evolution of a biological circuit to insure its robustness and thereby generate novel, experimentally testable hypotheses. It is the mission in the post-genomic era to understand how all the parts of cells – genes, proteins, and many other molecules – work in concert to create complex living organisms and analyze how entire biological systems function, both in health and in sickness.

The main challenge for systems biologists is to develop a mathematical modeling technique suitable for modeling the complexity of biological systems. The "Enzyme-Centric" mechanistic modeling approach has been developed in order to integrate expert knowledge in the mechanistic understanding of enzyme catalytic and regulatory mechanisms [1,2]. Various enzyme models can be assembled into a pathway such that the combination of various pathways is automatically extended into a larger biological network. The modularity of the model and the feature of automatic model generation enables flexible updates. This approach incorporates non-linear biological properties which are essential for simulating the network level regulation and predicting responses to perturbations.

In contrast, commonly seen modeling approaches either ignore the dynamic properties of enzymes by assuming they are constant or, without considering regulation, simply assign probability linkage among components through training sets. The term "non-linear" here refers to the biological nonlinearity of enzymes that are variables in the equations (not mathematical non-linearity). The amino acid biosynthetic network and the interaction of transcription and RNA splicing are presented here to show the advantages of using the non-linear Enzyme-Centric approach vs. a linear conversion model. These examples reveal the value of Enzyme-Centric modeling to help understand how a living system maintains homeostasis and continues to function (robustness) while facing environmental stresses or genetic mutations.

## Results

### The Modularity of Non-linear Enzyme-Centric Modeling of the Amino Acid Biosynthetic Network

**Figure 1:**
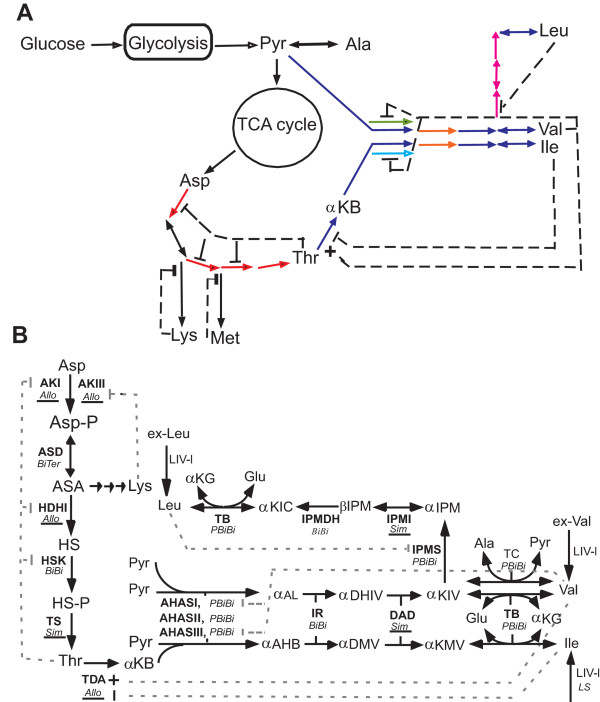
**Simulation of Metabolic Flux from Aspartate to the Branched Chain Amino Acid (BCAA) Biosynthetic Pathways in *E. coli *K12**. A. The metabolic network from glucose, through TCA cycle, to amino acids. The mathematical model of colored pathways was simulated here. Each color represents an operon that regulates the same color enzyme levels in the network. B. The abbreviations of enzymes: TDA, threonine deaminase; AHAS, acetohydroxy acid synthase; IR, acetohydroxy acid isomeroreductase; DAD, dihydroxy acid dehydrase; TB, transaminase B; TC, transaminase C; IPMS, α-isopropylmalate synthase; IPMI, α-isopropylmalate isomerase; IPMDH, β-isopropylmalate dehydrogenase; LIV-I, leucine, isoleucine, and valine transporter I; LS, leucine specific transporter, AKI, aspartate kinase I; AKIII, aspartate kinase III; HDHI, homoserine dehydrogenase I; ASD, semialdehyde dehydrogenase; HSK, homoserine kinase; TS, threonine synthase. The abbreviations of metabolites: Thr, threonine; Ile, isoleucine; Val, valine; Leu, leucine; Glu, glutamate; Ala, alanine; Pyr, pyruvate; αKB, α-ketobutyrate; αAL, α-acetolactate; αAHB, α-aceto-hydroxybutyrate; αDHIV, α,β-dihydroxy-isovalerate; αDMV, α, β-dihydroxy-β-methylvalerate; αKIV, α-ketoisovalerate; αKMV, α-keto-β-methylvalerate; αKG, α-ketoglutarate; αIPM, α-isopropylmalate; βIPM, β-isopropylmalate; αKIC, α-ketoisocaproate; ex-Ile, extracellular isoleucine; ex-Val, extracellular valine; ex-Leu, extracellular leucine, Asp, aspartate; AspP, Aspartyl phosphate; ASA, aspartate semialdehyde; Hse, homoserine; HseP, homoserine phosphate. kMech models used for each enzyme are italicized. Allo: allosteric, Sim: simple, PBiBi: Ping Pong Bi Bi enzyme mechanisms. Enzyme reactions are indicated by arrows. Feedback inhibition patterns are indicated by dashed lines. Activation is indicated by a plus sign, and inhibitions are indicated by vertical bars.

The validated Enzyme-Centric models for the branched chain amino acids (BCAA: isoleucine, valine and leucine) [1] and its upstream threonine biosynthetic pathways [3] in *Escherichia coli *(*E. coli*) K12 strain were integrated together to demonstrate the modularity and expandability of this approach (Fig 1A&B) [Additional file 1: Mathematica™ codes and parameter values]. This network includes seventeen enzymes (four of which are allosteric enzymes), eight feedback regulation loops, three sets of isozymes and several multi-functional enzymes of multi-functional pathways. This integrated model represents the first example of modeling "network level regulatory patterns" of multifunctional pathways. The non-linear, dynamic responses of intermediate metabolites due to the feedback regulations were observed. There are only three simple enzyme (i.e. one substrate and one product) models (Fig 1B, underlined Sim) in the network which emphasize the importance of modeling the complex enzyme model. Although there are only four allosteric enzymes (Fig 1B, underlined Allo), they are located at the upstream of each pathway. It is important to model them correctly [3] in order to simulate the regulation of the metabolic flux entering the individual pathway.

**Figure 2:**
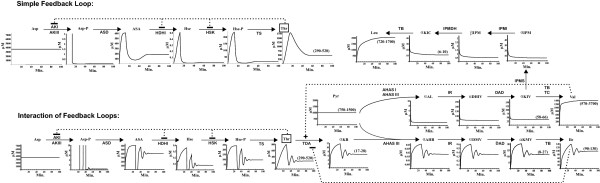
**Interaction of Feedback Loops.** Simple Feedback Loops: Simulation of threonine (Thr) biosynthetic pathway starting from aspartate with three feedback inhibition loops. Interaction of Feedback Loops: Threonine biosynthetic pathway connected with the downstream BCAA pathways. The graphical insets show the approach (minutes) to steady state (μM) synthesis and utilization of the substrates, intermediates, and end-products of the pathways. Where available, the ranges of reported values for pathway intermediate and end-product levels in cells growing in a glucose minimal salts medium are shown in parentheses (μM) in the inset graphs. Dashed lines indicate feedback regulation. Plus sign is positive feedback and minus sign is negative feedback.

In Fig 2 with Simple Feedback Loops, a sharp rise in the concentrations of Asp-P, ASA, Hse and Hse-P at the beginning was followed by a sharp rise in the concentration of threonine (Thr). These initial increases in the concentrations of metabolites are feedback-inhibited by accumulation of threonine on AKI, HDHI and HSK. After connecting the downstream pathway, threonine levels are also controlled by its downstream enzyme (TDA) (Fig 2, Interaction of Feedback Loops). TDA enzyme activity is feedback inhibited by isoleucine (Ile) and feedback activated by valine (Val). The ripple effect (i.e. damping oscillation) is due to the balance between different regulatory mechanisms. At the end, all metabolites reach homeostasis (i.e. steady states) dynamically. We demonstrate here that, using the Enzyme-Centric approach, each individual pathway model can be built and validated independently and then added into a larger network model.

#### Non-linear Network Level Regulation vs. Linear Conversion Model: Prediction of Isoleucine Production over Pyruvate Perturbation

**Figure 3:**
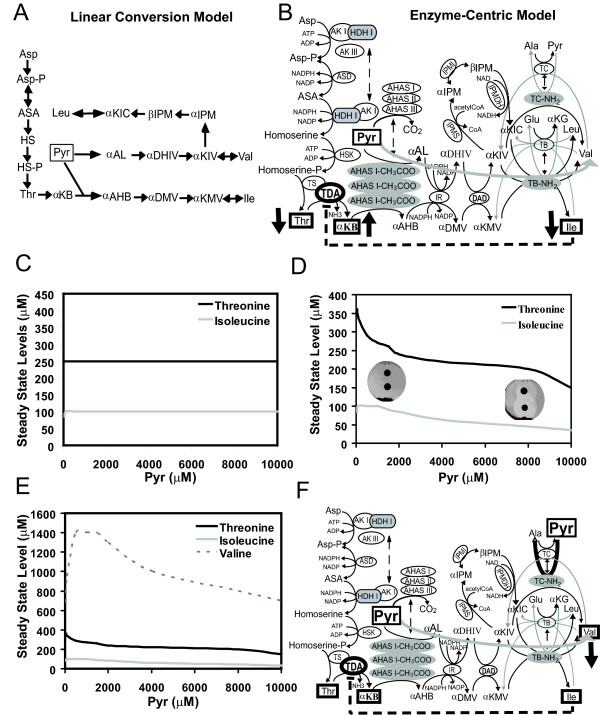
**Network Level Regulation.** A. The linear conversion model of the metabolic network. B. The corresponding non-linear, enzymecentric model. The grey arrow starting from Pyr depicts metabolic flow shifting to valine while pyruvate (Pyr) increasing. Black vertical arrows depict up- or down- regulated metabolites. C and D are the spectra of threonine (upstream of Pyr) and isoleucine (downstream of Pyr) responses over the Pyr perturbation using Model A and Model B, respectively. X-axis: Pyr from 50 to 10,000 uM. Y-axis: the simulated steady state levels of threoine and isoleucine at given Pyr concentrations. The dish images are growth assays given low (left) and high (right) Pyr. E. The spectrum of valine production using the Model B. F. The black arrow on the top right-hand corner depicts while Pyr passing the threshold, turning on the reverse TC enzyme reaction to switch the direction of metabolic flux.

It is important to demonstrate the difference between the non-linear, enzyme-centric model and linear conversion model. The easiest way is to do this is by introducing perturbation into both models. In Fig 3A and 3B, we introduce pyruvate (Pyr) perturbation. Changes in the levels of threonine (upstream of Pyr) and isoleucine (downstream of Pyr) are simulated. When perturbing Pyr in the linear model (Fig 3C), isoleucine reaches the max flux and remains constant while threonine stays in constant since it is upstream of Pyr and assumed independent from Pyr in the linear model. In contrast, with the enzyme-centric model (Fig 3D), products (isoleucine and threonine) are decreased as substrate (Pyr) increases.

To illustrate what happens in the non-linear model, in Fig 3B, AHAS, IR, DAD and TB are multi-functional enzymes shared by valine and isoleucine biosynthetic pathways. Excess Pyr disturbs the balance of enzyme partition and shifts most of enzymes to the valine biosynthesis. Decreased enzymes for the isoleucine pathway produce less isoleucine and the feedback inhibition of threonine deaminase (TDA) is relieved. TDA is more active to consume and decrease threonine level. To validate the response of the feedback regulation, the product of TDA, αKB, is a toxic metabolite that blocks glucose transport and cell growth. When TDA is activated, αKB accumulates, and AHAS enzymes become a rate limit step due to the shift of enzyme partition between two pathways. Using a simple growth assay, we validated that excess Pyr inhibits *E. coli *K12 growth (the right image of Petri dish in Fig 3D). This phenomenon cannot be explained by the simple linear conversion model.

It is essential to realize that under the network regulation, increasing substrate alone can not guarantee increased product amount. This is especially important for metabolic engineering. What we demonstrated here is the effect of multi-functional enzyme partition under increasing substrate condition on preventing over-production and maintaining homeostasis of an amino acid.

#### Non-linear Network Level Regulation vs. Linear Conversion Model: Prediction of Valine Production over Pyruvate Perturbation

While excess Pyr shifts enzymes to the valine biosynthesis, an interesting question is whether this affects valine production. Given more substrates and enzymes, the obvious answer is valine will increase, which is true if the reaction is in a test tube with purified enzymes. However, the simulated valine response pattern (dashed line in Fig 3E) tells a different story. Initially, valine increases in concert with Pyr level until saturation occurs. While Pyr keeps increasing and passes a certain threshold, an isozyme (transaminase C or TC) reaction is turned on (Fig 3F). TC converts valine reversibly back to its intermediate, αKIV. Once turned on, the TC reaction becomes a dominant reaction over the major transamination of transaminase B (TB), so that valine decreases as Pyr continues to increase.

This biological circuit is designed to automatically switch the direction of the metabolic flux to prevent over-producing and thus maintain homeostasis of valine. While the substrate levels increase, the outcome could be increasing, unchanged or decreasing, depending upon the given condition. Although the difference may not be obvious within the physiological levels of Pyr in both models (1000 uM in Fig 3C&D), under the extreme perturbation (e.g. drug treatments or gene mutations), the enzyme-centric model clearly shows a distinct response pattern.

#### Flexibility and Compatibility of Mechanistic Model within Close Species: Prediction of Drug Sensitivity in Salmonella LT2

The ability to reuse models within close species or cell lines is another advantage of the mechanistic modeling over statistic modeling. The model can be reused by changing a few parameters that are known to be different among species and validated without rebuilding the model. In contrast, statistical models require an entirely new training data set to train a new model.

We demonstrate here that *Escherichia coli *(*E. coli*) K12 and *Salmonella typhimurium *(*S. typh*.) LT2 are two closely related model organisms. However, several enzyme kinetic properties are known to be different: (i) *E. coli *K12 has non-functional, mutated AHAS isozyme II; *S. typh*. LT2 has normal AHAS II, but does not express AHAS III; (ii) LT2 has much higher TDA enzyme activity (*kcat *is over 10 fold increased) than K12; and, (iii) although the allosteric TDA is a tetramer in LT2 the same as in K12, it has only two functional catalytic sites. The allosteric parameters (*c *and *L*) were refitted to the kinetic data from LT2 as described in [3] [curve fitting shown in Additional file 2A]. The following are values of parameters in the models to reflect this knowledge: LT2: [AHAS II] = 8 uM, [AHAS III] = 0 uM, c = 0, L = 0.27 vs. K12: [AHAS II] = 0 uM, [AHAS III] = 2 uM, c = 0.013, L = 1.05.

**Figure 4:**
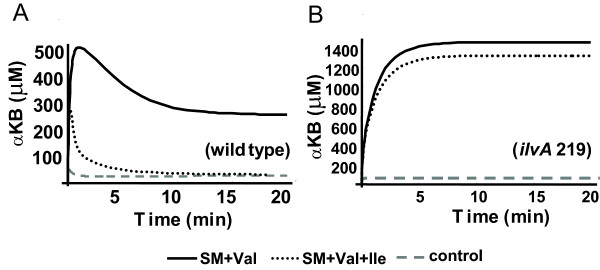
**Simulation of the Branched Chain Amino Acid (BCAA) Biosynthetic Pathways in *Salmonella *LT2.** A. Simulation of treatments in wild type (*ilvA*+) LT2; B. Simulation of treatments in the TDA_Ile feedback resistant mutant (*ilvA *219); αKB is an inhibitor for glucose transporter. Low αKB (Y-axis) indicates growth; high αKB indicates growth suppression. Solid line: Sulfometuron Methyl (SM) + valine (Val); Dotted line: SM + Val + Ile (isoleucine); Dashed line: control with no treatment.

The LT2 model was validated with the known drug toxicity effect on Sulfometuron Methyl (SM), which is an herbicide that specifically blocks AHAS II activity. The enzyme concentration of AHAS II was turned down to simulate the SM effect. The Ile feedback resistant mutant of TDA was simulated by increasing *Ki *of TDA to Ile to a large number. Again, using αKB as a growth indicator, when treated with SM + Val, SM inhibits AHAS II. Val inhibits AHAS I (downstream of αKB), but activates TDA (upstream of αKB). AHAS III is null in LT2 (Fig 1B). Threonine is rapidly converted to αKB which blocks glucose transport. Cell growth was suppressed in both strains [experimental results shown in Additional file 2B]. TDA is an allosteric enzyme that is activated by Val and inhibited by Ile. Ile can rescue the wild type strain (*ilvA*+) by competing with Val to inhibit TDA activity and eliminate αKB accumulation (simulation shown as dotted line in Fig 4A), but not in the TDA_Ile feedback mutant (*ilvA*219) (simulation shown as dotted line in Fig 4B). The results demonstrate that, with mechanistic modeling, we are able to switch the metabolic network model of *E. coli *to *S. typh *by changing a few parameter values. The simulations of drug sensitivity agree with known experimental results.

### Enzyme-Centric Modeling of the Coupling Effect of Spliceosome and RNA Polymerase

**Figure 5:**
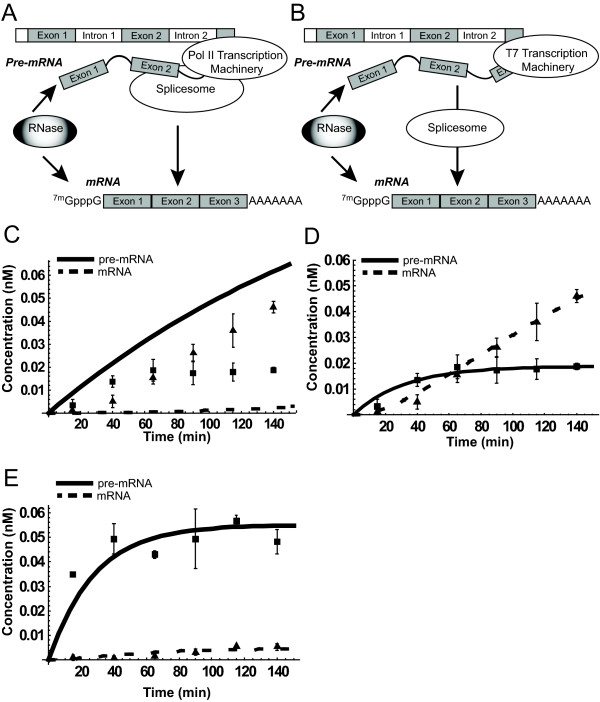
**The Interactome Model of RNA Transcription and Spliceosome.** A. The protein factory model of RNA Polymerase II and spliceosome. B. The T7 polymerase model without interacting with spliceosome. C. to E. Simulations of the *in vitro *RNA splicing assay. Black squares: measured premRNA; Solid lines: simulated pre-mRNA curves; Black triangles: measured mature mRNA; Dotted lines: simulated mRNA curves. Black bars: standard deviations of data. The interactome of Pol II and spliceosome (NEs) increases the affinity of NEs to the pre-mRNA (smaller *Km*) as demonstrated by perturbing *Km *(NEs to pre-mRNA). The value is 50 nM in C and 1 nM in D while *Km *(RNase to pre-mRNA) is 50 nM in both cases. D. The pre-mRNA protected by PolII interactome is in steady state to maintain mature mRNA production. E. The viral T7 polymerase simulation. Mature mRNA is rapid degraded without protection. *Km *(NEs to pre-mRNA) = *Km *(RNase to pre-mRNA) = 50 nM.

In eukaryotic cells, proper RNA processing is essential for producing proteins with normal functionality. The protein factory hypothesis suggests that the protein-protein interaction of RNA polymerase II (Pol II) and spliceosome protects the functional splice sites within nascent pre-mRNA from the RNase degradation and results in the efficient mature mRNA production (Fig 5A&D). In contrast, other RNA polymerases (e.g. viral T7) do not provide similar protection resulting in excess mature mRNA degradation (Fig 5B&E) [4]. The Enzyme-Centric interactome model of spliceosome and RNA polymerase was constructed, and after systematic perturbing, the key controlling factor that was identified is the affinity (*Km*) of spliceosome to pre-mRNA related to the affinity of RNase to pre-mRNA. The former needs to be much less (~1/50) than the latter (Fig 5C vs. 5D). Computer simulations support the protein factory hypothesis that the gene expression machinery in eukaryotic cells is closely interacted with each other to form an assembly line. We demonstrate that this circuit design guarantees an extended half-life for the proper processing of nascent pre-mRNAs and ensures that the quality and steady production of mature mRNA production (Fig 5D) vs. viral polymerase dose not warrant this functionality (Fig 5E). Furthermore, the deterministic Pol II model can be adapted to the stochastic simulation by adding normally distributed noise terms (Langevin equation) to the rate constants. The probabilistic perturbations of reaction rates for transcription and splicing also demonstrate that these two rate constants are the key factors to simulate the observed experimental variations [Additional file 3B].

## Discussion

In this paper, we demonstrate the application of complex enzyme catalytic and regulatory modules to simulate nonlinear network regulatory patterns. In order to simulate the non-linear biological complexities, it is essential to incorporate prior knowledge of enzyme mechanism from the literature. Simulation/perturbation of the integrated mathematical model from each individual enzyme models will help us to understand the network level regulation and the purpose of the circuit design. The model expandability is demonstrated; each individual amino acid metabolic pathway is simulated/validated independently and then adds into a larger network model to form a complex amino acid biosynthetic network with layers of multi-regulations. These regulatory mechanisms, including multi-functional enzyme partition and reversible isozyme reaction, contribute to prevent over-production and maintain homeostasis of amino acids under environmental changes. The purposes of these metabolic circuit designs are never realized by investigators when the focus is on studying the mechanism of a single enzyme. Simulations using the integrated, non-linear Enzyme-Centric model uncover the purposes of these designs. To simulate and understand these properties are especially important for metabolic engineers to design mutant strains that remove these regulatory mechanisms that protect microorganism from over-production to increase yield of amino acid production.

We also demonstrated the flexibility of mechanistic models within close species by changing a few parameter values. The model can easily be re-used from one species to the other. Furthermore, the eukaryotic protein factory model for ensuring steady mRNA production is simulated and the coupling of RNA transcription and splicing is validated by both mathematical simulation and experimental analysis. This circuit design guarantees an extended half-life for the proper processing of nascent pre-mRNAs and ensures the quality and steady production of mature mRNA production, which does not occur in viral RNA polymerase.

### Comparison of Non-linear Enzyme-Centric Approach to Other Models: The Key for Modeling the Biological Non-Linearity is "Enzyme is a Variable"

**Figure 6:**
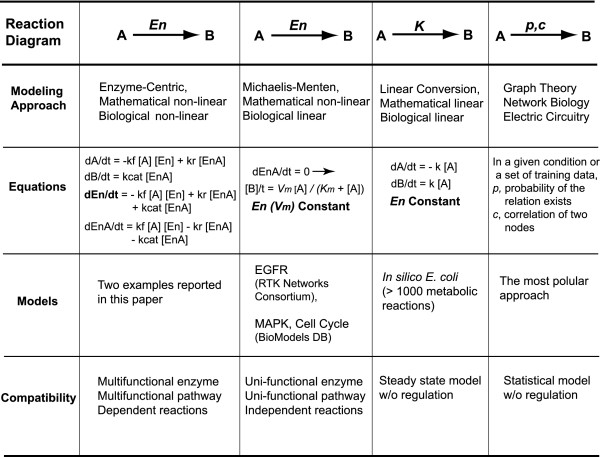
Comparison of Current Modeling Approaches Using the Simple Enzyme Model.

Traditional enzyme modeling approaches use the *Michaelis-Menten *kinetic equation for one substrate/one product reactions while the *King-Altman *method is used to derive equations for more complex multiple reactant reactions. These types of equations are called steady-state velocity equations since the derivatives of the concentration over time for each reactant in the model are set to zero to simplify a set of nonlinear differential equations to linear algebra equations. This type of approach may be suitable for single enzyme or unifunctional pathway modeling (Fig 6). *Michaelis-Menten *equation is mathematically non-linear but biologically linear, since the *Vm *(*Vmax*, the maximum flux of the reaction) in the equation is a constant and *Vm *= *kcat *× [*En*]total. *kcat *is a constant, therefore [*En*]total is a constant. This assumption is valid if the model only concerns the reactions inside a test tube, but not for multifunctional pathways and network-level regulation. In the field of metabolic engineering, the most widely used modeling framework is metabolic control analysis (MCA). The major limitation of the traditional MCA is its assumption that a system is always in a steady state, i.e. the assumption of linearity and independency (Fig 6). Therefore, the traditional MCA approach is not suitable for modeling the transient phenomena from perturbations of metabolic parameters. Several improvements have tried to remove these limitations, including the Power-law approximation (S-system) [5], the (Log)Linear refinement of the MCA model [6] and lin-log kinetics [7] which have been developed to consider enzyme concentrations, feedback regulators and reversible enzyme reactions in the models.

The network level regulation involving multiple regulatory mechanisms is beyond the concept of a simple feedback loop, linear conversion [8] or statistic model. Regulatory proteins which are critical for the robustness and integrity of the system can be identified using numerical perturbations as shown above. In general, such regulatory proteins are located at the upstream branch points of a network. Usually, multiple regulators or isoforms control the direction or partition of metabolic flux (e.g. TDA and AHAS). Nevertheless, downstream regulators are also observed (e.g. TC) under certain conditions. Computers can generate spectra of dynamic responses over biological perturbations to facilitate the identification of regulatory proteins and thereby explicate the design principles of nature-occurring biological circuits.

Unfortunately, biology is more complicated than those models can realistically reflect without the introduction of numerous extra dynamic variables. For example, in the metabolic network model below, to model the isozymes (AKI&III and AHASI&II&III) that are controlled by different modes of regulation, the MCA models must allow multiple fluxes for the conversion of the same substrate to the same product. To model the bi-functional enzyme (AKI-HDHI, the same protein carrying two enzyme activities), the fluxes of these two enzyme reactions must be "dependent" on each other. Also, several enzymes are involved in multi-pathways and their fluxes depend on the enzyme partition between these pathways in a given condition. These facts point out the fundamental drawback of the MCA approach in considering metabolic networks as a collection of "independent" chemical conversions. With enzymes remaining as variables, the Enzyme-Centric approach allows the temporal patterns of enzyme state/partition and network-specific regulatory patterns to be identified and is able to incorporate non-linear properties of a biological network, such as positive/negative feedback regulation, allosteric regulation [3], reversible enzyme reaction, post-translational modification (intermediate enzyme states), biological redundancy (isozymes/isoforms), multifunctional enzyme and its partition between the multifunctional pathways [1]. In contrast, commonly seen modeling approaches mainly focus on discovering correlations or probability linkages between molecular species using specific training sets. Regulation is not considered to simplify the complexity of their models (Fig 6). Nevertheless, simulations presented here demonstrate these non-linear regulatory properties are essential for modeling the network level regulation that maintains the robustness of a biological system.

## Conclusion

In conclusion, we recognize biological complexity and develop a novel modeling tool to integrate prior mechanistic knowledge into a mathematical model. With perturbing the parameters in the model systematically, regulatory factors critical to maintaining the functional integrity of the system were found. We demonstrated the importance of modeling complex enzyme catalytic and regulatory mechanisms to further understand the nonlinear network regulatory patterns and circuit design for preventing of over-production and thereby maintaining homeostasis. The simulations presented in this paper reveal how a living system maintains homeostasis and robustness to continue functioning while facing environmental stresses and also strengthen the idea of bringing knowledge and regulatory mechanisms into computer simulation [8] to make a model smart enough, and, as such, become an engine of discovery and prediction.

## Methods

### Enzyme-Centric Modeling Approach

The idea of "Enzyme-Centric" modeling is to understand common enzyme catalytic and regulatory mechanisms in biological processes, and then integrate individual enzyme models into a pathway so various pathways assemble into a larger biological network [1,2]. The focus is on mining the expert knowledge of individual enzymes studies by different laboratories from the literature to identify molecular interactions and regulatory patterns of each enzyme (e.g. feedback and allosteric regulation). Special attention is given to enzyme isoforms and multifunctional enzymes which are essential for the reactive flux distribution within the network [1]. The mathematical tools for the Enzyme-Centric modeling, including kMech (enzyme kinetics), GMWC (Generalized MWC model for multi-ligand allosteric regulation) and Cellerator, are freely available to noncommercial users http://www.cellerator.org. They can be executed in Windows, MacOS, or Linux within Mathematica™. Alternatively, Cellerator can generate ordinary differential equations (ODEs) in System Biology Markup Language (SBML).

### Network Extension: The Modularity of Enzyme-Centric Modeling

Yang et al. 2005 [1] and Najdi et al. 2006 [3] have used the Enzyme-Centric approach to model the regulated flow of metabolites through the multi-functional branched chain amino acids (BCAA, isoleucine, valine and leucine) biosynthetic pathways and their upstream threonine biosynthesis in the model organism, and *E. coli *K12, respectively (Fig 1A). Both models have been validated with several known genetic and biochemical perturbations. To demonstrate the modularity and expandability of kMech, we simply put these two models together in the simulator to form a network of four interacting metabolic pathways. Cellerator automatically regenerates 131 ODEs with 189 rate parameters for the new model consisting of 17 enzymes [Additional file 1: Mathematica™ codes and parameter values]. The enzyme mechanisms of these pathways include simple catalytic, Bi Bi, Ping Pong Bi Bi, and Bi Ter mechanisms that are regulated by either allosteric, competitive, noncompetitive inhibition or activation mechanisms (Fig 1B). The connecting points of these four pathways (Thr, Pyr and αKIV) and eight feedback loops are automatically found and connected by the computer. In the reported simulations, steady-state enzyme activity levels were optimized to properly channel the steady-state flow of metabolic intermediates through these pathways at levels that match their reported *in vivo *levels and the concentrations of enzyme cofactors, ATP and NAD(P)(H) were kept as constants [3,1].

### Rate Constant Approximation

One important issue of mechanistic modeling is how to obtain kinetic rate constants for simulation. It is a difficult task to measure the forward and reverse rate constants (*kf, kr*) experimentally. Alternatively, the rate constants of metabolic enzymes are approximated from easily measured kinetic constants *Km *(*Michaelis-Menten *constant) and *kcat *(catalytic constant or enzyme turnover number) using the Lambda (Λ) approximation which is previously developed and implemented in kMech [1,2]. In brief, the approximation introduces a new parameter Λ that represents the ratio of forward reaction flux of the enzyme-substrate complex formation to the catalytic flux of product production. In other words, when Λ is large, the enzyme-substrate complex approaches steady state very fast. This is the same as the *Michaelis-Menten *pseudo-steady state assumption [9]. The flexibility of data fitting using the Λ approximation over the *Michaelis-Menten *equation is also illustrated in Additional file 3A. The values of Λ are empirically adjusted to fit experimental data. In the case of metabolic network simulation, the values of Λ can be varied from 10 to 1,000,000 with no significant changes in the steady levels of intermediates and end-products. Therefore, the Λ is set to 100 for all enzymes in the model [Additional file 1: Mathematica™ codes and parameter values]. However, in the RNA splicing model, the binding of RNA polymerase to the DNA template is a slow process, and the Λ is set to 1 to fit the measured results [Additional file 4: Mathematica™ codes and parameter values]. However, not all biological pathways are as well studied as the metabolic network (i.e. *Km *and *kcat *are not always available). One solution for this challenge is to apply the quantitative time course data after certain treatments to constrain the model and approximate parameter spaces for kinetic constants as demonstrated in the RNA splicing model below.

The other issue of dealing with a large number of parameters is how to prevent over-fitting. The integrated metabolic network model has total of 189 rate parameters. To avoid the over-fitting problem, the key is to understand the parameters. For each enzyme, at least three parameters are needed: total enzyme concentration ([*En*]total), affinity to all of its substrates (*Km*), and reaction rate (*kcat*). If the enzyme is regulated by additional factors, more parameters are added (e.g. *Ki *for affinity to an inhibitor; *Ka *for an activator). The *Monod, Wyman, Changeux *model [3] is used for modeling an allosteric enzyme with two additional allosteric parameters: *L *(partition of active and inactive enzymes) and *c *(affinity of substrate to the inactive enzyme). In other words, we only introduced biological meaningful parameters into the model to prevent the problems of over-fitting.

### The Interactome of RNA Transcription and Spliceosome: The Mathematical Model of RNA Splicing

The mathematical model of RNA splicing was built using the Enzyme-Centric approach. Each enzyme mechanism is parsed by kMech into a set of fundamental association-dissociation reactions that are translated by Cellerator into ordinary differential equations (ODEs) that are numerically solved by Mathematica™. The pathway diagram of the interaction between transcription and RNA splicing is shown below:

where NE1 is RNA polymerase (either human Pol II or viral T7), NEs is spliceosome, and NEd is RNase. The model consists of three major reactions: Transcription of DNA, Splicing of pre-mRNA and Degradation of all mRNA. This reaction model can be represented by the following six kMech/Cellerator reactions:

$$\begin{array}{l} \text{mRNASplcing}=\text{Union}\underset{\text{NE}1}{[}\\ \text{Enz}\left[\left\{\text{DNA},\text{ NTP}\}⇄\{\text{premRNA},\text{ DNA}\},\text{BiBi }[\text{kf}1,\text{ kr}2,\text{ kcat}3\right]\right],\\ \{\{\text{premRNA}+\text{NEs}⇄\$\text{Complex}\$\text{NEs}\$\text{premRNA}\$,\text{ kf}4,\text{ kr}5\}\},\\ \{\{\$\text{Complex}\$\text{NEs}\$\text{premRNA}\$\to \text{NEs}+\text{mRNA},\text{ kcat}6\}\},\\ \{\{\text{premRNA}\overset{\text{NEd}}{⇄}\text{NMP},\text{ kf}7,\text{ kr}8,\text{ kcat}9\}\},\\ \{\{\$\text{Complex}\$\text{NEs}\$\text{premRNA}\$\overset{\text{NEd}}{⇄}\text{NMP},\text{ kf}10,\text{ kr}11,\text{ kcat}12\}\},\\ \{\{\text{mRNA}\overset{\text{NEd}}{⇄}\text{NMP},\text{ kf}13,\text{ kr}14,\text{ kcat}15\}\}\\ ] \end{array}$$

The 1st reaction represents "Transcription of DNA" modeled by the kMech generalized BiBi (two-substrate, two-product) reaction. NTP represented four nucleotides required for transcription. The 2nd reaction represents spliceosome binds to pre-mRNA modeled by the Cellerator simple catalytic model. The 3rd reaction represents spliceosome_pre-mRNA complex releases mRNA and free spliceosome. The 4th to 6th reactions represent pre-mRNA and mRNA degradation by RNase. The above model was translated by Cellerator into 13 ODEs with 15 rate constants that describe the rates of change of 13 reactants involved in the model [Additional file 4: Mathematica™ codes and parameter values]. The forward rate constants (variable names with kf-prefix) and reverse rate constants (variable names with kr-prefix) were not available experimentally and approximated from experimental measurements (*Km *and *kcat*) of enzymes by Λ approximation method. The plausible values of the kinetic measurements (*Km *and *kcat*) are optimized from the quantitative time course measurements of pre-mRNA and spliced mRNA using *in vitro *RNA splicing assay for the Pol II and T7 polymerase as shown in Additional file 4.

### Analysis of the Nonlinear System using Systematic Perturbation

The rule of thumb for the Enzyme-Centric approach is that as more factors are introduced into the model, the more we can study how these factors affect the robustness of the system and why the system evolves to have these factors. However, the common bi-stability or bifurcation analysis requires reducing the complex nonlinear model (by assuming many variables as constants) to a simplified near-linear model with a few parameters. To maintain the biological complexity, an alternative way is to apply the systematic perturbation, which is commonly used in other contexts (e.g., bridge building, or automobile and airplane manufacturing) to test newly designed products through extensive computer simulations before prototyping. The goal of this task is to identify the regulatory proteins or controlling factors which are critical for the robustness and integrity of the biological system by iterating simulations with altered values of substrate/enzyme concentrations or kinetic constants. All enzymatic parameters including [*En*]total (total enzyme concentration), *Km *(the affinity of enzyme to substrate) and *kcat *(the rate of catalysis) were perturbed numerically.

## Abbreviations

BCAA: branched chain amino acids; *E. coli*:* Escherichia coli*; *S. typh*: *Salmonella typhimurium*; SM: Sulfometuron Methyl; Pol II: RNA polymerase II; MCA: metabolic control analysis; GMWC: Generalized *Monod, Wyman, Changeux *model; ODE: ordinary differential equation.

## Authors' contributions

C–RY developed kMech for mechanistic modeling, constructed and simulated the mathematical models of the metabolic network and RNA splicing pathway presented in the paper.

## Supplementary Material

Additional file 1 Mathematica™ codes and parameter values for the metabolic model. Click here for file

Additional file 2 Threonine Deaminase in *Salmonella typhimurium*. Click here for file

Additional file 3 The Lambda (Λ) approximation and Langevin equation. Click here for file

Additional file 4 Mathematica™ codes and parameter values for the RNA Splicing model. Click here for file
