# Supplementary material for: An enzyme-centric approach for modelling non-linear biological complexity
Source: BMC Syst Biol. 2008 Aug 1;2:70. doi: 10.1186/1752-0509-2-70 (PMC3146071; doi:10.1186/1752-0509-2-70)
Supplement: Additional file 1 — Mathematica™ codes and parameter values for the metabolic model. [file 1752-0509-2-70-S1.pdf]

# A Mathematical Model for the Flow of Carbon Through the Asparate, Threonine and the Branched Chain Amino Acid Pathways of *Escherichia coli* K12.

```
(* call Cellerator and kMech into Mathematica Kernel *)
<< cellerator.m;
<< kMech.m;
```

```
Cellerator™ 1.4.6 (30-July-2004) loaded 27-Aug-2005 17:01
  using Mathematica Version 5.0 for Microsoft Windows (June 11, 2003)
```

```
kMech is loaded
```

```
(* kMech Input of Enzyme Mechanisms for the Mathematical Modeling
  of Threonine (Thr), L-isoleucine (ILE), L-valine (VAL),
 and L-leucine (LEU) Biosynthesis *)

Asp$Thr$Synthesis = Union[

   {{{AKI} ==ASA==> {HDHI},   (modifiers: {{},{},{}})
     GMWC[cGMWC → cASA, LGMWC → LASA, nGMWC → nASA, KGMWC → {KmHDHIASA},
      kcatGMWC → kcat$AKI$HDHI]}},

   {{{HDHI} ==Asp==> {AKI},   (modifiers: {{},{},{}})
     GMWC[cGMWC → cAsp, LGMWC → LAsp, nGMWC → nAsp, KGMWC → {KmAKIAsp},
      kcatGMWC → kcat$HDHI$AKI]}},

   {{{Asp} ==AKI==> {AspP},   (modifiers: {{},{Thr},{}})
     GMWC[cGMWC → cAKI, LGMWC → LAKI, nGMWC → nAKI, KGMWC → {KmAKIAsp, KiAKIAspThr},
```

```
      kcatGMWC → kcat$AKI$Asp]}},
  Enz[{Asp, ATP} ⇄(AKII) {AspP, ADP}, BiBi[kfAKIIAsp, krAKIIAsp, kcat$AKII$Asp]],
  {{{Asp} ⟹(AKIII; {{},{Lys},{}}) {AspP},
    GMWC[cGMWC → cAKIII, LGMWC → LAKIII, nGMWC → nAKIII,
     KGMWC → {KmAKIIIAsp, KiAKIIIAspLys},  kcatGMWC → kcat$AKIII$Asp]}},
  Enz[{AspP, NADPH} ⇄(ASD) {ASA, NADP, Phosphate}, BiTri[kfASDAspP, krASDAspP, kcat$ASD$
  Enz[{ASA, NADP, Phosphate} ⇄(ASD) {AspP, NADPH}, TriBi[kfASDASA, krASDASA, kcat$ASD$ASA
  {{{ASA} ⟹(HDHI; {{},{Thr},{}}) {Hse},
    GMWC[cGMWC → cHDHI, LGMWC → LHDHI, nGMWC → nHDHI, KGMWC → {KmHDHIASA, KiHDHIASAT
     kcatGMWC → kcat$HDHI$ASA]}},
  {{ASA → ∅, kfASA}},
  {{Hse → ∅, kfHse}},
  Enz[{ASA, NADPH} ⇄(HDHII) {Hse, NADP}, BiBi[kfHDHIIASA, krHDHIIASA, kcat$HDHII$ASA]],
  Enz[{Hse, ATP} ⇄(HSK) {HseP, ADP}, BiBi[kfHSKHse, krHSKHse, kcat$HSK$Hse],
   CI[Thr, kfiHSKHseThr, kriHSKHseThr]],
  {{HseP ⇄(TS) Thr, kfTSHseP, krTSHseP, kcat$TS$HseP}}
 ];

ILESynthesis = Union[
  {{{Thr} ⟹(TDA; {{Val},{Ile}}) {aKB, NH3},
    GMWC[nGMWC → nTDA, cGMWC → cTDA, LGMWC → L0TDA, kcatGMWC → kcat$TDA,
     KGMWC → {KmThr, KaVal, KiIle}]}},
```

```
{{          aKB ⇄(KDC) propionylCoA, kfKDCaKB, krKDCaKB, kcat$KDC$aKB}},

Enz[{Pyr, aKB} ⇄(AHASI,AHASICH3CO) {CO2, aAHB}, PingPong[kfAHASIPyr, krAHASIPyr, kcat$AHASI$Pyr,
  kfAHASIaKB, krAHASIaKB, kcat$AHASI$aKB],
 NCI[Val, kfiAHASIPyrVal, kriAHASIPyrVal, kfiAHASIaKBVal, kriAHASIaKBVal,
  residualRateAHASIValaKB]],

Enz[{Pyr, aKB} ⇄(AHASII,AHASIICH3CO) {CO2, aAHB}, PingPong[kfAHASIIPyr, krAHASIIPyr, kcat$AHASII$Pyr,
  kfAHASIIaKB, krAHASIIaKB, kcat$AHASII$aKB]],

Enz[{Pyr, aKB} ⇄(AHASIII,AHASIIICH3CO) {CO2, aAHB}, PingPong[kfAHASIIIPyr, krAHASIIIPyr, kcat$AHASIII$Py
  kfAHASIIIaKB, krAHASIIIaKB, kcat$AHASIII$aKB],
 NCI[Val, kfiAHASIIIPyrVal, kriAHASIIIPyrVal, kfiAHASIIIaKBVal, kriAHASIIIaKBVal
  residualRateAHASIIIValaKB]],

Enz[{aAHB, NADPH} ⇄(IR) {aDMV, NADP}, BiBi[kfIRaAHB, krIRaAHB, kcat$IR$aAHB]],

{{aDMV ⇄(DAD) aKMV, kfDADaDMV, krDADaDMV, kcat$DAD$aDMV}},

Enz[{Glu, aKMV} ⇄(TB,TBNH2) {aKG, Ile}, PingPong[fkfTBGlu, fkrTBGlu, fkcat$TB$Glu, fkfTBaKM
  fkrTBaKMV, fkcat$TB$aKMV]],
Enz[{Ile, aKG} ⇄(TB,TBNH2) {aKMV, Glu}, PingPong[rkfTBIle, rkrTBIle, rkcat$TB$Ile, rkfTBaKG,
  rkrTBaKG, rkcat$TB$aKG]],
```

```
   {{exIle ⇄(LIVI) Ile, kfLIVIexIle, krLIVIexIle, kcat$LIVI$exIle}},
   {{exIle ⇄(LIVII) Ile, kfLIVIIexIle, krLIVIIexIle, kcat$LIVII$exIle}},

   {{aKMV → acetylCoA, kcat$aKMV}},

   {{Ile → protein, kcat$Ile}}];

VALSynthesis = Union[
   Enz[{Pyr, Pyr} ⇄(AHASI,AHASICH3CO) {CO2, aAL}, PingPong[kfAHASIPyr, krAHASIPyr, kcat$AHASI$Pyr,
     kfAHASIPyr2, krAHASIPyr2, kcat$AHASI$Pyr2],
    NCI[Val, kfiAHASIPyrVal, kriAHASIPyrVal, kfiAHASIPyr2Val, kriAHASIPyr2Val,
     residualRateAHASIValPyr2]],

   Enz[{Pyr, Pyr} ⇄(AHASII,AHASIICH3CO) {CO2, aAL}, PingPong[kfAHASIIPyr, krAHASIIPyr, kcat$AHASII$Pyr,
     kfAHASIIPyr2, krAHASIIPyr2, kcat$AHASII$Pyr2]],

   Enz[{Pyr, Pyr} ⇄(AHASIII,AHASIIICH3CO) {CO2, aAL}, PingPong[kfAHASIIIPyr, krAHASIIIPyr, kcat$AHASIII$Pyr
     kfAHASIIIPyr2, krAHASIIIPyr2, kcat$AHASIII$Pyr2],
    NCI[Val, kfiAHASIIIPyrVal, kriAHASIIIPyrVal, kfiAHASIIIPyr2Val, kriAHASIIIPyr2V
     residualRateAHASIIIValPyr2]],

   Enz[{aAL, NADPH} ⇄(IR) {aDHIV, NADP}, BiBi[kfIRaAL, krIRaAL, kcat$IR$aAL]],

   {{aDHIV ⇄(DAD) aKIV, kfDADaDHIV, krDADaDHIV, kcat$DAD$aDHIV}},
```

```
  Enz[{Ala, aKIV} ⇄(TC,TCNH2) {Pyr, Val}, PingPong[fkfTCAla, fkrTCAla, fkcat$TC$Ala, fkfTCaKI
    fkrTCaKIV, fkcat$TC$aKIV]],
  Enz[{Val, Pyr} ⇄(TC,TCNH2) {aKIV, Ala}, PingPong[rkfTCVal, rkrTCVal, rkcat$TC$Val, rkfTCPyr,
    rkrTCPyr, rkcat$TC$Pyr]],

  Enz[{Glu, aKIV} ⇄(TB,TBNH2) {aKG, Val}, PingPong[fkfTBGlu, fkrTBGlu, fkcat$TB$Glu, fkfTBaKI
    fkrTBaKIV, fkcat$TB$aKIV]],
  Enz[{Val, aKG} ⇄(TB,TBNH2) {aKIV, Glu}, PingPong[rkfTBVal, rkrTBVal, rkcat$TB$Val, rkfTBaKG,
    rkrTBaKG, rkcat$TB$aKG]],

  {{exVal ⇄(LIVI) Val, kfLIVIexVal, krLIVIexVal, kcat$LIVI$exVal}},
  {{exVal ⇄(LIVII) Val, kfLIVIIexVal, krLIVIIexVal, kcat$LIVII$exVal}},

  {{aKIV → pantothenate, kcat$aKIV}},

  {{Val → protein, kcat$Val}}];

LEUSynthesis = Union[
  Enz[{acetylCoA, aKIV} ⇄(IPMS,IPMSacetyl) {CoA, aIPM},
   PingPong[kfIPMSacetylCoA, krIPMSacetylCoA, kcat$IPMS$acetylCoA, kfIPMSaKIV,
    krIPMSaKIV, kcat$IPMS$aKIV], CI[Leu, kfiIPMSLeu, kriIPMSLeu],
   NCI[Leu, kfiIPMSacetylLeu, kriIPMSacetylLeu]],
```

```
       IPMI
{{aIPM ⇄ bIPM, kfIPMIaIPM, krIPMIaIPM, kcat$IPMI$aIPM}},
       IPMI
{{bIPM ⇄ aIPM, kfIPMIbIPM, krIPMIbIPM, kcat$IPMI$bIPM}},

                    IPMDH
Enz[{bIPM, NAD} ⇄ {aKIC, NADH}, BiBi[kfIPMDHbIPM, krIPMDHbIPM, kcat$IPMDH$bIPM]],

                TB,TBNH2
Enz[{Glu, aKIC} ⇄ {aKG, Leu}, PingPong[fkfTBGlu, fkrTBGlu, fkcat$TB$Glu, fkfTBaKI
  fkrTBaKIC, fkcat$TB$aKIC]],
                TB,TBNH2
Enz[{Leu, aKG} ⇄ {aKIC, Glu}, PingPong[rkfTBLeu, rkrTBLeu, rkcat$TB$Leu, rkfTBaKG,
  rkrTBaKG, rkcat$TB$aKG]],

{{aKIC → glutarylCoA, kcat$aKIC}},

        LIVI
{{exLeu ⇄ Leu, kfLIVIexLeu, krLIVIexLeu, kcat$LIVI$exLeu}},
        LIVII
{{exLeu ⇄ Leu, kfLIVIIexLeu, krLIVIIexLeu, kcat$LIVII$exLeu}},
         LS
{{exLeu ⇄ Leu, kfLSexLeu, krLSexLeu, kcat$LS$exLeu}},

{{Leu → protein, kcat$Leu}}];

THR$ILE$VAL$LEU$Synthesis = Union[Asp$Thr$Synthesis, ILESynthesis, VALSynthesis,
   LEUSynthesis];
```

```
(* Cellerator-interpreted Ordinary Differential Equations (ODEs) and
   Equations for the MWC Model and Substrate Generators . *)

interpret[THR$ILE$VAL$LEU$Synthesis];
```

```
(*
 Modifications for Substrate Generators:
   The generated ODEs require some modifications to include the
    constant substrate fluxes for the steady state simulation

  1. Substrate Generators:
         Set the first derivatives of the following substrates to 0
     to simulate constant flux,
 Asp'[t]==0, ATP'[t]==0, NADP'[t]==0, NADPH'[t]==0, Phosphate'[t]==0,
 Pyr'[t]==0, Glu'[t]==0, Ala'[t]==0, exIle′[t]==0, exVal′[t]==0,
 exLeu′[t]==0, acetylCoA′[t]==0, NAD′[t]==0,
 *)

{myODEs, myVars} =
 {{aAHB′[t] == -kfIRaAHB aAHB[t] IR[t] NADPH[t] + kcat$AHASI$aKB $Complex$aKB$AHASICH3C
     kcat$AHASI$aKB residualRateAHASIValaKB $Complex$aKB$AHASICH3CO$Val$[t] +
     kcat$AHASII$aKB $Complex$aKB$AHASIICH3CO$[t] +
     kcat$AHASIII$aKB $Complex$aKB$AHASIIICH3CO$[t] +
     kcat$AHASIII$aKB residualRateAHASIIIValaKB $Complex$aKB$AHASIIICH3CO$Val$[t] +
     krIRaAHB $Complex$IR$aAHB$NADPH$[t],
   aAL′[t] == -kfIRaAL aAL[t] IR[t] NADPH[t] + krIRaAL $Complex$IR$aAL$NADPH$[t] +
     kcat$AHASI$Pyr2 $Complex$Pyr$AHASICH3CO$[t] +
     kcat$AHASI$Pyr2 residualRateAHASIValPyr2 $Complex$Pyr$AHASICH3CO$Val$[t] +
     kcat$AHASII$Pyr2 $Complex$Pyr$AHASIICH3CO$[t] +
```

```
    kcat$AHASIII$Pyr2 $Complex$Pyr$AHASIIICH3CO$[t] +
    kcat$AHASIII$Pyr2 residualRateAHASIIIValPyr2 $Complex$Pyr$AHASIIICH3CO$Val$[t]
  acetylCoA′[t] == 0,
  aDHIV′[t] == -kfDADaDHIV aDHIV[t] DAD[t] + krDADaDHIV $Complex$aDHIV$DAD$[t] +
    kcat$IR$aAL $Complex$IR$aAL$NADPH$[t],
  aDMV′[t] == -kfDADaDMV aDMV[t] DAD[t] + krDADaDMV $Complex$aDMV$DAD$[t] +
    kcat$IR$aAHB $Complex$IR$aAHB$NADPH$[t],
  ADP′[t] == kcat$AKII$Asp $Complex$AKII$Asp$ATP$[t] + kcat$HSK$Hse $Complex$HSK$Hse$
  AHASI′[t] == -kfAHASIPyr AHASI[t] Pyr[t] - kfiAHASIPyrVal AHASI[t] Val[t] +
    kriAHASIPyrVal $Complex$AHASI$Val$[t] + kcat$AHASI$aKB $Complex$aKB$AHASICH3CO$
    kcat$AHASI$Pyr2 $Complex$Pyr$AHASICH3CO$[t] + krAHASIPyr $Complex$Pyr$AHASI$[t]
  AHASICH3CO′[t] == -kfAHASIaKB AHASICH3CO[t] aKB[t] - kfAHASIPyr2 AHASICH3CO[t] Pyr[t
    kfiAHASIaKBVal AHASICH3CO[t] Val[t] + kriAHASIaKBVal $Complex$AHASICH3CO$Val$[t
    krAHASIaKB $Complex$aKB$AHASICH3CO$[t] + krAHASIPyr2 $Complex$Pyr$AHASICH3CO$[t
    kcat$AHASI$Pyr $Complex$Pyr$AHASI$[t],
  AHASII′[t] == -kfAHASIIPyr AHASII[t] Pyr[t] + kcat$AHASII$aKB $Complex$aKB$AHASIICH
    kcat$AHASII$Pyr2 $Complex$Pyr$AHASIICH3CO$[t] + krAHASIIPyr $Complex$Pyr$AHASII
  AHASIICH3CO′[t] == -kfAHASIIaKB AHASIICH3CO[t] aKB[t] - kfAHASIIPyr2 AHASIICH3CO[t]
    krAHASIIaKB $Complex$aKB$AHASIICH3CO$[t] + krAHASIIPyr2 $Complex$Pyr$AHASIICH3C
    kcat$AHASII$Pyr $Complex$Pyr$AHASII$[t],
  AHASIII′[t] == -kfAHASIIIPyr AHASIII[t] Pyr[t] - kfiAHASIIIPyrVal AHASIII[t] Val[t]
    kriAHASIIIPyrVal $Complex$AHASIII$Val$[t] +
    kcat$AHASIII$aKB $Complex$aKB$AHASIIICH3CO$[t] +
    kcat$AHASIII$Pyr2 $Complex$Pyr$AHASIIICH3CO$[t] + krAHASIIIPyr $Complex$Pyr$AHA
  AHASIIICH3CO′[t] == -kfAHASIIIaKB AHASIIICH3CO[t] aKB[t] -
    kfAHASIIIPyr2 AHASIIICH3CO[t] Pyr[t] - kfiAHASIIIaKBVal AHASIIICH3CO[t] Val[t] +
    kriAHASIIIaKBVal $Complex$AHASIIICH3CO$Val$[t] +
    krAHASIIIaKB $Complex$aKB$AHASIIICH3CO$[t] +
    krAHASIIIPyr2 $Complex$Pyr$AHASIIICH3CO$[t] + kcat$AHASIII$Pyr $Complex$Pyr$AHA
```

$$\mathrm{aIPM}'[t] == -\mathrm{kfIPMIaIPM}\ \mathrm{aIPM}[t]\ \mathrm{IPMI}[t] + \mathrm{krIPMIaIPM}\ \$\mathrm{Complex}\$\mathrm{aIPM}\$\mathrm{IPMI}\$[t] + \mathrm{kcat}\$\mathrm{IPMS}\$\mathrm{aKIV}\ \$\mathrm{Complex}\$\mathrm{aKIV}\$\mathrm{IPMSacetyl}\$[t] + \mathrm{kcat}\$\mathrm{IPMI}\$\mathrm{bIPM}\ \$\mathrm{Complex}\$\mathrm{bIPM}\$\mathrm{IPMI}$$

$$\mathrm{aKB}'[t] == -\mathrm{kfAHASIaKB}\ \mathrm{AHASICH3CO}[t]\ \mathrm{aKB}[t] - \mathrm{kfAHASIIaKB}\ \mathrm{AHASIICH3CO}[t]\ \mathrm{aKB}[t] - \mathrm{kfAHASIIIaKB}\ \mathrm{AHASIIICH3CO}[t]\ \mathrm{aKB}[t] - \mathrm{kfKDCaKB}\ \mathrm{aKB}[t]\ \mathrm{KDC}[t] + \left(\mathrm{kcat}\$\mathrm{TDA}\ \mathrm{TDA}[t] \left(\frac{\mathrm{cTDA}\ \mathrm{L0TDA}\left(1+\frac{\mathrm{Ile}[t]}{\mathrm{KiIle}}\right)^{\mathrm{nTDA}} \mathrm{Thr}[t]\left(1+\frac{\mathrm{cTDA}\ \mathrm{Thr}[t]}{\mathrm{KmThr}}\right)^{-1+\mathrm{nTDA}}}{\mathrm{KmThr}} + \frac{\mathrm{Thr}[t]\left(1+\frac{\mathrm{Thr}[t]}{\mathrm{KmThr}}\right)^{-1+\mathrm{nTDA}}\left(1+\frac{\mathrm{Val}[t]}{\mathrm{KaVal}}\right)^{\mathrm{nTDA}}}{\mathrm{KmThr}}\right)\right) \Big/ \left(\mathrm{L0TDA}\left(1+\frac{\mathrm{Ile}[t]}{\mathrm{KiIle}}\right)^{\mathrm{nTDA}}\left(1+\frac{\mathrm{cTDA}\ \mathrm{Thr}[t]}{\mathrm{KmThr}}\right)^{\mathrm{nTDA}} + \left(1+\frac{\mathrm{Thr}[t]}{\mathrm{KmThr}}\right)^{\mathrm{nTDA}}\left(1+\frac{\mathrm{Val}[t]}{\mathrm{KaVal}}\right)^{\mathrm{nTDA}}\right) - \mathrm{kfAHASIaKB}\ \mathrm{aKB}[t]\ \$\mathrm{Complex}\$\mathrm{AHASICH3CO}\$\mathrm{Val}\$[t] - \mathrm{kfAHASIIIaKB}\ \mathrm{aKB}[t]\ \$\mathrm{Complex}\$\mathrm{AHASIIICH3CO}\$\mathrm{Val}\$[t] + \mathrm{krAHASIaKB}\ \$\mathrm{Complex}\$\mathrm{aKB}\$\mathrm{AHASICH3CO}\$[t] + \mathrm{krAHASIaKB}\ \$\mathrm{Complex}\$\mathrm{aKB}\$\mathrm{AHASICH3CO}\$\mathrm{Val} \ldots \mathrm{krAHASIIaKB}\ \$\mathrm{Complex}\$\mathrm{aKB}\$\mathrm{AHASIICH3CO}\$[t] + \mathrm{krAHASIIIaKB}\ \$\mathrm{Complex}\$\mathrm{aKB}\$\mathrm{AHASIIICH3} \ldots \mathrm{krAHASIIIaKB}\ \$\mathrm{Complex}\$\mathrm{aKB}\$\mathrm{AHASIIICH3CO}\$\mathrm{Val}\$[t] + \mathrm{krKDCaKB}\ \$\mathrm{Complex}\$\mathrm{aKB}\$\mathrm{KDC}\$[t],$$

$$\mathrm{aKG}'[t] == -\mathrm{rkfTBaKG}\ \mathrm{aKG}[t]\ \mathrm{TBNH2}[t] + \mathrm{rkrTBaKG}\ \$\mathrm{Complex}\$\mathrm{aKG}\$\mathrm{TBNH2}\$[t] + \mathrm{fkcat}\$\mathrm{TB}\$\mathrm{Glu}\ \$\mathrm{Complex}\$\mathrm{Glu}\$\mathrm{TB}\$[t],$$

$$\mathrm{AKI}'[t] == -\left(\mathrm{kcat}\$\mathrm{AKI}\$\mathrm{HDHI}\left(\frac{\mathrm{AKI}[t]\left(1+\frac{\mathrm{AKI}[t]}{\mathrm{KmHDHIASA}}\right)^{-1+\mathrm{nASA}}}{\mathrm{KmHDHIASA}} + \frac{\mathrm{cASA}\ \mathrm{LASA}\ \mathrm{AKI}[t]\left(1+\frac{\mathrm{cASA}\ \mathrm{AKI}[t]}{\mathrm{KmHDHIASA}}\right)^{-1+\mathrm{nA}\ldots}}{\mathrm{KmHDHIASA}} \ldots \mathrm{ASA}[t]\right) \Big/ \left(\left(\left(1+\frac{\mathrm{AKI}[t]}{\mathrm{KmHDHIASA}}\right)^{\mathrm{nASA}} + \mathrm{LASA}\left(1+\frac{\mathrm{cASA}\ \mathrm{AKI}[t]}{\mathrm{KmHDHIASA}}\right)^{\mathrm{nASA}}\right) + \right.$$

$$\left(\text{kcat\$HDHI\$AKI}\ \text{Asp}[t]\left(\frac{\text{HDHI}[t]\left(1+\frac{\text{HDHI}[t]}{\text{KmAKIAsp}}\right)^{-1+\text{nAsp}}}{\text{KmAKIAsp}}+\frac{\text{cAsp LAsp HDHI}[t]\left(1+\frac{\text{cAsp HDHI}[t]}{\text{KmAKIAsp}}\right)^{-1+\text{nAsp}}}{\text{KmAKIAsp}}\right)\right)\Big/$$

$$\left(\left(1+\frac{\text{HDHI}[t]}{\text{KmAKIAsp}}\right)^{\text{nAsp}}+\text{LAsp}\left(1+\frac{\text{cAsp HDHI}[t]}{\text{KmAKIAsp}}\right)^{\text{nAsp}}\right),$$

```
aKIC′[t] == -kcat$aKIC aKIC[t] - fkfTBaKIC aKIC[t] TBNH2[t] +
  fkrTBaKIC $Complex$aKIC$TBNH2$[t] + kcat$IPMDH$bIPM $Complex$IPMDH$bIPM$NAD$[t]
  rkcat$TB$Leu $Complex$Leu$TB$[t],
AKII′[t] == -kfAKIIAsp AKII[t] Asp[t] ATP[t] + kcat$AKII$Asp $Complex$AKII$Asp$ATP$[
  krAKIIAsp $Complex$AKII$Asp$ATP$[t], AKIII′[t] == 0,
aKIV′[t] == -kcat$aKIV aKIV[t] - kfIPMSaKIV aKIV[t] IPMSacetyl[t] -
  fkfTBaKIV aKIV[t] TBNH2[t] - fkfTCaKIV aKIV[t] TCNH2[t] +
  kcat$DAD$aDHIV $Complex$aDHIV$DAD$[t] + krIPMSaKIV $Complex$aKIV$IPMSacetyl$[t]
  krIPMSaKIV $Complex$aKIV$IPMSacetyl$Leu$[t] + fkrTBaKIV $Complex$aKIV$TBNH2$[t]
  fkrTCaKIV $Complex$aKIV$TCNH2$[t] - kfIPMSaKIV aKIV[t] $Complex$IPMSacetyl$Leu$
  rkcat$TB$Val $Complex$Val$TB$[t] + rkcat$TC$Val $Complex$Val$TC$[t],
aKMV′[t] == -kcat$aKMV aKMV[t] - fkfTBaKMV aKMV[t] TBNH2[t] +
  kcat$DAD$aDMV $Complex$aDMV$DAD$[t] + fkrTBaKMV $Complex$aKMV$TBNH2$[t] +
  rkcat$TB$Ile $Complex$Ile$TB$[t],
Ala′[t] == 0,
ASA′[t] == -kfASA ASA[t] - kfHDHIIASA ASA[t] HDHII[t] NADPH[t] -
  kfASDASA ASA[t] ASD[t] NADP[t] Phosphate[t] -
   (kcat$HDHI$ASA HDHI[t]
```

$$\left(\frac{\mathrm{ASA}[t]\left(1+\frac{\mathrm{ASA}[t]}{\mathrm{KmHDHIASA}}\right)^{-1+\mathrm{nHDHI}}}{\mathrm{KmHDHIASA}}+\right.$$

$$\left.\left.\left.\frac{\mathrm{cHDHI}\;\mathrm{LHDHI}\;\mathrm{ASA}[t]\left(1+\frac{\mathrm{cHDHI}\;\mathrm{ASA}[t]}{\mathrm{KmHDHIASA}}\right)^{-1+\mathrm{nHDHI}}\left(1+\frac{\mathrm{Thr}[t]}{\mathrm{KiHDHIASAThr}}\right)^{\mathrm{nHDHI}}}{\mathrm{KmHDHIASA}}\right)\right)\right)\Big/$$

$$\left(\left(1+\frac{\mathrm{ASA}[t]}{\mathrm{KmHDHIASA}}\right)^{\mathrm{nHDHI}}+\mathrm{LHDHI}\left(1+\frac{\mathrm{cHDHI}\;\mathrm{ASA}[t]}{\mathrm{KmHDHIASA}}\right)^{\mathrm{nHDHI}}\left(1+\frac{\mathrm{Thr}[t]}{\mathrm{KiHDHIASAThr}}\right)^{\mathrm{nHDHI}}\right)+$$

$$\mathrm{krASDASA}\;\mathrm{\$Complex\$ASD\$ASA\$NADP\$Phosphate\$}[t]+$$

$$\mathrm{kcat\$ASD\$AspP}\;\mathrm{\$Complex\$ASD\$AspP\$NADPH\$}[t]+\mathrm{krHDHIIASA}\;\mathrm{\$Complex\$HDHII\$ASA\$NADP}$$

$$\mathrm{ASD}'[t]==-\mathrm{kfASDAspP}\;\mathrm{ASD}[t]\;\mathrm{AspP}[t]\;\mathrm{NADPH}[t]-\mathrm{kfASDASA}\;\mathrm{ASA}[t]\;\mathrm{ASD}[t]\;\mathrm{NADP}[t]\;\mathrm{Phosph}$$

$$\mathrm{kcat\$ASD\$ASA}\;\mathrm{\$Complex\$ASD\$ASA\$NADP\$Phosphate\$}[t]+$$

$$\mathrm{krASDASA}\;\mathrm{\$Complex\$ASD\$ASA\$NADP\$Phosphate\$}[t]+$$

$$\mathrm{kcat\$ASD\$AspP}\;\mathrm{\$Complex\$ASD\$AspP\$NADPH\$}[t]+\mathrm{krASDAspP}\;\mathrm{\$Complex\$ASD\$AspP\$NADPH\$}$$

$$\mathrm{Asp}'[t]==0,$$

$$\mathrm{AspP}'[t]==$$

$$\left(\mathrm{kcat\$AKIII\$Asp}\;\mathrm{AKIII}[t]\right.$$

$$\left(\frac{\mathrm{Asp}[t]\left(1+\frac{\mathrm{Asp}[t]}{\mathrm{KmAKIIIAsp}}\right)^{-1+\mathrm{nAKIII}}}{\mathrm{KmAKIIIAsp}}+\frac{1}{\mathrm{KmAKIIIAsp}}\right.$$

$$\left.\left(\mathrm{cAKIII}\;\mathrm{LAKIII}\;\mathrm{Asp}[t]\left(1+\frac{\mathrm{cAKIII}\;\mathrm{Asp}[t]}{\mathrm{KmAKIIIAsp}}\right)^{-1+\mathrm{nAKIII}}\left(1+\frac{\mathrm{Lys}[t]}{\mathrm{KiAKIIIAspLys}}\right)^{\mathrm{nAKIII}}\right)\right)$$

$$\left(\left(1+\frac{\mathrm{Asp}[t]}{\mathrm{KmAKIIIAsp}}\right)^{\mathrm{nAKIII}}+\mathrm{LAKIII}\left(1+\frac{\mathrm{cAKIII}\;\mathrm{Asp}[t]}{\mathrm{KmAKIIIAsp}}\right)^{\mathrm{nAKIII}}\left(1+\frac{\mathrm{Lys}[t]}{\mathrm{KiAKIIIAspLys}}\right)^{\mathrm{nAKII}}\right.$$

$$\mathrm{kfASDAspP\ ASD[t]\ AspP[t]\ NADPH[t]} +$$

$$\left(\mathrm{kcat\$AKI\$Asp\ AKI[t]}\left(\frac{\mathrm{Asp[t]}\left(1+\frac{\mathrm{Asp[t]}}{\mathrm{KmAKIAsp}}\right)^{-1+\mathrm{nAKI}}}{\mathrm{KmAKIAsp}}+\frac{\mathrm{cAKI\ LAKI\ Asp[t]}\left(1+\frac{\mathrm{cAKI\ Asp[t]}}{\mathrm{KmAKIAsp}}\right)^{-1+\mathrm{nAKI}}\left(1+\frac{\mathrm{Thr[t]}}{\mathrm{KiAKIAspThr}}\right)^{\mathrm{nAKI}}}{\mathrm{KmAKIAsp}}\right)\right)\Big/$$

$$\left(\left(1+\frac{\mathrm{Asp[t]}}{\mathrm{KmAKIAsp}}\right)^{\mathrm{nAKI}}+\mathrm{LAKI}\left(1+\frac{\mathrm{cAKI\ Asp[t]}}{\mathrm{KmAKIAsp}}\right)^{\mathrm{nAKI}}\left(1+\frac{\mathrm{Thr[t]}}{\mathrm{KiAKIAspThr}}\right)^{\mathrm{nAKI}}\right)+$$

$$\mathrm{kcat\$AKII\$Asp\ \$Complex\$AKII\$Asp\$ATP\$[t]} +$$
$$\mathrm{kcat\$ASD\$ASA\ \$Complex\$ASD\$ASA\$NADP\$Phosphate\$[t]} +$$
$$\mathrm{krASDAspP\ \$Complex\$ASD\$AspP\$NADPH\$[t]},$$

$$\mathrm{ATP'[t]} == 0,$$

$$\mathrm{bIPM'[t]} == -\mathrm{kfIPMIbIPM\ bIPM[t]\ IPMI[t]} - \mathrm{kfIPMDHbIPM\ bIPM[t]\ IPMDH[t]\ NAD[t]} +$$
$$\mathrm{kcat\$IPMI\$aIPM\ \$Complex\$aIPM\$IPMI\$[t]} + \mathrm{krIPMIbIPM\ \$Complex\$bIPM\$IPMI\$[t]} +$$
$$\mathrm{krIPMDHbIPM\ \$Complex\$IPMDH\$bIPM\$NAD\$[t]},$$

$$\mathrm{CO2'[t]} == \mathrm{kcat\$AHASIII\$Pyr\ \$Complex\$Pyr\$AHASIII\$[t]} +$$
$$\mathrm{kcat\$AHASIII\$Pyr\ residualRateAHASIIIValaKB\ \$Complex\$Pyr\$AHASIII\$Val\$[t]} +$$
$$\mathrm{kcat\$AHASII\$Pyr\ \$Complex\$Pyr\$AHASII\$[t]} + \mathrm{kcat\$AHASI\$Pyr\ \$Complex\$Pyr\$AHASI\$[t]}$$
$$\mathrm{kcat\$AHASI\$Pyr\ residualRateAHASIValaKB\ \$Complex\$Pyr\$AHASI\$Val\$[t]},$$

$$\mathrm{CoA'[t]} == \mathrm{kcat\$IPMS\$acetylCoA\ \$Complex\$acetylCoA\$IPMS\$[t]},$$

$$\mathrm{DAD'[t]} == -\mathrm{kfDADaDHIV\ aDHIV[t]\ DAD[t]} - \mathrm{kfDADaDMV\ aDMV[t]\ DAD[t]} +$$
$$\mathrm{kcat\$DAD\$aDHIV\ \$Complex\$aDHIV\$DAD\$[t]} + \mathrm{krDADaDHIV\ \$Complex\$aDHIV\$DAD\$[t]} +$$
$$\mathrm{kcat\$DAD\$aDMV\ \$Complex\$aDMV\$DAD\$[t]} + \mathrm{krDADaDMV\ \$Complex\$aDMV\$DAD\$[t]},$$

$$\mathrm{exIle'[t]} == 0,$$

```
exLeu′[t] == 0,
exVal′[t] == 0,
Glu′[t] == 0,
glutarylCoA′[t] == kcat$aKIC aKIC[t],
HDHI′[t] ==
 (kcat$AKI$HDHI (AKI[t] (1 + AKI[t]/KmHDHIASA)^(-1+nASA)/KmHDHIASA + cASA LASA AKI[t] (1 + cASA AKI[t]/KmHDHIASA)^(-1+nASA
     ASA[t]) / ((1 + AKI[t]/KmHDHIASA)^nASA + LASA (1 + cASA AKI[t]/KmHDHIASA)^nASA) -
  (kcat$HDHI$AKI Asp[t]
     (HDHI[t] (1 + HDHI[t]/KmAKIAsp)^(-1+nAsp)/KmAKIAsp + cAsp LAsp HDHI[t] (1 + cAsp HDHI[t]/KmAKIAsp)^(-1+nAsp)/KmAKIAsp)) /
   ((1 + HDHI[t]/KmAKIAsp)^nAsp + LAsp (1 + cAsp HDHI[t]/KmAKIAsp)^nAsp),
HDHII′[t] == -kfHDHIIASA ASA[t] HDHII[t] NADPH[t] +
  kcat$HDHII$ASA $Complex$HDHII$ASA$NADPH$[t] + krHDHIIASA $Complex$HDHII$ASA$NAI
Hse′[t] == -kfHse Hse[t] - kfHSKHse ATP[t] Hse[t] HSK[t] +
  (kcat$HDHI$ASA HDHI[t]
```

$$\left(\frac{\mathrm{ASA}[t]\left(1+\frac{\mathrm{ASA}[t]}{\mathrm{KmHDHIASA}}\right)^{-1+\mathrm{nHDHI}}}{\mathrm{KmHDHIASA}}+\right.$$

$$\left.\left.\frac{\mathrm{cHDHI}\ \mathrm{LHDHI}\ \mathrm{ASA}[t]\left(1+\frac{\mathrm{cHDHI}\ \mathrm{ASA}[t]}{\mathrm{KmHDHIASA}}\right)^{-1+\mathrm{nHDHI}}\left(1+\frac{\mathrm{Thr}[t]}{\mathrm{KiHDHIASAThr}}\right)^{\mathrm{nHDHI}}}{\mathrm{KmHDHIASA}}\right)\right)\Big/$$

$$\left(\left(1+\frac{\mathrm{ASA}[t]}{\mathrm{KmHDHIASA}}\right)^{\mathrm{nHDHI}}+\mathrm{LHDHI}\left(1+\frac{\mathrm{cHDHI}\ \mathrm{ASA}[t]}{\mathrm{KmHDHIASA}}\right)^{\mathrm{nHDHI}}\left(1+\frac{\mathrm{Thr}[t]}{\mathrm{KiHDHIASAThr}}\right)^{\mathrm{nHDHI}}\right)+$$

```
   kcat$HDHII$ASA $Complex$HDHII$ASA$NADPH$[t] + krHSKHse $Complex$HSK$Hse$ATP$[t]
HseP′[t] == -kfTSHseP HseP[t] TS[t] + krTSHseP $Complex$HseP$TS$[t] +
   kcat$HSK$Hse $Complex$HSK$Hse$ATP$[t],
HSK′[t] == -kfHSKHse ATP[t] Hse[t] HSK[t] - kfiHSKHseThr HSK[t] Thr[t] +
   kcat$HSK$Hse $Complex$HSK$Hse$ATP$[t] + krHSKHse $Complex$HSK$Hse$ATP$[t] +
   kriHSKHseThr $Complex$HSK$Thr$[t],
Ile′[t] == -kcat$Ile Ile[t] - rkfTBIle Ile[t] TB[t] + fkcat$TB$aKMV $Complex$aKMV$TBN
   kcat$LIVII$exIle $Complex$exIle$LIVII$[t] + kcat$LIVI$exIle $Complex$exIle$LIVI
   rkrTBIle $Complex$Ile$TB$[t],
IPMDH′[t] == -kfIPMDHbIPM bIPM[t] IPMDH[t] NAD[t] +
   kcat$IPMDH$bIPM $Complex$IPMDH$bIPM$NAD$[t] + krIPMDHbIPM $Complex$IPMDH$bIPM$N
IPMI′[t] == -kfIPMIaIPM aIPM[t] IPMI[t] - kfIPMIbIPM bIPM[t] IPMI[t] +
   kcat$IPMI$aIPM $Complex$aIPM$IPMI$[t] + krIPMIaIPM $Complex$aIPM$IPMI$[t] +
   kcat$IPMI$bIPM $Complex$bIPM$IPMI$[t] + krIPMIbIPM $Complex$bIPM$IPMI$[t],
IPMS′[t] == -kfIPMSacetylCoA acetylCoA[t] IPMS[t] - kfiIPMSLeu IPMS[t] Leu[t] +
   krIPMSacetylCoA $Complex$acetylCoA$IPMS$[t] +
   kcat$IPMS$aKIV $Complex$aKIV$IPMSacetyl$[t] + kriIPMSLeu $Complex$IPMS$Leu$[t],
IPMSacetyl′[t] == -kfIPMSaKIV aKIV[t] IPMSacetyl[t] - kfiIPMSacetylLeu IPMSacetyl[t
   kcat$IPMS$acetylCoA $Complex$acetylCoA$IPMS$[t] +
   krIPMSaKIV $Complex$aKIV$IPMSacetyl$[t] + kriIPMSacetylLeu $Complex$IPMSacetylS
IR′[t] == -kfIRaAHB aAHB[t] IR[t] NADPH[t] - kfIRaAL aAL[t] IR[t] NADPH[t] +
```

```
    kcat$IR$aAHB $Complex$IR$aAHB$NADPH$[t] + krIRaAHB $Complex$IR$aAHB$NADPH$[t] +
    kcat$IR$aAL $Complex$IR$aAL$NADPH$[t] + krIRaAL $Complex$IR$aAL$NADPH$[t],
  KDC′[t] == -kfKDCaKB aKB[t] KDC[t] + kcat$KDC$aKB $Complex$aKB$KDC$[t] +
    krKDCaKB $Complex$aKB$KDC$[t],
  Leu′[t] == -kcat$Leu Leu[t] - kfiIPMSLeu IPMS[t] Leu[t] -
    kfiIPMSacetylLeu IPMSacetyl[t] Leu[t] - rkfTBLeu Leu[t] TB[t] +
    fkcat$TB$aKIC $Complex$aKIC$TBNH2$[t] -
    kfiIPMSacetylLeu Leu[t] $Complex$aKIV$IPMSacetyl$[t] +
    kriIPMSacetylLeu $Complex$aKIV$IPMSacetyl$Leu$[t] +
    kcat$LIVII$exLeu $Complex$exLeu$LIVII$[t] + kcat$LIVI$exLeu $Complex$exLeu$LIVI
    kcat$LS$exLeu $Complex$exLeu$LS$[t] + kriIPMSacetylLeu $Complex$IPMSacetyl$Leu$
    kriIPMSLeu $Complex$IPMS$Leu$[t] + rkrTBLeu $Complex$Leu$TB$[t],
  LIVI′[t] == -kfLIVIexIle exIle[t] LIVI[t] - kfLIVIexLeu exLeu[t] LIVI[t] -
    kfLIVIexVal exVal[t] LIVI[t] + kcat$LIVI$exIle $Complex$exIle$LIVI$[t] +
    krLIVIexIle $Complex$exIle$LIVI$[t] + kcat$LIVI$exLeu $Complex$exLeu$LIVI$[t] +
    krLIVIexLeu $Complex$exLeu$LIVI$[t] + kcat$LIVI$exVal $Complex$exVal$LIVI$[t] +
    krLIVIexVal $Complex$exVal$LIVI$[t],
  LIVII′[t] == -kfLIVIIexIle exIle[t] LIVII[t] - kfLIVIIexLeu exLeu[t] LIVII[t] -
    kfLIVIIexVal exVal[t] LIVII[t] + kcat$LIVII$exIle $Complex$exIle$LIVII$[t] +
    krLIVIIexIle $Complex$exIle$LIVII$[t] + kcat$LIVII$exLeu $Complex$exLeu$LIVII$[
    krLIVIIexLeu $Complex$exLeu$LIVII$[t] + kcat$LIVII$exVal $Complex$exVal$LIVII$[
    krLIVIIexVal $Complex$exVal$LIVII$[t],
  LS′[t] == -kfLSexLeu exLeu[t] LS[t] + kcat$LS$exLeu $Complex$exLeu$LS$[t] +
    krLSexLeu $Complex$exLeu$LS$[t],
  Lys′[t] == 0,
  NAD′[t] == 0,
  NADH′[t] == kcat$IPMDH$bIPM $Complex$IPMDH$bIPM$NAD$[t],
  NADP′[t] == 0,
  NADPH′[t] == 0,
```

```
NH3′[t] == 
 (kcat$TDA TDA[t] ((cTDA L0TDA (1 + Ile[t]/KiIle)^nTDA Thr[t] (1 + (cTDA Thr[t])/KmThr)^(-1+nTDA))/KmThr + 
       (Thr[t] (1 + Thr[t]/KmThr)^(-1+nTDA) (1 + Val[t]/KaVal)^nTDA)/KmThr)) / 
   (L0TDA (1 + Ile[t]/KiIle)^nTDA (1 + (cTDA Thr[t])/KmThr)^nTDA + (1 + Thr[t]/KmThr)^nTDA (1 + Val[t]/KaVal)^nTDA),
pantothenate′[t] == kcat$aKIV aKIV[t],
Phosphate′[t] == 0,
propionylCoA′[t] == kcat$KDC$aKB $Complex$aKB$KDC$[t],
protein′[t] == kcat$Ile Ile[t] + kcat$Leu Leu[t] + kcat$Val Val[t],
Pyr′[t] == 0,
TB′[t] == -fkfTBGlu Glu[t] TB[t] - rkfTBIle Ile[t] TB[t] - rkfTBLeu Leu[t] TB[t] - 
   rkfTBVal TB[t] Val[t] + rkcat$TB$aKG $Complex$aKG$TBNH2$[t] + 
   fkcat$TB$aKIC $Complex$aKIC$TBNH2$[t] + fkcat$TB$aKIV $Complex$aKIV$TBNH2$[t] + 
   fkcat$TB$aKMV $Complex$aKMV$TBNH2$[t] + fkrTBGlu $Complex$Glu$TB$[t] + 
   rkrTBIle $Complex$Ile$TB$[t] + rkrTBLeu $Complex$Leu$TB$[t] + 
   rkrTBVal $Complex$Val$TB$[t],
TBNH2′[t] == -rkfTBaKG aKG[t] TBNH2[t] - fkfTBaKIC aKIC[t] TBNH2[t] - 
   fkfTBaKIV aKIV[t] TBNH2[t] - fkfTBaKMV aKMV[t] TBNH2[t] + rkrTBaKG $Complex$aKG$T[illegible]
   fkrTBaKIC $Complex$aKIC$TBNH2$[t] + fkrTBaKIV $Complex$aKIV$TBNH2$[t] + 
   fkrTBaKMV $Complex$aKMV$TBNH2$[t] + fkcat$TB$Glu $Complex$Glu$TB$[t] + 
   rkcat$TB$Ile $Complex$Ile$TB$[t] + rkcat$TB$Leu $Complex$Leu$TB$[t] + 
   rkcat$TB$Val $Complex$Val$TB$[t],
TC′[t] == -fkfTCAla Ala[t] TC[t] - rkfTCVal TC[t] Val[t] + 
   fkcat$TC$aKIV $Complex$aKIV$TCNH2$[t] + fkrTCAla $Complex$Ala$TC$[t] + 
   rkcat$TC$Pyr $Complex$Pyr$TCNH2$[t] + rkrTCVal $Complex$Val$TC$[t],
```

```
TCNH2′[t] == -fkfTCaKIV aKIV[t] TCNH2[t] - rkfTCPyr Pyr[t] TCNH2[t] +
  fkrTCaKIV $Complex$aKIV$TCNH2$[t] + fkcat$TC$Ala $Complex$Ala$TC$[t] +
  rkrTCPyr $Complex$Pyr$TCNH2$[t] + rkcat$TC$Val $Complex$Val$TC$[t], TDA′[t] == 0,
Thr′[t] == -kfiHSKHseThr HSK[t] Thr[t] -
```

$$\left(\text{kcat\$TDA TDA[t]}\left(\frac{\text{cTDA L0TDA}\left(1+\frac{\text{Ile[t]}}{\text{KiIle}}\right)^{\text{nTDA}}\text{Thr[t]}\left(1+\frac{\text{cTDA Thr[t]}}{\text{KmThr}}\right)^{-1+\text{nTDA}}}{\text{KmThr}}+\frac{\text{Thr[t]}\left(1+\frac{\text{Thr[t]}}{\text{KmThr}}\right)^{-1+\text{nTDA}}\left(1+\frac{\text{Val[t]}}{\text{KaVal}}\right)^{\text{nTDA}}}{\text{KmThr}}\right)\right)\Big/$$

$$\left(\text{L0TDA}\left(1+\frac{\text{Ile[t]}}{\text{KiIle}}\right)^{\text{nTDA}}\left(1+\frac{\text{cTDA Thr[t]}}{\text{KmThr}}\right)^{\text{nTDA}}+\left(1+\frac{\text{Thr[t]}}{\text{KmThr}}\right)^{\text{nTDA}}\left(1+\frac{\text{Val[t]}}{\text{KaVal}}\right)^{\text{nTDA}}\right)\ [illegible]$$

```
  kcat$TS$HseP $Complex$HseP$TS$[t] + kriHSKHseThr $Complex$HSK$Thr$[t],
TS′[t] == -kfTSHseP HseP[t] TS[t] + kcat$TS$HseP $Complex$HseP$TS$[t] +
  krTSHseP $Complex$HseP$TS$[t],
Val′[t] == -kcat$Val Val[t] - kfiAHASIPyrVal AHASI[t] Val[t] -
  kfiAHASIaKBVal AHASICH3CO[t] Val[t] - kfiAHASIIIPyrVal AHASIII[t] Val[t] -
  kfiAHASIIIaKBVal AHASIIICH3CO[t] Val[t] - rkfTBVal TB[t] Val[t] - rkfTCVal TC[t] V[illegible]
  kriAHASIaKBVal $Complex$AHASICH3CO$Val$[t] +
  kriAHASIIIaKBVal $Complex$AHASIIICH3CO$Val$[t] +
  kriAHASIIIPyrVal $Complex$AHASIII$Val$[t] + kriAHASIPyrVal $Complex$AHASI$Val$[illegible]
  kfiAHASIaKBVal Val[t] $Complex$aKB$AHASICH3CO$[t] +
  kriAHASIaKBVal $Complex$aKB$AHASICH3CO$Val$[t] -
  kfiAHASIIIaKBVal Val[t] $Complex$aKB$AHASIIICH3CO$[t] +
  kriAHASIIIaKBVal $Complex$aKB$AHASIIICH3CO$Val$[t] +
  fkcat$TB$aKIV $Complex$aKIV$TBNH2$[t] + fkcat$TC$aKIV $Complex$aKIV$TCNH2$[t] +
  kcat$LIVII$exVal $Complex$exVal$LIVII$[t] + kcat$LIVI$exVal $Complex$exVal$LIVI[illegible]
  kfiAHASIPyr2Val Val[t] $Complex$Pyr$AHASICH3CO$[t] +
  kriAHASIPyr2Val $Complex$Pyr$AHASICH3CO$Val$[t] -
```

```
    kfiAHASIIIPyr2Val Val[t] $Complex$Pyr$AHASIIICH3CO$[t] +
    kriAHASIIIPyr2Val $Complex$Pyr$AHASIIICH3CO$Val$[t] -
    kfiAHASIIIPyrVal Val[t] $Complex$Pyr$AHASIII$[t] +
    kriAHASIIIPyrVal $Complex$Pyr$AHASIII$Val$[t] -
    kfiAHASIPyrVal Val[t] $Complex$Pyr$AHASI$[t] + kriAHASIPyrVal $Complex$Pyr$AHAS
    rkrTBVal $Complex$Val$TB$[t] + rkrTCVal $Complex$Val$TC$[t],
  $Complex$acetylCoA$IPMS$′[t] == kfIPMSacetylCoA acetylCoA[t] IPMS[t] -
    kcat$IPMS$acetylCoA $Complex$acetylCoA$IPMS$[t] -
    krIPMSacetylCoA $Complex$acetylCoA$IPMS$[t],
  $Complex$aDHIV$DAD$′[t] == kfDADaDHIV aDHIV[t] DAD[t] -
    kcat$DAD$aDHIV $Complex$aDHIV$DAD$[t] - krDADaDHIV $Complex$aDHIV$DAD$[t],
  $Complex$aDMV$DAD$′[t] == kfDADaDMV aDMV[t] DAD[t] - kcat$DAD$aDMV $Complex$aDMV$DA
    krDADaDMV $Complex$aDMV$DAD$[t],
  $Complex$AHASICH3CO$Val$′[t] == kfiAHASIaKBVal AHASICH3CO[t] Val[t] -
    kriAHASIaKBVal $Complex$AHASICH3CO$Val$[t] -
    kfAHASIaKB aKB[t] $Complex$AHASICH3CO$Val$[t] -
    kfAHASIPyr2 Pyr[t] $Complex$AHASICH3CO$Val$[t] +
    krAHASIaKB $Complex$aKB$AHASICH3CO$Val$[t] +
    kcat$AHASI$aKB residualRateAHASIValaKB $Complex$aKB$AHASICH3CO$Val$[t] +
    krAHASIPyr2 $Complex$Pyr$AHASICH3CO$Val$[t] +
    kcat$AHASI$Pyr2 residualRateAHASIValPyr2 $Complex$Pyr$AHASICH3CO$Val$[t],
  $Complex$AHASIIICH3CO$Val$′[t] ==
   kfiAHASIIIaKBVal AHASIIICH3CO[t] Val[t] - kriAHASIIIaKBVal $Complex$AHASIIICH3CO
    kfAHASIIIaKB aKB[t] $Complex$AHASIIICH3CO$Val$[t] -
    kfAHASIIIPyr2 Pyr[t] $Complex$AHASIIICH3CO$Val$[t] +
    krAHASIIIaKB $Complex$aKB$AHASIIICH3CO$Val$[t] +
    kcat$AHASIII$aKB residualRateAHASIIIValaKB $Complex$aKB$AHASIIICH3CO$Val$[t] +
    krAHASIIIPyr2 $Complex$Pyr$AHASIIICH3CO$Val$[t] +
    kcat$AHASIII$Pyr2 residualRateAHASIIIValPyr2 $Complex$Pyr$AHASIIICH3CO$Val$[t
```

```
$Complex$AHASIII$Val$′[t] == kfiAHASIIIPyrVal AHASIII[t] Val[t] -
  kriAHASIIIPyrVal $Complex$AHASIII$Val$[t] - kfAHASIIIPyr Pyr[t] $Complex$AHASII
  krAHASIIIPyr $Complex$Pyr$AHASIII$Val$[t] +
  kcat$AHASIII$Pyr residualRateAHASIIIValaKB $Complex$Pyr$AHASIII$Val$[t],
$Complex$AHASI$Val$′[t] == kfiAHASIPyrVal AHASI[t] Val[t] -
  kriAHASIPyrVal $Complex$AHASI$Val$[t] - kfAHASIPyr Pyr[t] $Complex$AHASI$Val$[t
  krAHASIPyr $Complex$Pyr$AHASI$Val$[t] +
  kcat$AHASI$Pyr residualRateAHASIValaKB $Complex$Pyr$AHASI$Val$[t],
$Complex$aIPM$IPMI$′[t] == kfIPMIaIPM aIPM[t] IPMI[t] -
  kcat$IPMI$aIPM $Complex$aIPM$IPMI$[t] - krIPMIaIPM $Complex$aIPM$IPMI$[t],
$Complex$aKB$AHASICH3CO$′[t] == kfAHASIaKB AHASICH3CO[t] aKB[t] -
  kcat$AHASI$aKB $Complex$aKB$AHASICH3CO$[t] - krAHASIaKB $Complex$aKB$AHASICH3C
  kfiAHASIaKBVal Val[t] $Complex$aKB$AHASICH3CO$[t] +
  kriAHASIaKBVal $Complex$aKB$AHASICH3CO$Val$[t],
$Complex$aKB$AHASICH3CO$Val$′[t] ==
 kfAHASIaKB aKB[t] $Complex$AHASICH3CO$Val$[t] +
  kfiAHASIaKBVal Val[t] $Complex$aKB$AHASICH3CO$[t] -
  krAHASIaKB $Complex$aKB$AHASICH3CO$Val$[t] -
  kriAHASIaKBVal $Complex$aKB$AHASICH3CO$Val$[t] -
  kcat$AHASI$aKB residualRateAHASIValaKB $Complex$aKB$AHASICH3CO$Val$[t],
$Complex$aKB$AHASIICH3CO$′[t] ==
 kfAHASIIaKB AHASIICH3CO[t] aKB[t] - kcat$AHASII$aKB $Complex$aKB$AHASIICH3CO$[t]
  krAHASIIaKB $Complex$aKB$AHASIICH3CO$[t],
$Complex$aKB$AHASIIICH3CO$′[t] ==
 kfAHASIIIaKB AHASIIICH3CO[t] aKB[t] - kcat$AHASIII$aKB $Complex$aKB$AHASIIICH3CO
  krAHASIIIaKB $Complex$aKB$AHASIIICH3CO$[t] -
  kfiAHASIIIaKBVal Val[t] $Complex$aKB$AHASIIICH3CO$[t] +
  kriAHASIIIaKBVal $Complex$aKB$AHASIIICH3CO$Val$[t],
$Complex$aKB$AHASIIICH3CO$Val$′[t] ==
```

```
 kfAHASIIIaKB aKB[t] $Complex$AHASIIICH3CO$Val$[t] +
  kfiAHASIIIaKBVal Val[t] $Complex$aKB$AHASIIICH3CO$[t] -
  krAHASIIIaKB $Complex$aKB$AHASIIICH3CO$Val$[t] -
  kriAHASIIIaKBVal $Complex$aKB$AHASIIICH3CO$Val$[t] -
  kcat$AHASIII$aKB residualRateAHASIIIValaKB $Complex$aKB$AHASIIICH3CO$Val$[t],
$Complex$aKB$KDC$′[t] == kfKDCaKB aKB[t] KDC[t] - kcat$KDC$aKB $Complex$aKB$KDC$[t]
  krKDCaKB $Complex$aKB$KDC$[t],
$Complex$aKG$TBNH2$′[t] == rkfTBaKG aKG[t] TBNH2[t] - rkcat$TB$aKG $Complex$aKG$TBN
  rkrTBaKG $Complex$aKG$TBNH2$[t],
$Complex$aKIC$TBNH2$′[t] == fkfTBaKIC aKIC[t] TBNH2[t] -
  fkcat$TB$aKIC $Complex$aKIC$TBNH2$[t] - fkrTBaKIC $Complex$aKIC$TBNH2$[t],
$Complex$AKII$Asp$ATP$′[t] == kfAKIIAsp AKII[t] Asp[t] ATP[t] -
  kcat$AKII$Asp $Complex$AKII$Asp$ATP$[t] - krAKIIAsp $Complex$AKII$Asp$ATP$[t],
$Complex$aKIV$IPMSacetyl$′[t] ==
 kfIPMSaKIV aKIV[t] IPMSacetyl[t] - kcat$IPMS$aKIV $Complex$aKIV$IPMSacetyl$[t] -
  krIPMSaKIV $Complex$aKIV$IPMSacetyl$[t] -
  kfiIPMSacetylLeu Leu[t] $Complex$aKIV$IPMSacetyl$[t] +
  kriIPMSacetylLeu $Complex$aKIV$IPMSacetyl$Leu$[t],
$Complex$aKIV$IPMSacetyl$Leu$′[t] ==
 kfiIPMSacetylLeu Leu[t] $Complex$aKIV$IPMSacetyl$[t] -
  kriIPMSacetylLeu $Complex$aKIV$IPMSacetyl$Leu$[t] -
  krIPMSaKIV $Complex$aKIV$IPMSacetyl$Leu$[t] +
  kfIPMSaKIV aKIV[t] $Complex$IPMSacetyl$Leu$[t],
$Complex$aKIV$TBNH2$′[t] == fkfTBaKIV aKIV[t] TBNH2[t] -
  fkcat$TB$aKIV $Complex$aKIV$TBNH2$[t] - fkrTBaKIV $Complex$aKIV$TBNH2$[t],
$Complex$aKIV$TCNH2$′[t] == fkfTCaKIV aKIV[t] TCNH2[t] -
  fkcat$TC$aKIV $Complex$aKIV$TCNH2$[t] - fkrTCaKIV $Complex$aKIV$TCNH2$[t],
$Complex$aKMV$TBNH2$′[t] == fkfTBaKMV aKMV[t] TBNH2[t] -
  fkcat$TB$aKMV $Complex$aKMV$TBNH2$[t] - fkrTBaKMV $Complex$aKMV$TBNH2$[t],
```

```
$Complex$Ala$TC$′[t] == fkfTCAla Ala[t] TC[t] – fkcat$TC$Ala $Complex$Ala$TC$[t] –
  fkrTCAla $Complex$Ala$TC$[t],
$Complex$ASD$ASA$NADP$Phosphate$′[t] ==
 kfASDASA ASA[t] ASD[t] NADP[t] Phosphate[t] –
  kcat$ASD$ASA $Complex$ASD$ASA$NADP$Phosphate$[t] –
  krASDASA $Complex$ASD$ASA$NADP$Phosphate$[t],
$Complex$ASD$AspP$NADPH$′[t] == kfASDAspP ASD[t] AspP[t] NADPH[t] –
  kcat$ASD$AspP $Complex$ASD$AspP$NADPH$[t] – krASDAspP $Complex$ASD$AspP$NADPH$[
$Complex$bIPM$IPMI$′[t] == kfIPMIbIPM bIPM[t] IPMI[t] –
  kcat$IPMI$bIPM $Complex$bIPM$IPMI$[t] – krIPMIbIPM $Complex$bIPM$IPMI$[t],
$Complex$exIle$LIVII$′[t] == kfLIVIIexIle exIle[t] LIVII[t] –
  kcat$LIVII$exIle $Complex$exIle$LIVII$[t] – krLIVIIexIle $Complex$exIle$LIVII$[
$Complex$exIle$LIVI$′[t] == kfLIVIexIle exIle[t] LIVI[t] –
  kcat$LIVI$exIle $Complex$exIle$LIVI$[t] – krLIVIexIle $Complex$exIle$LIVI$[t],
$Complex$exLeu$LIVII$′[t] == kfLIVIIexLeu exLeu[t] LIVII[t] –
  kcat$LIVII$exLeu $Complex$exLeu$LIVII$[t] – krLIVIIexLeu $Complex$exLeu$LIVII$[
$Complex$exLeu$LIVI$′[t] == kfLIVIexLeu exLeu[t] LIVI[t] –
  kcat$LIVI$exLeu $Complex$exLeu$LIVI$[t] – krLIVIexLeu $Complex$exLeu$LIVI$[t],
$Complex$exLeu$LS$′[t] == kfLSexLeu exLeu[t] LS[t] – kcat$LS$exLeu $Complex$exLeu$L
  krLSexLeu $Complex$exLeu$LS$[t],
$Complex$exVal$LIVII$′[t] == kfLIVIIexVal exVal[t] LIVII[t] –
  kcat$LIVII$exVal $Complex$exVal$LIVII$[t] – krLIVIIexVal $Complex$exVal$LIVII$[
$Complex$exVal$LIVI$′[t] == kfLIVIexVal exVal[t] LIVI[t] –
  kcat$LIVI$exVal $Complex$exVal$LIVI$[t] – krLIVIexVal $Complex$exVal$LIVI$[t],
$Complex$Glu$TB$′[t] == fkfTBGlu Glu[t] TB[t] – fkcat$TB$Glu $Complex$Glu$TB$[t] –
  fkrTBGlu $Complex$Glu$TB$[t],
$Complex$HDHII$ASA$NADPH$′[t] == kfHDHIIASA ASA[t] HDHII[t] NADPH[t] –
  kcat$HDHII$ASA $Complex$HDHII$ASA$NADPH$[t] – krHDHIIASA $Complex$HDHII$ASA$NAI
$Complex$HseP$TS$′[t] == kfTSHseP HseP[t] TS[t] – kcat$TS$HseP $Complex$HseP$TS$[t]
```

```
    krTSHseP $Complex$HseP$TS$[t],
  $Complex$HSK$Hse$ATP$′[t] == kfHSKHse ATP[t] Hse[t] HSK[t] -
    kcat$HSK$Hse $Complex$HSK$Hse$ATP$[t] - krHSKHse $Complex$HSK$Hse$ATP$[t],
  $Complex$HSK$Thr$′[t] == kfiHSKHseThr HSK[t] Thr[t] - kriHSKHseThr $Complex$HSK$Thr
  $Complex$Ile$TB$′[t] == rkfTBIle Ile[t] TB[t] - rkcat$TB$Ile $Complex$Ile$TB$[t] -
    rkrTBIle $Complex$Ile$TB$[t],
  $Complex$IPMDH$bIPM$NAD$′[t] == kfIPMDHbIPM bIPM[t] IPMDH[t] NAD[t] -
    kcat$IPMDH$bIPM $Complex$IPMDH$bIPM$NAD$[t] - krIPMDHbIPM $Complex$IPMDH$bIPM$N
  $Complex$IPMSacetyl$Leu$′[t] == kfiIPMSacetylLeu IPMSacetyl[t] Leu[t] +
    krIPMSaKIV $Complex$aKIV$IPMSacetyl$Leu$[t] -
    kriIPMSacetylLeu $Complex$IPMSacetyl$Leu$[t] -
    kfIPMSaKIV aKIV[t] $Complex$IPMSacetyl$Leu$[t],
  $Complex$IPMS$Leu$′[t] == kfiIPMSLeu IPMS[t] Leu[t] - kriIPMSLeu $Complex$IPMS$Leu$
  $Complex$IR$aAHB$NADPH$′[t] == kfIRaAHB aAHB[t] IR[t] NADPH[t] -
    kcat$IR$aAHB $Complex$IR$aAHB$NADPH$[t] - krIRaAHB $Complex$IR$aAHB$NADPH$[t],
  $Complex$IR$aAL$NADPH$′[t] == kfIRaAL aAL[t] IR[t] NADPH[t] -
    kcat$IR$aAL $Complex$IR$aAL$NADPH$[t] - krIRaAL $Complex$IR$aAL$NADPH$[t],
  $Complex$Leu$TB$′[t] == rkfTBLeu Leu[t] TB[t] - rkcat$TB$Leu $Complex$Leu$TB$[t] -
    rkrTBLeu $Complex$Leu$TB$[t],
  $Complex$Pyr$AHASICH3CO$′[t] == kfAHASIPyr2 AHASICH3CO[t] Pyr[t] -
    kcat$AHASI$Pyr2 $Complex$Pyr$AHASICH3CO$[t] - krAHASIPyr2 $Complex$Pyr$AHASICH3
    kfiAHASIPyr2Val Val[t] $Complex$Pyr$AHASICH3CO$[t] +
    kriAHASIPyr2Val $Complex$Pyr$AHASICH3CO$Val$[t],
  $Complex$Pyr$AHASICH3CO$Val$′[t] ==
   kfAHASIPyr2 Pyr[t] $Complex$AHASICH3CO$Val$[t] +
    kfiAHASIPyr2Val Val[t] $Complex$Pyr$AHASICH3CO$[t] -
    krAHASIPyr2 $Complex$Pyr$AHASICH3CO$Val$[t] -
    kriAHASIPyr2Val $Complex$Pyr$AHASICH3CO$Val$[t] -
    kcat$AHASI$Pyr2 residualRateAHASIValPyr2 $Complex$Pyr$AHASICH3CO$Val$[t],
```

```
$Complex$Pyr$AHASIICH3CO$′[t] == kfAHASIIPyr2 AHASIICH3CO[t] Pyr[t] -
    kcat$AHASII$Pyr2 $Complex$Pyr$AHASIICH3CO$[t] -
    krAHASIIPyr2 $Complex$Pyr$AHASIICH3CO$[t],
$Complex$Pyr$AHASIIICH3CO$′[t] ==
  kfAHASIIIPyr2 AHASIIICH3CO[t] Pyr[t] - kcat$AHASIII$Pyr2 $Complex$Pyr$AHASIIICH3
    krAHASIIIPyr2 $Complex$Pyr$AHASIIICH3CO$[t] -
    kfiAHASIIIPyr2Val Val[t] $Complex$Pyr$AHASIIICH3CO$[t] +
    kriAHASIIIPyr2Val $Complex$Pyr$AHASIIICH3CO$Val$[t],
$Complex$Pyr$AHASIIICH3CO$Val$′[t] ==
  kfAHASIIIPyr2 Pyr[t] $Complex$AHASIIICH3CO$Val$[t] +
    kfiAHASIIIPyr2Val Val[t] $Complex$Pyr$AHASIIICH3CO$[t] -
    krAHASIIIPyr2 $Complex$Pyr$AHASIIICH3CO$Val$[t] -
    kriAHASIIIPyr2Val $Complex$Pyr$AHASIIICH3CO$Val$[t] -
    kcat$AHASIII$Pyr2 residualRateAHASIIIValPyr2 $Complex$Pyr$AHASIIICH3CO$Val$[t
$Complex$Pyr$AHASIII$′[t] == kfAHASIIIPyr AHASIII[t] Pyr[t] -
    kcat$AHASIII$Pyr $Complex$Pyr$AHASIII$[t] - krAHASIIIPyr $Complex$Pyr$AHASIII$
    kfiAHASIIIPyrVal Val[t] $Complex$Pyr$AHASIII$[t] +
    kriAHASIIIPyrVal $Complex$Pyr$AHASIII$Val$[t],
$Complex$Pyr$AHASIII$Val$′[t] == kfAHASIIIPyr Pyr[t] $Complex$AHASIII$Val$[t] +
    kfiAHASIIIPyrVal Val[t] $Complex$Pyr$AHASIII$[t] -
    krAHASIIIPyr $Complex$Pyr$AHASIII$Val$[t] -
    kriAHASIIIPyrVal $Complex$Pyr$AHASIII$Val$[t] -
    kcat$AHASIII$Pyr residualRateAHASIIIValaKB $Complex$Pyr$AHASIII$Val$[t],
$Complex$Pyr$AHASII$′[t] == kfAHASIIPyr AHASII[t] Pyr[t] -
    kcat$AHASII$Pyr $Complex$Pyr$AHASII$[t] - krAHASIIPyr $Complex$Pyr$AHASII$[t],
$Complex$Pyr$AHASI$′[t] == kfAHASIPyr AHASI[t] Pyr[t] -
    kcat$AHASI$Pyr $Complex$Pyr$AHASI$[t] - krAHASIPyr $Complex$Pyr$AHASI$[t] -
    kfiAHASIPyrVal Val[t] $Complex$Pyr$AHASI$[t] + kriAHASIPyrVal $Complex$Pyr$AHAS
$Complex$Pyr$AHASI$Val$′[t] == kfAHASIPyr Pyr[t] $Complex$AHASI$Val$[t] +
```

```
    kfiAHASIPyrVal Val[t] $Complex$Pyr$AHASI$[t] - krAHASIPyr $Complex$Pyr$AHASI$Va
    kriAHASIPyrVal $Complex$Pyr$AHASI$Val$[t] -
    kcat$AHASI$Pyr residualRateAHASIValaKB $Complex$Pyr$AHASI$Val$[t],
  $Complex$Pyr$TCNH2$′[t] == rkfTCPyr Pyr[t] TCNH2[t] - rkcat$TC$Pyr $Complex$Pyr$TCN
    rkrTCPyr $Complex$Pyr$TCNH2$[t],
  $Complex$Val$TB$′[t] == rkfTBVal TB[t] Val[t] - rkcat$TB$Val $Complex$Val$TB$[t] -
    rkrTBVal $Complex$Val$TB$[t],
  $Complex$Val$TC$′[t] == rkfTCVal TC[t] Val[t] - rkcat$TC$Val $Complex$Val$TC$[t] -
    rkrTCVal $Complex$Val$TC$[t]},
 {aAHB, aAL, acetylCoA, aDHIV, aDMV, ADP, AHASI, AHASICH3CO, AHASII, AHASIICH3CO,
  AHASIII, AHASIIICH3CO, aIPM, aKB, aKG, AKI, aKIC, AKII, AKIII, aKIV, aKMV, Ala,
  ASA, ASD, Asp, AspP, ATP, bIPM, CO2, CoA, DAD, exIle, exLeu, exVal, Glu, glutarylCo
  HDHI, HDHII, Hse, HseP, HSK, Ile, IPMDH, IPMI, IPMS, IPMSacetyl, IR, KDC, Leu,
  LIVI, LIVII, LS, Lys, NAD, NADH, NADP, NADPH, NH3, pantothenate, Phosphate,
  propionylCoA, protein, Pyr, TB, TBNH2, TC, TCNH2, TDA, Thr, TS, Val,
  $Complex$acetylCoA$IPMS$, $Complex$aDHIV$DAD$, $Complex$aDMV$DAD$,
  $Complex$AHASICH3CO$Val$, $Complex$AHASIIICH3CO$Val$, $Complex$AHASIII$Val$,
  $Complex$AHASI$Val$, $Complex$aIPM$IPMI$, $Complex$aKB$AHASICH3CO$,
  $Complex$aKB$AHASICH3CO$Val$, $Complex$aKB$AHASIICH3CO$, $Complex$aKB$AHASIIICH
  $Complex$aKB$AHASIIICH3CO$Val$, $Complex$aKB$KDC$, $Complex$aKG$TBNH2$,
  $Complex$aKIC$TBNH2$, $Complex$AKII$Asp$ATP$, $Complex$aKIV$IPMSacetyl$,
  $Complex$aKIV$IPMSacetyl$Leu$, $Complex$aKIV$TBNH2$, $Complex$aKIV$TCNH2$,
  $Complex$aKMV$TBNH2$, $Complex$Ala$TC$, $Complex$ASD$ASA$NADP$Phosphate$,
  $Complex$ASD$AspP$NADPH$, $Complex$bIPM$IPMI$, $Complex$exIle$LIVII$,
  $Complex$exIle$LIVI$, $Complex$exLeu$LIVII$, $Complex$exLeu$LIVI$, $Complex$exLe
  $Complex$exVal$LIVII$, $Complex$exVal$LIVI$, $Complex$Glu$TB$, $Complex$HDHII$AS
  $Complex$HseP$TS$, $Complex$HSK$Hse$ATP$, $Complex$HSK$Thr$, $Complex$Ile$TB$,
  $Complex$IPMDH$bIPM$NAD$, $Complex$IPMSacetyl$Leu$, $Complex$IPMS$Leu$,
  $Complex$IR$aAHB$NADPH$, $Complex$IR$aAL$NADPH$, $Complex$Leu$TB$,
```

```
$Complex$Pyr$AHASICH3CO$, $Complex$Pyr$AHASICH3CO$Val$, $Complex$Pyr$AHASIICH3C
$Complex$Pyr$AHASIIICH3CO$, $Complex$Pyr$AHASIIICH3CO$Val$, $Complex$Pyr$AHASII
$Complex$Pyr$AHASIII$Val$, $Complex$Pyr$AHASII$, $Complex$Pyr$AHASI$,
$Complex$Pyr$AHASI$Val$, $Complex$Pyr$TCNH2$, $Complex$Val$TB$, $Complex$Val$TC$
```

```
(*
 Inputs for Values of Km , Ki and kcat for each Enzyme.
  Lambda Approximation Functions:Kf[],Kf2S[] for kf,
                                       Kr[] for kr
  Omega Approximation Functions: Kfi[] for kfi,
                                       Kri[] for kri.
 *)

Lambda = 100;
Omega = 1;
myKs = {

   cASA = 1;
   LASA = 1;
   nASA = 4;
   KmHDHIASA = 80;
   kcat$AKI$HDHI = 10000;

   cAsp = 1;
   LAsp = 1;
   nAsp = 4;
   KmAKIAsp = 220;
   kcat$HDHI$AKI = 10000;
```

```
cAKI = 0.0001;
LAKI = 10000;
nAKI = 4;
KmAKIAsp = 220;
KiAKIAspThr = 135;
kcat$AKI$Asp = 100;

cAKIII = 0.00005;
LAKIII = 1000;
nAKIII = 2;
KmAKIIIAsp = 30;
KiAKIIIAspLys = 15;
kcat$AKIII$Asp = 20;

cHDHI = 0.008;
LHDHI = 3000;
nHDHI = 4;
KmHDHIASA = 80;
KiHDHIASAThr = 120;
kcat$HDHI$ASA = 800;

kfHDHIAKI = 1000;
kfAKIHDHI = 1000;

KmAKIIAsp = 100; kcat$AKII$Asp = 100;
kfAKIIAsp → Kf[KmAKIIAsp, kcat$AKII$Asp, Lambda],
krAKIIAsp → Kr[kcat$AKII$Asp, Lambda],

kmASDAspP = 22; kcat$ASD$AspP = 40;
```

```
kfASDAspP → Kf[kmASDAspP, kcat$ASD$AspP, Lambda],
krASDAspP → Kr[kcat$ASD$AspP, Lambda],

KmASDASA = 110; KmASDNADP = 140; KmASDPhosphate = 10200; kcat$ASD$ASA = 80;
kfASDASA → Kf3S[KmASDASA, KmASDNADP, KmASDPhosphate, kcat$ASD$ASA, Lambda],
krASDASA → Kr[kcat$ASD$ASA, Lambda],

KmHDHIIASA = 100; kcat$HDHII$ASA = 100;
kfHDHIIASA → Kf[KmHDHIIASA, kcat$HDHII$ASA, Lambda],
krHDHIIASA → Kr[kcat$HDHII$ASA, Lambda],

KmHSKHse = 110; kcat$HSK$Hse = 60;
kfHSKHse → Kf[KmHSKHse, kcat$HSK$Hse, Lambda],
krHSKHse → Kr[kcat$HSK$Hse, Lambda],

KiHSKHseThr = 1000;
kfiHSKHseThr → Kfi[KmHSKHse, kcat$HSK$Hse, Lambda, Omega],
kriHSKHseThr → Kri[KmHSKHse, kcat$HSK$Hse, Lambda, Omega, KiHSKHseThr],

kfASA = 0.05;
kfHse = 0.05;

KmTSHseP = 310; kcat$TS$HseP = 60;
kfTSHseP → Kf[KmTSHseP, kcat$TS$HseP, Lambda],
krTSHseP → Kr[kcat$TS$HseP, Lambda],

nTDA → 4,
cTDA → 0.013,
L0TDA → 1.05,
```

```
kcat$TDA → 6000,
KmThr → 2700,
KaVal → 550,
KiIle → 15,                    (* TDA feedback resistant mutant, KiIle=100000 *)

KmKDCaKB = 1000; kcat$KDC$aKB = 3000;
kfKDCaKB → Kf[KmKDCaKB, kcat$KDC$aKB, Lambda],
krKDCaKB → Kr[kcat$KDC$aKB, Lambda],

KmAHASIPyr = 10; kcat$AHASI$Pyr = 7000;
kfAHASIPyr → Kf[KmAHASIPyr, kcat$AHASI$Pyr, Lambda],
krAHASIPyr → Kr[kcat$AHASI$Pyr, Lambda],

KmAHASIaKB = 5000; kcat$AHASI$aKB = 7000;
kfAHASIaKB → Kf[KmAHASIaKB, kcat$AHASI$aKB, Lambda],
krAHASIaKB → Kr[kcat$AHASI$aKB, Lambda],
residualRateAHASIValaKB → 0,

KiAHASIVal = 200;
kfiAHASIPyrVal → Kfi[KmAHASIPyr, kcat$AHASI$Pyr, Lambda, Omega],
kriAHASIPyrVal → Kri[KmAHASIPyr, kcat$AHASI$Pyr, Lambda, Omega, KiAHASIVal],
kfiAHASIaKBVal → Kfi[KmAHASIaKB, kcat$AHASI$aKB, Lambda, Omega],
kriAHASIaKBVal → Kri[KmAHASIaKB, kcat$AHASI$aKB, Lambda, Omega, KiAHASIVal],

KmAHASIIPyr = 10; kcat$AHASII$Pyr = 7000;
kfAHASIIPyr → Kf[KmAHASIIPyr, kcat$AHASII$Pyr, Lambda],
krAHASIIPyr → Kr[kcat$AHASII$Pyr, Lambda],

KmAHASIIaKB = 150; kcat$AHASII$aKB = 7000;
```

```
kfAHASIIaKB → Kf[KmAHASIIaKB, kcat$AHASII$aKB, Lambda],
krAHASIIaKB → Kr[kcat$AHASII$aKB, Lambda],

KmAHASIIIPyr = 10; kcat$AHASIII$Pyr = 7000;
kfAHASIIIPyr → Kf[KmAHASIIIPyr, kcat$AHASIII$Pyr, Lambda],
krAHASIIIPyr → Kr[kcat$AHASIII$Pyr, Lambda],

KmAHASIIIaKB = 150; kcat$AHASIII$aKB = 7000;
kfAHASIIIaKB → Kf[KmAHASIIIaKB, kcat$AHASIII$aKB, Lambda],
krAHASIIIaKB → Kr[kcat$AHASIII$aKB, Lambda],
residualRateAHASIIIValaKB → 0.15,

KiAHASIIIVal = 20;
kfiAHASIIIPyrVal → Kfi[KmAHASIIIPyr, kcat$AHASIII$Pyr, Lambda, Omega],
kriAHASIIIPyrVal → Kri[KmAHASIIIPyr, kcat$AHASIII$Pyr, Lambda, Omega, KiAHASIIIVa
kfiAHASIIIaKBVal → Kfi[KmAHASIIIaKB, kcat$AHASIII$aKB, Lambda, Omega],
kriAHASIIIaKBVal → Kri[KmAHASIIIaKB, kcat$AHASIII$aKB, Lambda, Omega, KiAHASIIIVa

KmIRaAHB = 780; KmIRNADPH = 15; kcat$IR$aAHB = 4700;
kfIRaAHB → Kf2S[KmIRaAHB, KmIRNADPH, kcat$IR$aAHB, Lambda],
krIRaAHB → Kr[kcat$IR$aAHB, Lambda],

KmDADaDMV = 750; kcat$DAD$aDMV = 24000;
kfDADaDMV → Kf[KmDADaDMV, kcat$DAD$aDMV, Lambda],
krDADaDMV → Kr[kcat$DAD$aDMV, Lambda],

KmTBGlu = 1000; fkcat$TB$Glu = 2000;
fkfTBGlu → Kf[KmTBGlu, fkcat$TB$Glu, Lambda],
fkrTBGlu → Kr[fkcat$TB$Glu, Lambda],
```

```
KmTBaKMV = 200; fkcat$TB$aKMV = 1500;
fkfTBaKMV → Kf[KmTBaKMV, fkcat$TB$aKMV, Lambda],
fkrTBaKMV → Kr[fkcat$TB$aKMV, Lambda],

KmTBIle = 600; rkcat$TB$Ile = 3000;
rkfTBIle → Kf[KmTBIle, rkcat$TB$Ile, Lambda],
rkrTBIle → Kr[rkcat$TB$Ile, Lambda],

KmTBaKG = 2500; rkcat$TB$aKG = 2100;
rkfTBaKG → Kf[KmTBaKG, rkcat$TB$aKG, Lambda],
rkrTBaKG → Kr[rkcat$TB$aKG, Lambda],

KmLIVIexIle = 7; kcat$LIVI$exIle = 200;
kfLIVIexIle → Kf[KmLIVIexIle, kcat$LIVI$exIle, Lambda],
krLIVIexIle → Kr[kcat$LIVI$exIle, Lambda],

KmLIVIIexIle = 1; kcat$LIVII$exIle = 1;
kfLIVIIexIle → Kf[KmLIVIIexIle, kcat$LIVII$exIle, Lambda],
krLIVIIexIle → Kr[kcat$LIVII$exIle, Lambda],

kcat$aKMV → 5,
kcat$Ile → 0.2,

KmAHASIPyr = 10; kcat$AHASI$Pyr = 7000;
kfAHASIPyr → Kf[KmAHASIPyr, kcat$AHASI$Pyr, Lambda],
krAHASIPyr → Kr[kcat$AHASI$Pyr, Lambda],

KmAHASIPyr2 = 1000; kcat$AHASI$Pyr2 = 7000;
```

```
kfAHASIPyr2 → Kf[KmAHASIPyr2, kcat$AHASI$Pyr2, Lambda],
krAHASIPyr2 → Kr[kcat$AHASI$Pyr2, Lambda],
residualRateAHASIValPyr2 → 0,

KiAHASIVal = 200;
kfiAHASIPyrVal → Kfi[KmAHASIPyr, kcat$AHASI$Pyr, Lambda, Omega],
kriAHASIPyrVal → Kri[KmAHASIPyr, kcat$AHASI$Pyr, Lambda, Omega, KiAHASIVal],
kfiAHASIPyr2Val → Kfi[KmAHASIPyr2, kcat$AHASI$Pyr2, Lambda, Omega],
kriAHASIPyr2Val → Kri[KmAHASIPyr2, kcat$AHASI$Pyr2, Lambda, Omega, KiAHASIVal],

KmAHASIIPyr = 10; kcat$AHASII$Pyr = 7000;
kfAHASIIPyr → Kf[KmAHASIIPyr, kcat$AHASII$Pyr, Lambda],
krAHASIIPyr → Kr[kcat$AHASII$Pyr, Lambda],

KmAHASIIPyr2 = 10000; kcat$AHASII$Pyr2 = 7000;
kfAHASIIPyr2 → Kf[KmAHASIIPyr2, kcat$AHASII$Pyr2, Lambda],
krAHASIIPyr2 → Kr[kcat$AHASII$Pyr2, Lambda],

KmAHASIIIPyr = 10; kcat$AHASIII$Pyr = 7000;
kfAHASIIIPyr → Kf[KmAHASIIIPyr, kcat$AHASIII$Pyr, Lambda],
krAHASIIIPyr → Kr[kcat$AHASIII$Pyr, Lambda],

KmAHASIIIPyr2 = 7000; kcat$AHASIII$Pyr2 = 7000;
kfAHASIIIPyr2 → Kf[KmAHASIIIPyr2, kcat$AHASIII$Pyr2, Lambda],
krAHASIIIPyr2 → Kr[kcat$AHASIII$Pyr2, Lambda],
residualRateAHASIIIValPyr2 → 0.15,

KiAHASIIIVal = 20;
kfiAHASIIIPyrVal → Kfi[KmAHASIIIPyr, kcat$AHASIII$Pyr, Lambda, Omega],
```

```
kriAHASIIIPyrVal → Kri[KmAHASIIIPyr, kcat$AHASIII$Pyr, Lambda, Omega, KiAHASIIIVa
kfiAHASIIIPyr2Val → Kfi[KmAHASIIIPyr2, kcat$AHASIII$Pyr2, Lambda, Omega],
kriAHASIIIPyr2Val → Kri[KmAHASIIIPyr2, kcat$AHASIII$Pyr2, Lambda, Omega, KiAHASII

KmIRaAL = 290; KmIRNADPH = 15; kcat$IR$aAL = 1100;
kfIRaAL → Kf2S[KmIRaAL, KmIRNADPH, kcat$IR$aAL, Lambda],
krIRaAL → Kr[kcat$IR$aAL, Lambda],

KmDADaDHIV = 2800; kcat$DAD$aDHIV = 24000;
kfDADaDHIV → Kf[KmDADaDHIV, kcat$DAD$aDHIV, Lambda],
krDADaDHIV → Kr[kcat$DAD$aDHIV, Lambda],

KmTBGlu = 1000; fkcat$TB$Glu = 2000;
fkfTBGlu → Kf[KmTBGlu, fkcat$TB$Glu, Lambda],
fkrTBGlu → Kr[fkcat$TB$Glu, Lambda],

KmTBaKIV = 300; fkcat$TB$aKIV = 930;
fkfTBaKIV → Kf[KmTBaKIV, fkcat$TB$aKIV, Lambda],
fkrTBaKIV → Kr[fkcat$TB$aKIV, Lambda],

KmTBVal = 2700; rkcat$TB$Val = 2000;
rkfTBVal → Kf[KmTBVal, rkcat$TB$Val, Lambda],
rkrTBVal → Kr[rkcat$TB$Val, Lambda],

KmTBaKG = 2500; rkcat$TB$aKG = 2100;
rkfTBaKG → Kf[KmTBaKG, rkcat$TB$aKG, Lambda],
rkrTBaKG → Kr[rkcat$TB$aKG, Lambda],

KmTCAla = 100; fkcat$TC$Ala = 2000;
```

```
fkfTCAla → Kf[KmTCAla, fkcat$TC$Ala, Lambda],
fkrTCAla → Kr[fkcat$TC$Ala, Lambda],

KmTCaKIV = 100; fkcat$TC$aKIV = 1500;
fkfTCaKIV → Kf[KmTCaKIV, fkcat$TC$aKIV, Lambda],
fkrTCaKIV → Kr[fkcat$TC$aKIV, Lambda],

KmTCVal = 3000;  rkcat$TC$Val = 3000;
rkfTCVal → Kf[KmTCVal, rkcat$TC$Val, Lambda],
rkrTCVal → Kr[rkcat$TC$Val, Lambda],

KmTCPyr = 2000;  rkcat$TC$Pyr = 3000;
rkfTCPyr → Kf[KmTCPyr, rkcat$TC$Pyr, Lambda],
rkrTCPyr → Kr[rkcat$TC$Pyr, Lambda],

KmLIVIexVal = 2; kcat$LIVI$exVal = 500;
kfLIVIexVal → Kf[KmLIVIexVal, kcat$LIVI$exVal, Lambda],
krLIVIexVal → Kr[kcat$LIVI$exVal, Lambda],

KmLIVIIexVal = 1; kcat$LIVII$exVal = 1;
kfLIVIIexVal → Kf[KmLIVIIexVal, kcat$LIVII$exVal, Lambda],
krLIVIIexVal → Kr[kcat$LIVII$exVal, Lambda],

kcat$aKIV → 70,
kcat$Val → 0.2,

KmIPMSacetylCoA = 200 ; kcat$IPMS$acetylCoA = 1000;
kfIPMSacetylCoA → Kf[KmIPMSacetylCoA, kcat$IPMS$acetylCoA, Lambda],
krIPMSacetylCoA → Kr[kcat$IPMS$acetylCoA, Lambda],
```

```
KmIPMSaKIV = 60; kcat$IPMS$aKIV = 1000;
kfIPMSaKIV → Kf[KmIPMSaKIV, kcat$IPMS$aKIV, Lambda],
krIPMSaKIV → Kr[kcat$IPMS$aKIV, Lambda],

KiIPMSLeu = 200;
kfiIPMSLeu → Kfi[KmIPMSacetylCoA, kcat$IPMS$acetylCoA, Lambda, Omega],
kriIPMSLeu → Kri[KmIPMSacetylCoA, kcat$IPMS$acetylCoA, Lambda, Omega, KiIPMSLeu],

KiIPMSacetylLeu = 500;
kfiIPMSacetylLeu → Kfi[KmIPMSaKIV, kcat$IPMS$aKIV, Lambda, Omega],
kriIPMSacetylLeu → Kri[KmIPMSaKIV, kcat$IPMS$aKIV, Lambda, Omega, KiIPMSacetylLeu

KmIPMIaIPM = 100; kcat$IPMI$aIPM = 1000;
kfIPMIaIPM → Kf[KmIPMIaIPM, kcat$IPMI$aIPM, Lambda],
krIPMIaIPM → Kr[kcat$IPMI$aIPM, Lambda],

KmIPMIbIPM = 100; kcat$IPMI$bIPM = 1000;
kfIPMIbIPM → Kf[KmIPMIbIPM, kcat$IPMI$bIPM, Lambda],
krIPMIbIPM → Kr[kcat$IPMI$bIPM, Lambda],

KmIPMDHbIPM = 105; KmIPMDHNAD = 320; kcat$IPMDH$bIPM = 4000;
kfIPMDHbIPM → Kf2S[KmIPMDHbIPM, KmIPMDHNAD, kcat$IPMDH$bIPM, Lambda],
krIPMDHbIPM → Kr[kcat$IPMDH$bIPM, Lambda],

KmTBGlu = 1000; fkcat$TB$Glu = 2000;
fkfTBGlu → Kf[KmTBGlu, fkcat$TB$Glu, Lambda],
fkrTBGlu → Kr[fkcat$TB$Glu, Lambda],
```

```
  KmTBaKIC = 200; fkcat$TB$aKIC = 3600;
  fkfTBaKIC → Kf[KmTBaKIC, fkcat$TB$aKIC, Lambda],
  fkrTBaKIC → Kr[fkcat$TB$aKIC, Lambda],

  KmTBLeu = 4400; rkcat$TB$Leu = 2800;
  rkfTBLeu → Kf[KmTBLeu, rkcat$TB$Leu, Lambda],
  rkrTBLeu → Kr[rkcat$TB$Leu, Lambda],

  KmTBaKG = 2500; rkcat$TB$aKG = 2100;
  rkfTBaKG → Kf[KmTBaKG, rkcat$TB$aKG, Lambda],
  rkrTBaKG → Kr[rkcat$TB$aKG, Lambda],

  KmLIVIexLeu = 4; kcat$LIVI$exLeu = 100;
  kfLIVIexLeu → Kf[KmLIVIexLeu, kcat$LIVI$exLeu, Lambda],
  krLIVIexLeu → Kr[kcat$LIVI$exLeu, Lambda],

  KmLIVIIexLeu = 1; kcat$LIVII$exLeu = 1;
  kfLIVIIexLeu → Kf[KmLIVIIexLeu, kcat$LIVII$exLeu, Lambda],
  krLIVIIexLeu → Kr[kcat$LIVII$exLeu, Lambda],

  KmLSexLeu = 0.5; kcat$LS$exLeu = 100;
  kfLSexLeu → Kf[KmLSexLeu, kcat$LS$exLeu, Lambda],
  krLSexLeu → Kr[kcat$LS$exLeu, Lambda],

  kcat$aKIC → 25,
  kcat$Leu → 0.01
 };
```

```
(*
  Inputs for Values of Substrate and Enzyme Concentrations (μM)
  { note: NADP[0]=0 in yang's pathway}
*)

myICs = {
   AKI[0] == 8.1,
   AKII[0] == 0,
   AKIII[0] == 9.5,
   ASD[0] == 6.2,
   HDHI[0] == 8.1,
   HDHII[0] == 0,
   HSK[0] == 5.6,
   TS[0] == 8.3,

   Asp[0] == 3600,
   AspP[0] == 0,
   ASA[0] == 0,
   Hse[0] == 0,
   HseP[0] == 0,
   Lys[0] == 1000,
   Thr[0] == 0,

   ATP[0] == 1000,
   ADP[0] == 0,
   NADP[0] == 1000,
   NADPH[0] == 1000,
   Phosphate[0] == 1000,
```

```
$Complex$AKII$Asp$ATP$[0] == 0,
$Complex$ASD$ASA$NADP$Phosphate$[0] == 0,
$Complex$ASD$AspP$NADPH$[0] == 0,
$Complex$HDHII$ASA$NADPH$[0] == 0,
$Complex$HseP$TS$[0] == 0,
$Complex$HSK$Hse$ATP$[0] == 0,
$Complex$HSK$Thr$[0] == 0,

Pyr[0] == 1000,
Leu[0] == 0,
Ala[0] == 2000,
Glu[0] == 2000,
Ile[0] == 0,
Val[0] == 0,
aKB[0] == 0,
NH3[0] == 0,
aAHB[0] == 0,
aDMV[0] == 0,
aKMV[0] == 0,
aKG[0] == 0,
aAL[0] == 0,
aDHIV[0] == 0,
aKIV[0] == 0,
aIPM[0] == 0,
bIPM[0] == 0,
aKIC[0] == 0,

propionylCoA[0] == 0,
glutarylCoA[0] == 0,
```

```
pantothenate[0] == 0,
acetylCoA[0] == 1000,
CoA[0] == 0,
NAD[0] == 1000,
NADH[0] == 0,
CO2[0] == 0,
protein[0] == 0,

TDA[0] == 3,
KDC[0] == 2,

AHASI[0] == 10,
AHASICH3CO[0] == 0,
AHASII[0] == 0,           (* E. coli K12 has no active AHASII *)
AHASIICH3CO[0] == 0,
AHASIII[0] == 2,
AHASIIICH3CO[0] == 0,

IR[0] == 13.5,
DAD[0] == 7,

TB[0] == 2.5,
TBNH2[0] == 0,
TC[0] == 2,
TCNH2[0] == 0,

IPMS[0] == 5,
IPMSacetyl[0] == 0,
IPMI[0] == 6,
```

```
IPMDH[0] == 5,

LIVI[0] == 10,
LIVII[0] == 0,
LS[0] == 8,
                        (* extracellular amino acid treatment *)
exVal[0] == 0,          (* 1000 for valine growth inhibition *)
exIle[0] == 0,          (* 500 for isoleucine rescue *)
exLeu[0] == 0,


$Complex$aKB$KDC$[0] == 0, $Complex$Pyr$AHASI$[0] == 0,
$Complex$AHASI$Val$[0] == 0, $Complex$Pyr$AHASI$Val$[0] == 0,
$Complex$AHASICH3CO$Val$[0] == 0, $Complex$aKB$AHASICH3CO$[0] == 0,
$Complex$aKB$AHASICH3CO$Val$[0] == 0, $Complex$Pyr$AHASICH3CO$[0] == 0,
$Complex$Pyr$AHASICH3CO$Val$[0] == 0, $Complex$Pyr$AHASII$[0] == 0,
$Complex$aKB$AHASIICH3CO$[0] == 0, $Complex$Pyr$AHASIICH3CO$[0] == 0,
$Complex$Pyr$AHASIII$[0] == 0, $Complex$AHASIII$Val$[0] == 0,
$Complex$Pyr$AHASIII$Val$[0] == 0, $Complex$AHASIIICH3CO$Val$[0] == 0,
$Complex$aKB$AHASIIICH3CO$[0] == 0, $Complex$aKB$AHASIIICH3CO$Val$[0] == 0,
$Complex$Pyr$AHASIIICH3CO$[0] == 0, $Complex$Pyr$AHASIIICH3CO$Val$[0] == 0,
$Complex$IR$aAHB$NADPH$[0] == 0, $Complex$IR$aAL$NADPH$[0] == 0,
$Complex$aDHIV$DAD$[0] == 0, $Complex$aDMV$DAD$[0] == 0,
$Complex$Glu$TB$[0] == 0, $Complex$aKMV$TBNH2$[0] == 0,
$Complex$aKIV$TBNH2$[0] == 0, $Complex$Ile$TB$[0] == 0,
$Complex$Val$TB$[0] == 0, $Complex$aKG$TBNH2$[0] == 0,
$Complex$Ala$TC$[0] == 0, $Complex$aKIV$TCNH2$[0] == 0,
$Complex$Val$TC$[0] == 0, $Complex$Pyr$TCNH2$[0] == 0,
$Complex$exIle$LIVI$[0] == 0, $Complex$exIle$LIVII$[0] == 0,
```

```
   $Complex$exVal$LIVI$[0] == 0, $Complex$exVal$LIVII$[0] == 0,
   $Complex$acetylCoA$IPMS$[0] == 0, $Complex$aKIV$IPMSacetyl$[0] == 0,
   $Complex$IPMS$Leu$[0] == 0, $Complex$IPMSacetyl$Leu$[0] == 0,
   $Complex$aKIV$IPMSacetyl$Leu$[0] == 0, $Complex$aIPM$IPMI$[0] == 0,
   $Complex$bIPM$IPMI$[0] == 0, $Complex$IPMDH$bIPM$NAD$[0] == 0,
   $Complex$aKIC$TBNH2$[0] == 0, $Complex$Leu$TB$[0] == 0,
   $Complex$exLeu$LIVI$[0] == 0, $Complex$exLeu$LIVII$[0] == 0,
   $Complex$exLeu$LS$[0] == 0};
```

```
(*
 Call the Mathematica NDSolve Function to Solve the ODEs with Given
  Values of Kinetic Parameters and Substrate and Enzyme Concentrations
  Listed Above.
 *)

tmax = 100;  (* minutes *)
mySolution = NDSolve[Join[myODEs /. myKs, myICs], myVars, {t, 0, tmax}, AccuracyGoal →
   PrecisionGoal → 3, MaxSteps → 30000];
```

```
(* List of Substrates, Intermediates and Products for Graphic Outputs
  Displayed Below *)

metabolites = {
   Asp, AspP, ASA, Hse, HseP, HSK,
   Thr, Pyr, aKB, aAHB, aAL, NADPH, aDMV, aDHIV, aKMV, aKIV, acetylCoA, aIPM, bIPM,
   NAD, aKIC, Glu, Ala, aKG, Ile, Val, Leu
  };
```

```
TBTCdistribution = {
   TB, TBNH2, $Complex$Glu$TB$, $Complex$aKMV$TBNH2$, $Complex$aKIV$TBNH2$,
   $Complex$aKIC$TBNH2$,
   $Complex$Ile$TB$, $Complex$Val$TB$, $Complex$Leu$TB$, $Complex$aKG$TBNH2$,
   TC, TCNH2, $Complex$Ala$TC$, $Complex$aKIV$TCNH2$,
   $Complex$Val$TC$, $Complex$Pyr$TCNH2$};
```

```
(* Define some plotting functions *)
FormalPlot[varName_, displayTime_, YplotRange_] :=
  Plot[varName[t] /. mySolution, {t, 0, displayTime}, PlotRange → YplotRange,
   Background → GrayLevel[1],
   TextStyle → {FontWeight → "Bold", FontSize → 14, FontFamily →  "Times New Roman"},
   PlotStyle → {Thickness[0.01]}, AxesStyle → {Thickness[0.008]}];
DashPlot[varName_, displayTime_, YplotRange_] :=
  Plot[varName[t] /. mySolution, {t, 0, displayTime}, PlotRange → YplotRange,
   Background → GrayLevel[1],
   TextStyle → {FontWeight → "Bold", FontSize → 12, FontFamily →  "Times New Roman"},
   PlotStyle → {Thickness[0.008], Dashing[{0.02, 0.025}]}, AxesStyle → {Thickness[0.
DotPlot[varName_, displayTime_, YplotRange_] :=
  Plot[varName[t] /. mySolution, {t, 0, displayTime}, PlotRange → YplotRange,
   Background → GrayLevel[1],
   TextStyle → {FontWeight → "Bold", FontSize → 12, FontFamily →  "Times New Roman"},
   PlotStyle → {Thickness[0.008], Dashing[{0.001, 0.001, 0.001, 0.015}]},
   AxesStyle → {Thickness[0.0006]}];
```

```
(*
  Display Results of Simulation of Branched Chain Amino Acid
  Simulation: Rates of Production of Metabolic Intermediates and End-
    products.

      X axis is Time (min), and Y axis is Concentration (μM)
 *)

displayTime = 100;
Show[
  GraphicsArray[
   Partition[
    Map[Plot[#[t] /. mySolution, {t, 0, displayTime}, PlotLabel → #, PlotRange → All,
       DisplayFunction → Identity] &, metabolites], 3]
  ]
 ];
```

```
FormalPlot[aKB, 100, All];
```

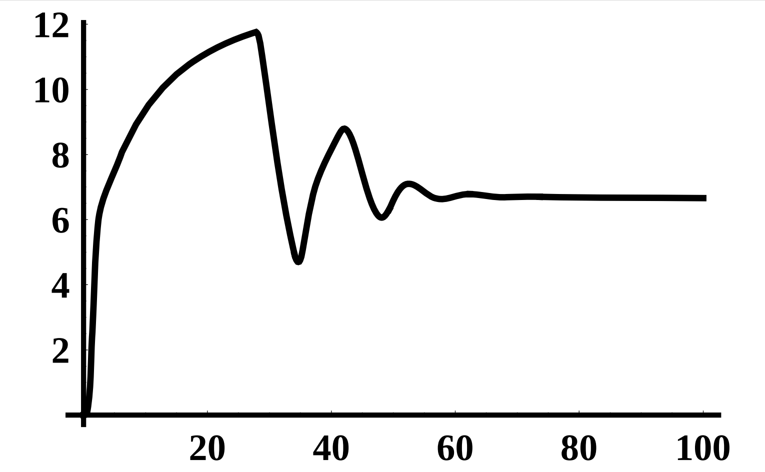


```
FormalPlot[aAHB, 100, All];
```

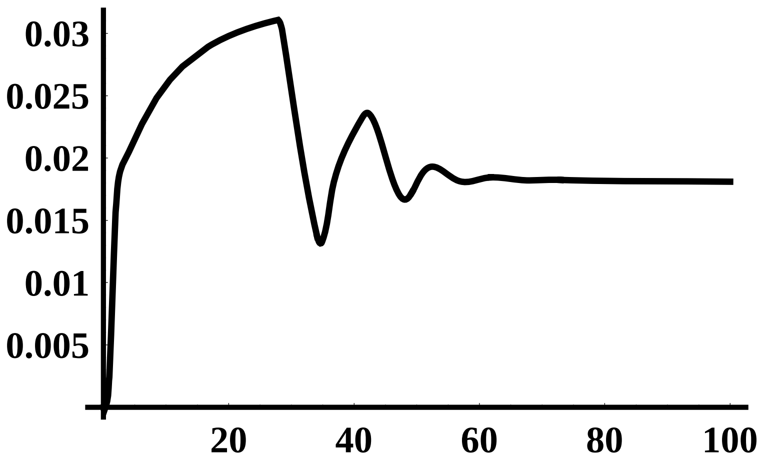

```
FormalPlot[aDMV, 100, All];
```

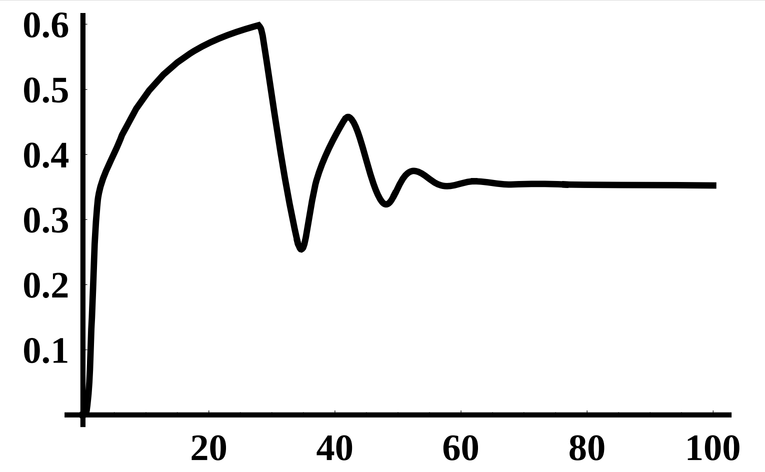


```
FormalPlot[aKMV, 100, All];
```

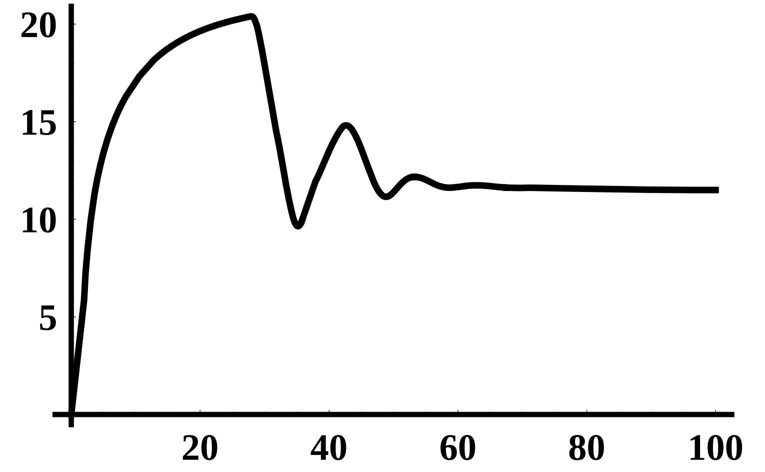

```
FormalPlot[Ile, 100, All];
```

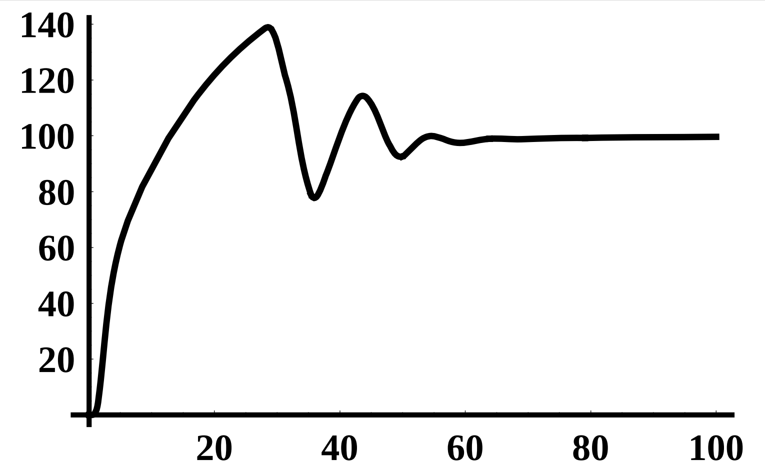


```
FormalPlot[Pyr, 100, All];
```

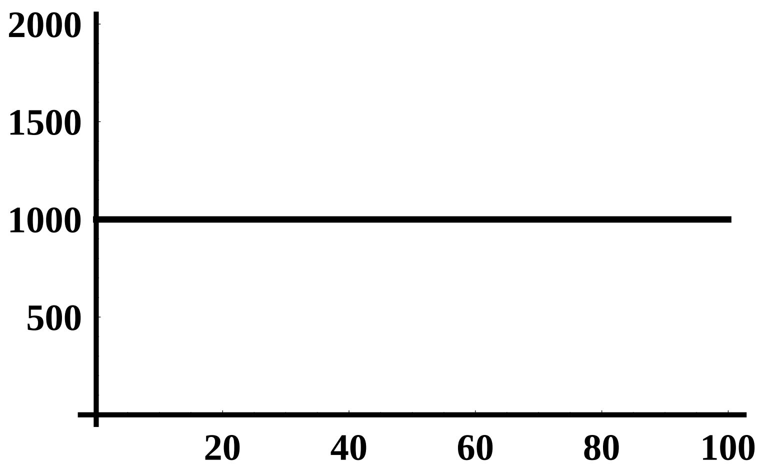

```
FormalPlot[aAL, 100, {0, 10}];
```

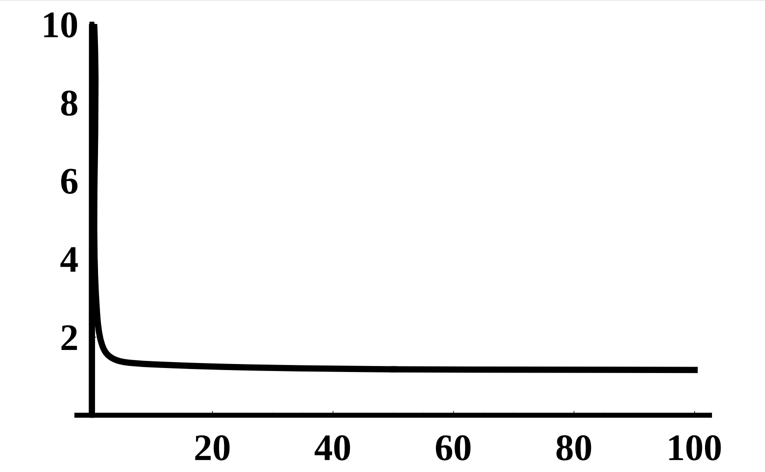


```
FormalPlot[aDHIV, 100, All];
```

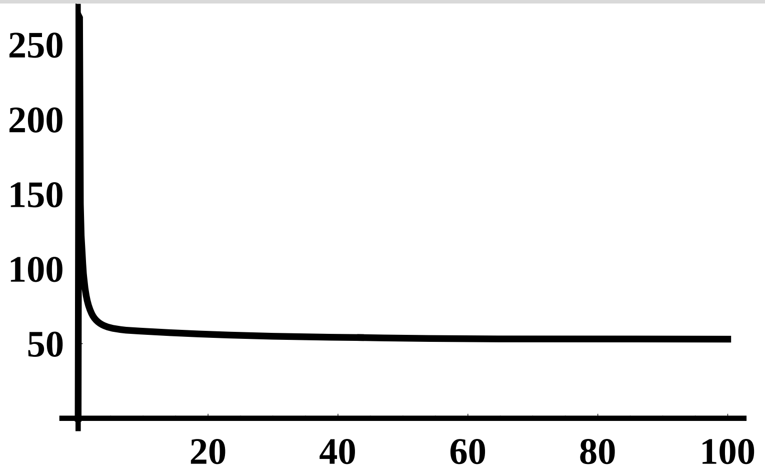

```
FormalPlot[aKIV, 100, All];
```

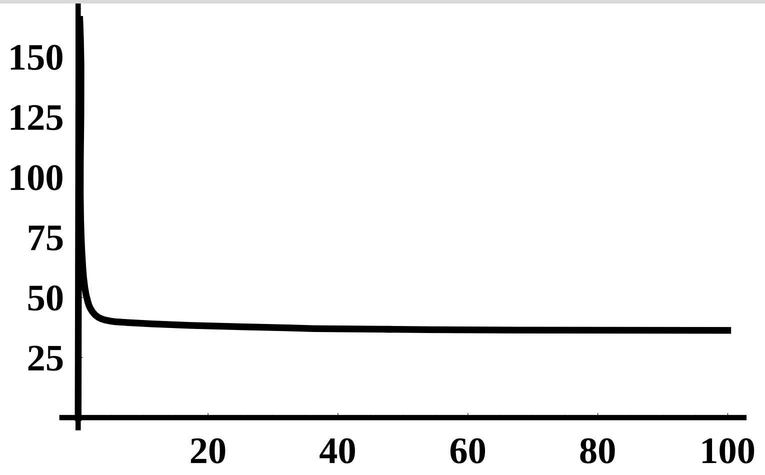


```
FormalPlot[Val, 100, All];
```

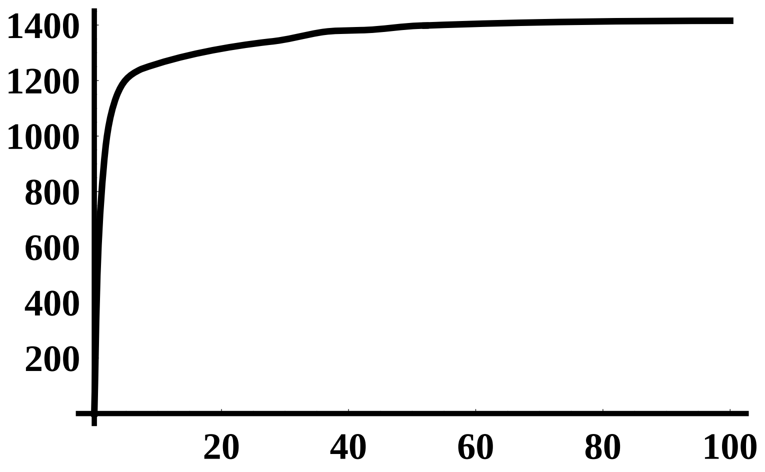

```
FormalPlot[aIPM, 100, All];
```

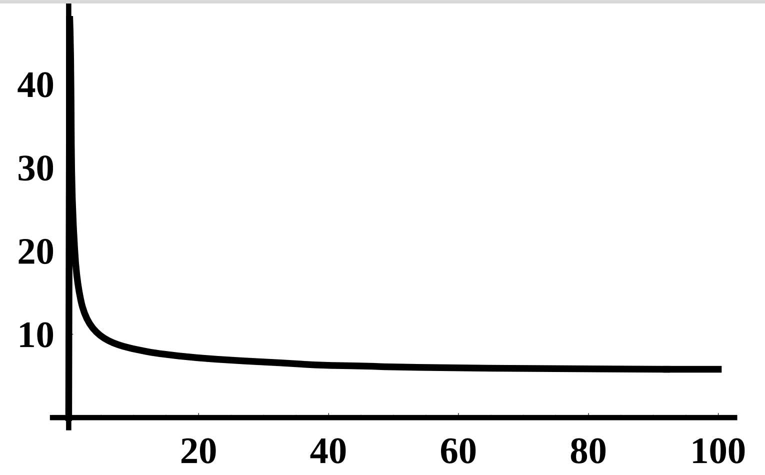


```
FormalPlot[bIPM, 100, All];
```

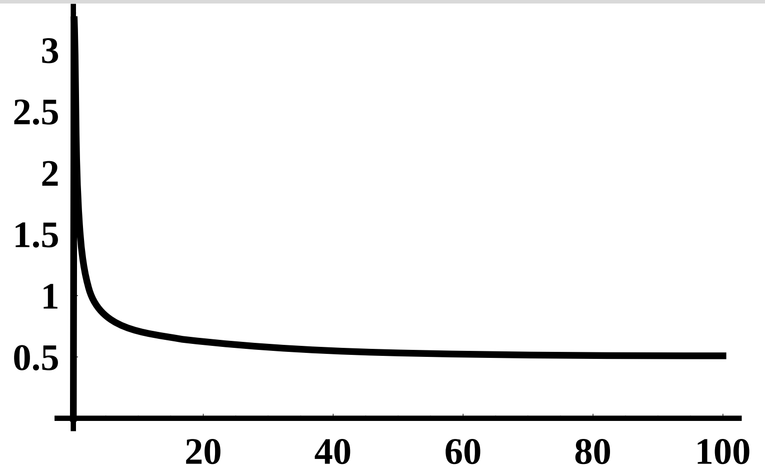

```
FormalPlot[aKIC, 100, All];
```

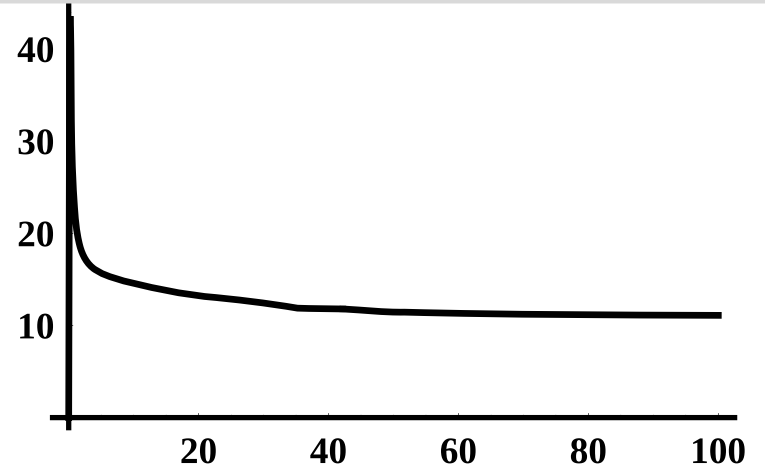


```
FormalPlot[Leu, 100, All];
```

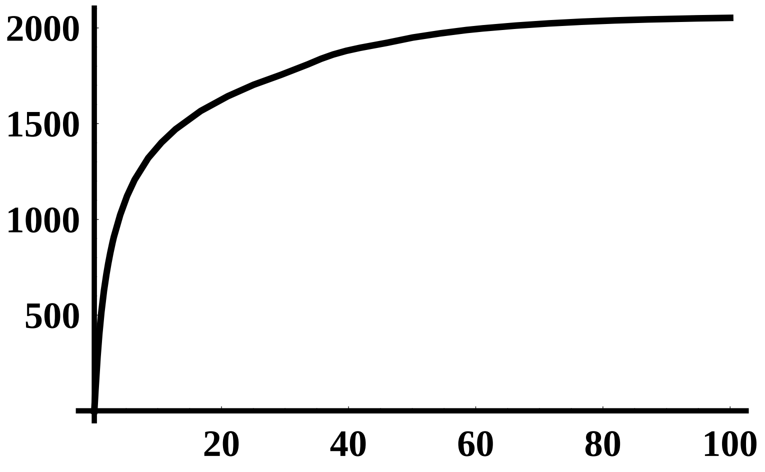

```
FormalPlot[Asp, 100, All];
```

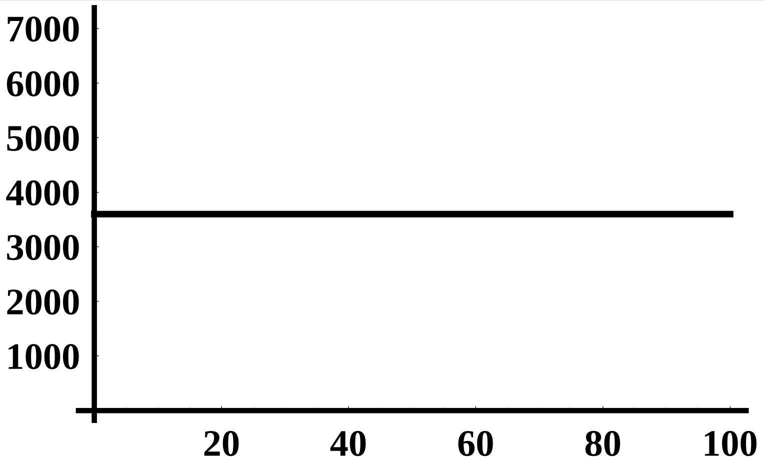


```
FormalPlot[AspP, 100, {0, 10}];
```

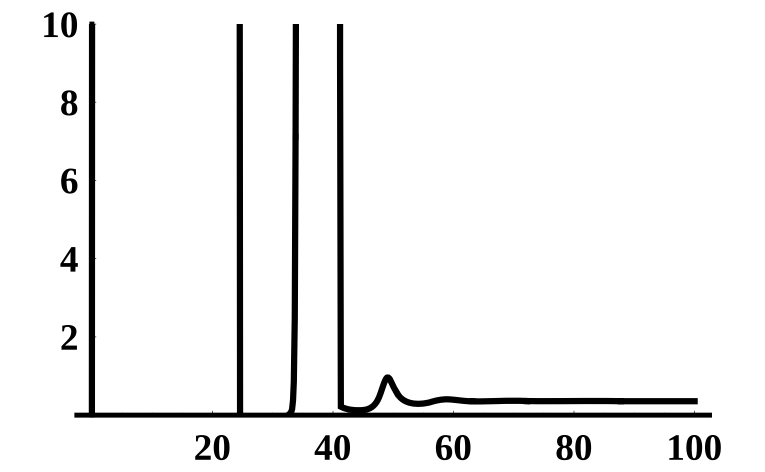

```
FormalPlot[ASA, 100, {0, 650}];
```

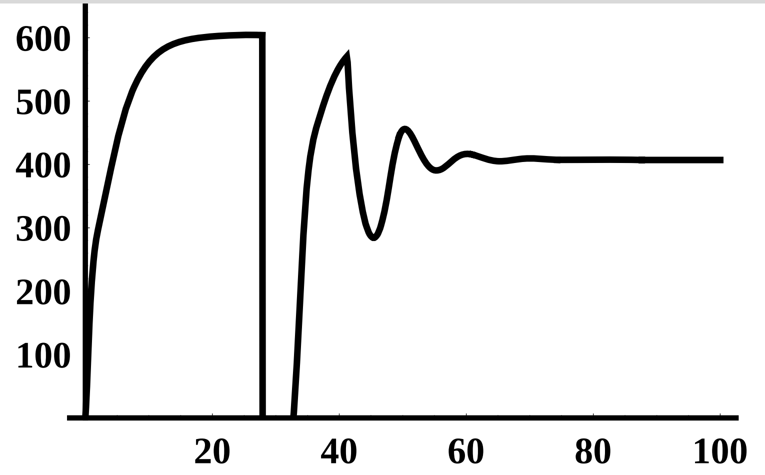


```
FormalPlot[Hse, 100, {0, 0.4}];
```

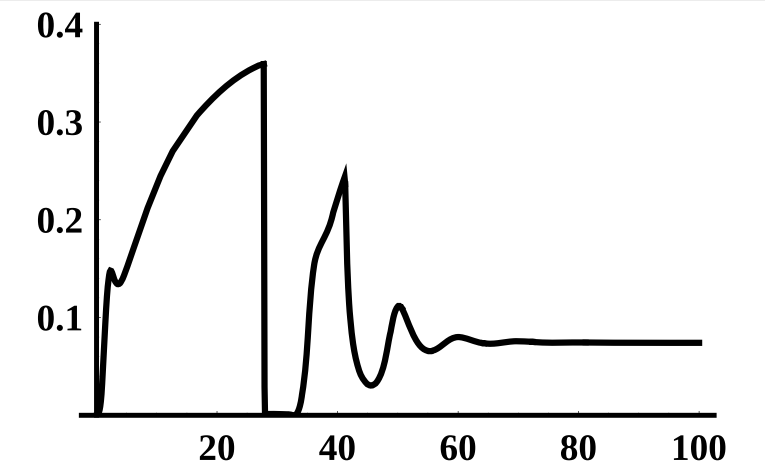

```
FormalPlot[HseP, 100, {0, 250}];
```

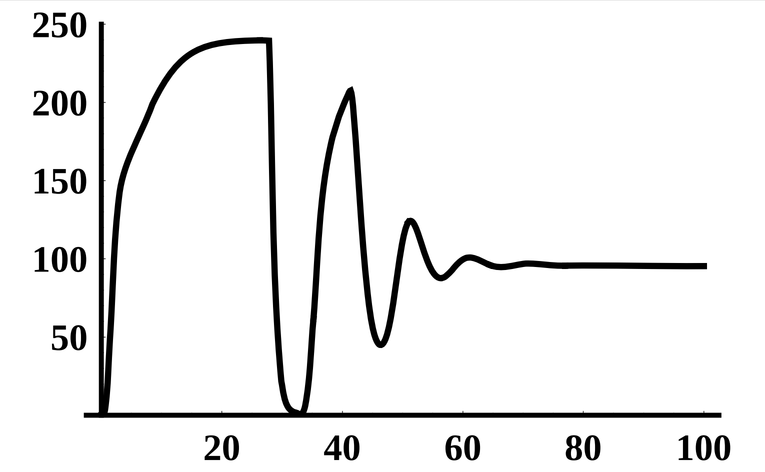


```
FormalPlot[Thr, 100, All];
```

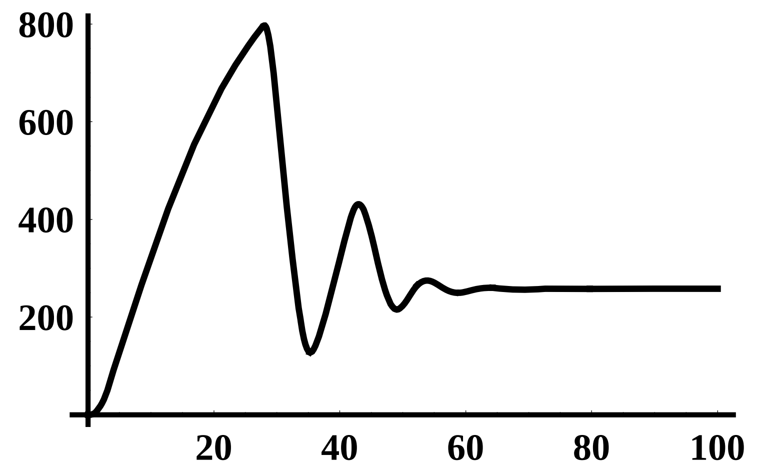


```
(* Pyr[0]= 1,000 *)
```

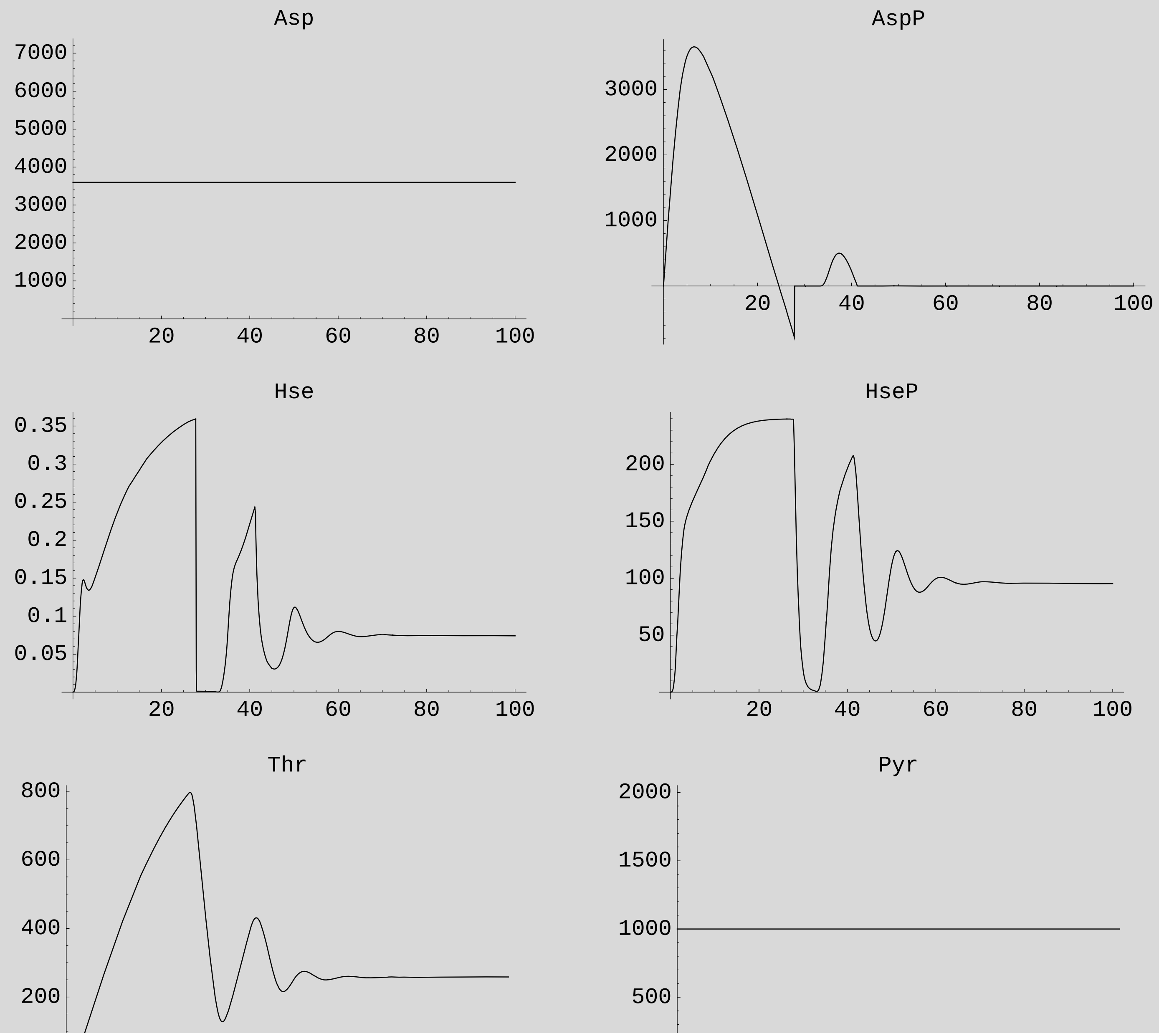
Asp
7000
6000
5000
4000
3000
2000
1000
20
40
60
80
100
AspP
3000
2000
1000
20
40
60
80
100
Hse
0.35
0.3
0.25
0.2
0.15
0.1
0.05
20
40
60
80
100
HseP
200
150
100
50
20
40
60
80
100
Thr
800
600
400
200
Pyr
2000
1500
1000
500

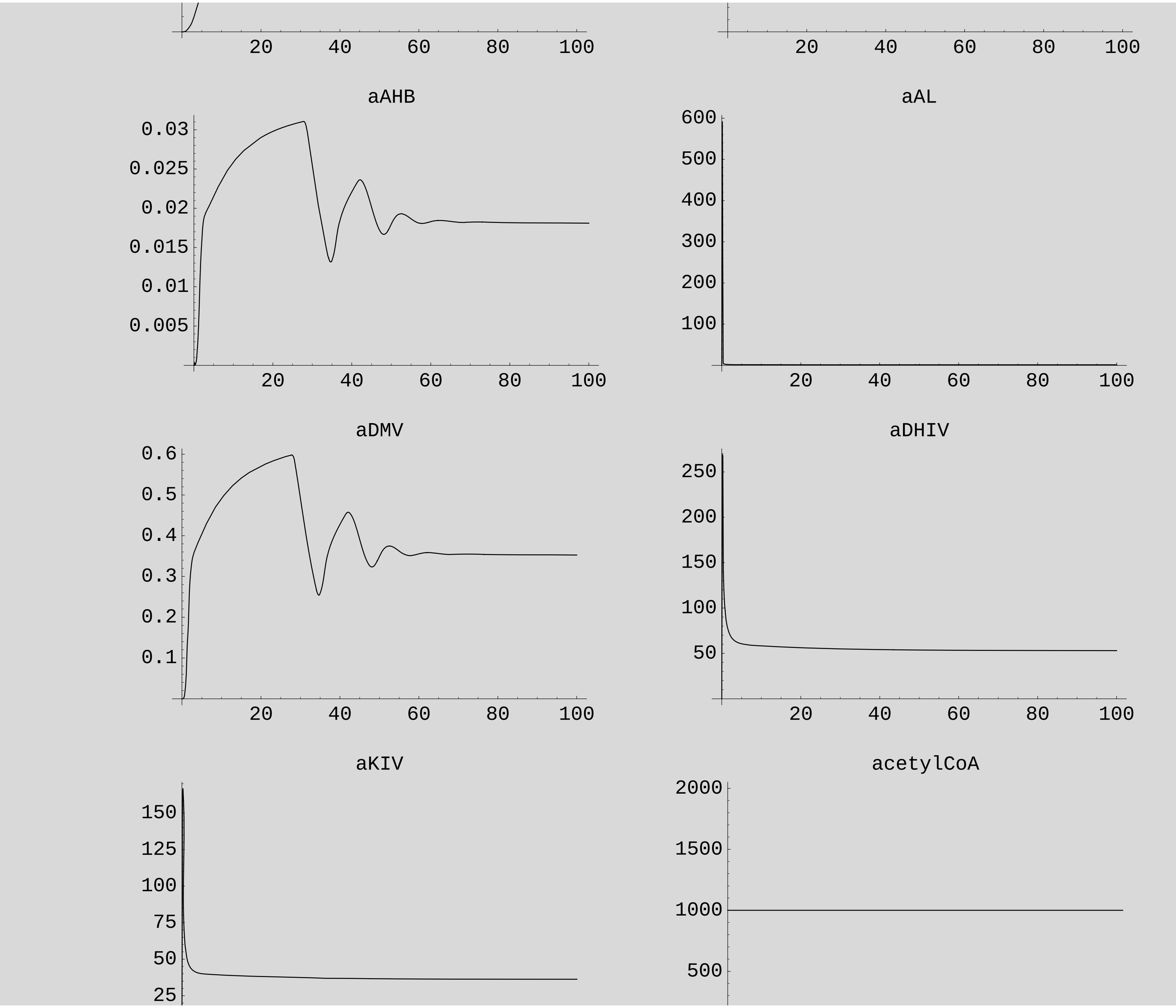
20 40 60 80 100
20 40 60 80 100
aAHB
0.03 0.025 0.02 0.015 0.01 0.005
20 40 60 80 100
aAL
600 500 400 300 200 100
20 40 60 80 100
aDMV
0.6 0.5 0.4 0.3 0.2 0.1
20 40 60 80 100
aDHIV
250 200 150 100 50
20 40 60 80 100
aKIV
150 125 100 75 50 25
acetylCoA
2000 1500 1000 500

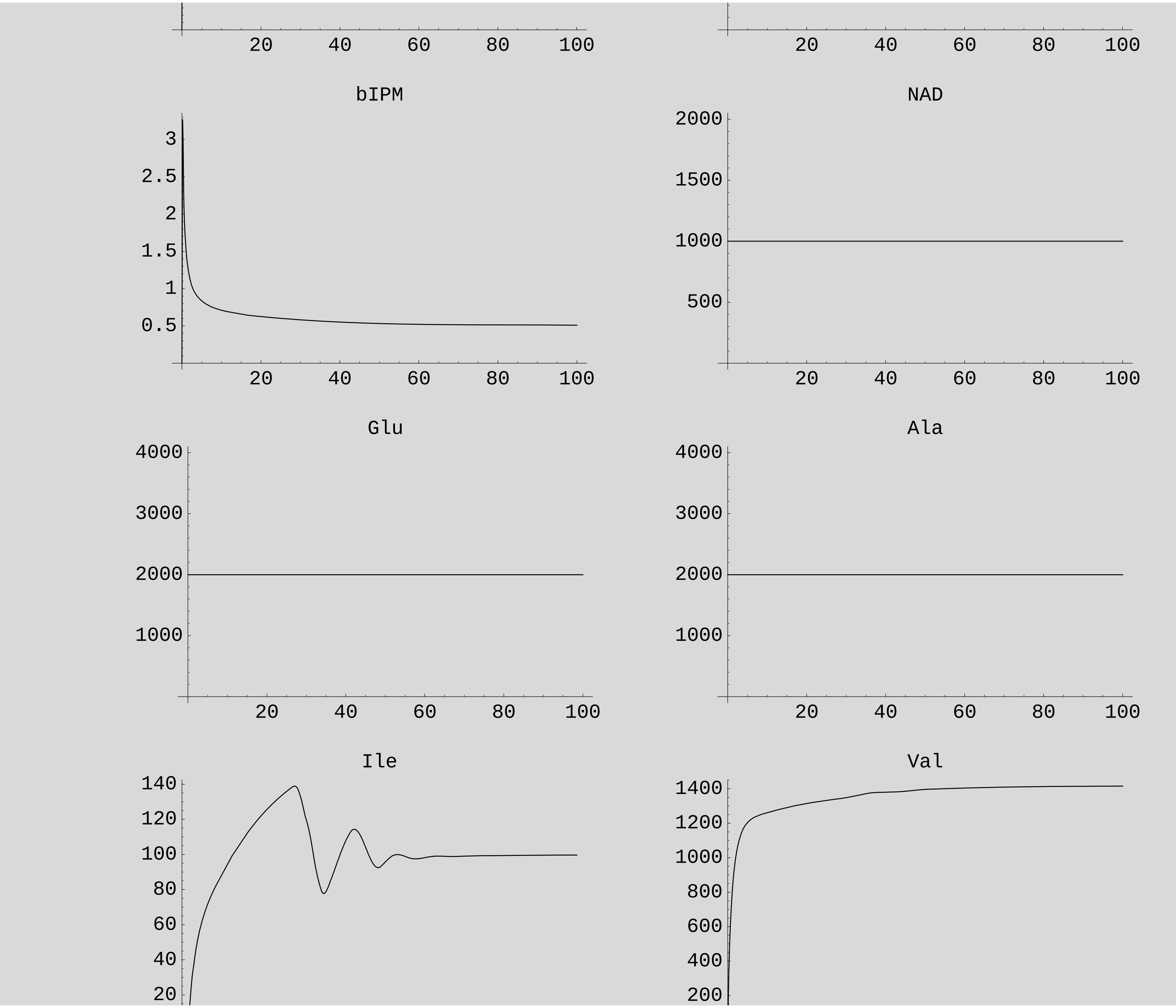
20 40 60 80 100
20 40 60 80 100
bIPM
3
2.5
2
1.5
1
0.5
20 40 60 80 100
NAD
2000
1500
1000
500
20 40 60 80 100
Glu
4000
3000
2000
1000
20 40 60 80 100
Ala
4000
3000
2000
1000
20 40 60 80 100
Ile
140
120
100
80
60
40
20
Val
1400
1200
1000
800
600
400
200

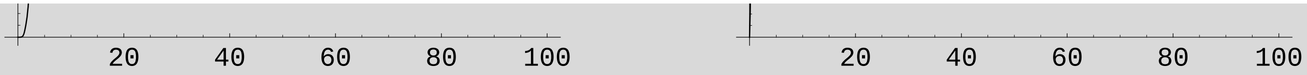
20
40
60
80
100
20
40
60
80
100

(* Pyr[0]= 10,000 *)

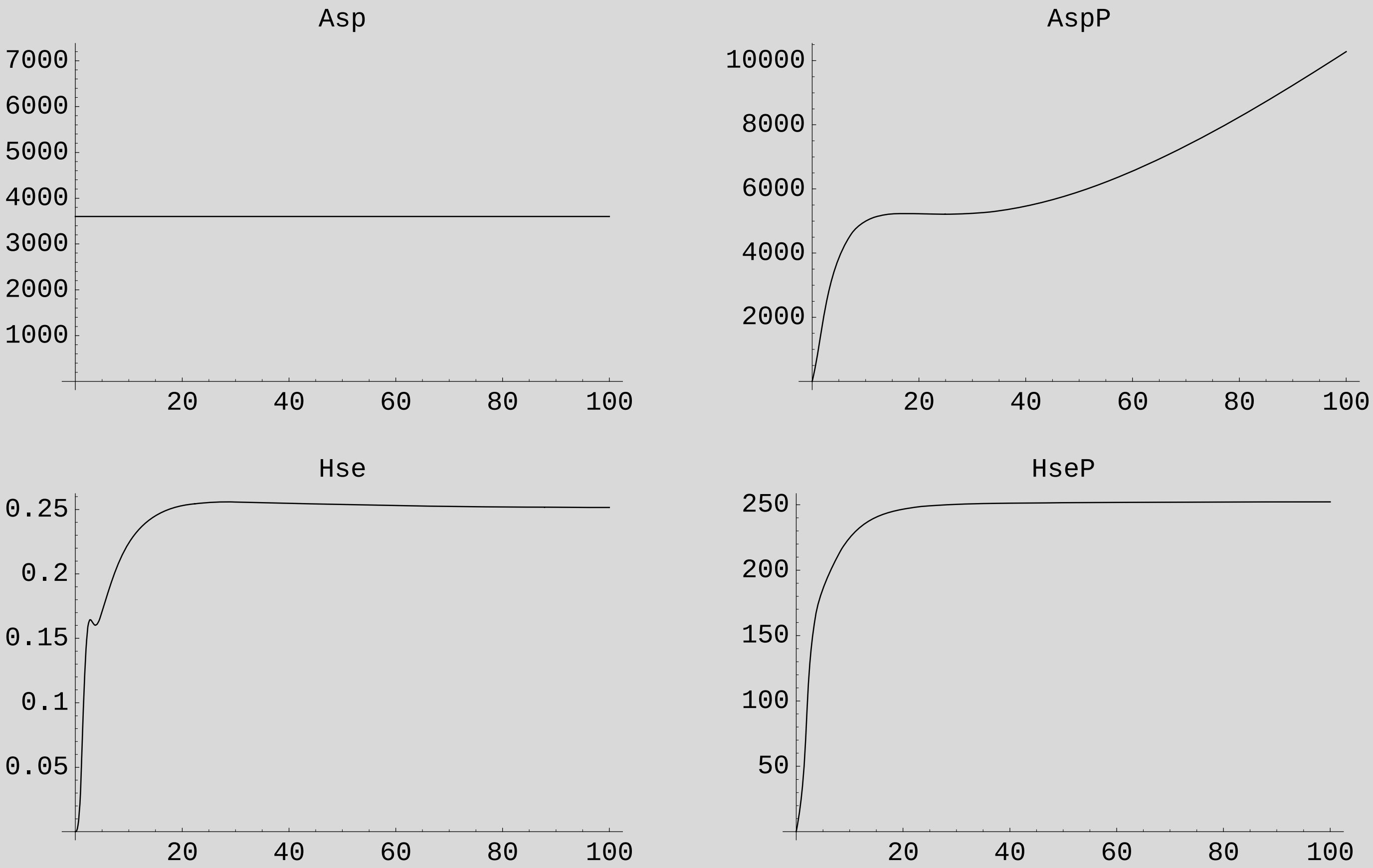
Asp
7000
6000
5000
4000
3000
2000
1000
20
40
60
80
100
AspP
10000
8000
6000
4000
2000
20
40
60
80
100
Hse
0.25
0.2
0.15
0.1
0.05
20
40
60
80
100
HseP
250
200
150
100
50
20
40
60
80
100

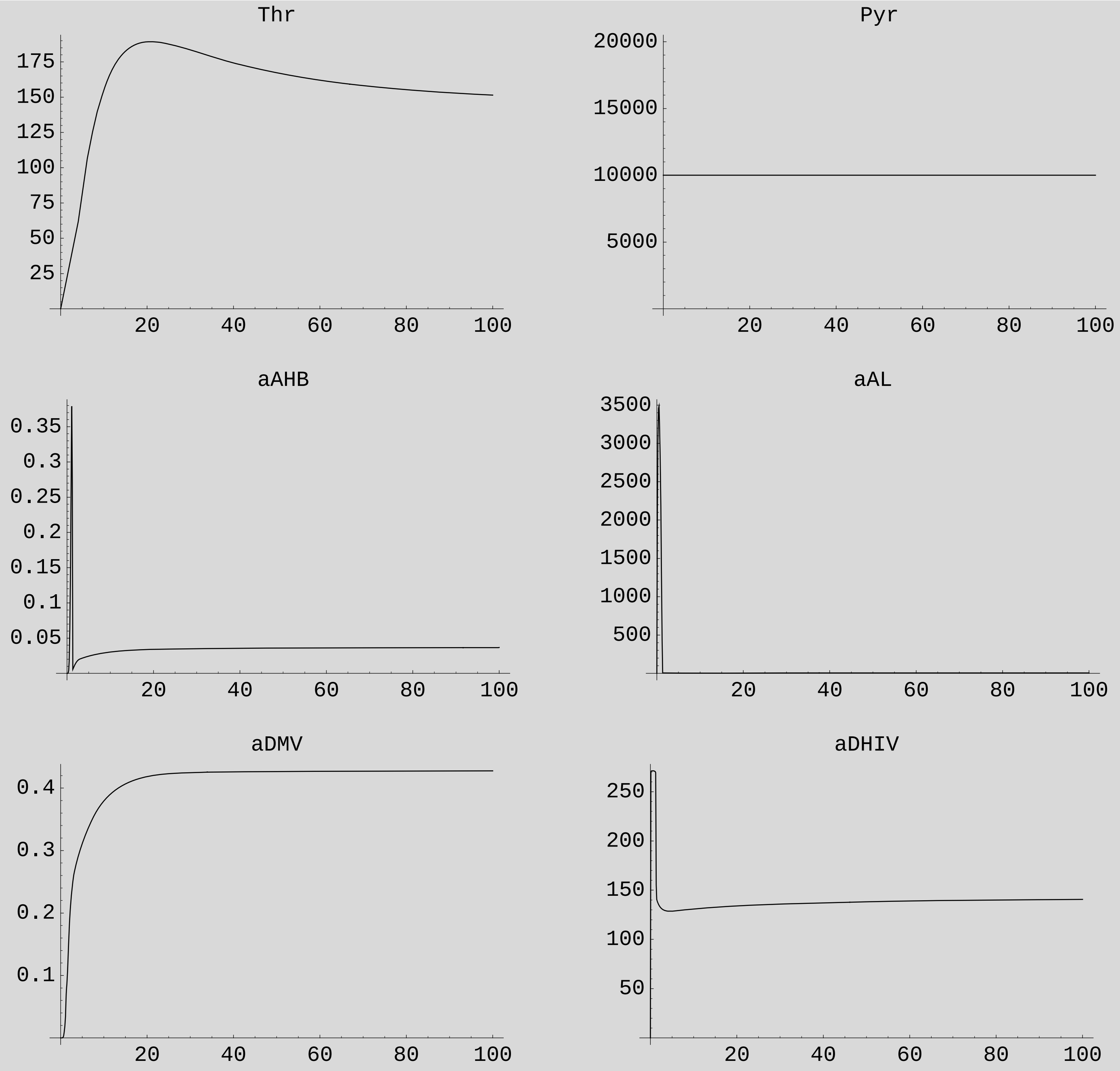
Thr
175
150
125
100
75
50
25
20
40
60
80
100
Pyr
20000
15000
10000
5000
20
40
60
80
100
aAHB
0.35
0.3
0.25
0.2
0.15
0.1
0.05
20
40
60
80
100
aAL
3500
3000
2500
2000
1500
1000
500
20
40
60
80
100
aDMV
0.4
0.3
0.2
0.1
20
40
60
80
100
aDHIV
250
200
150
100
50
20
40
60
80
100

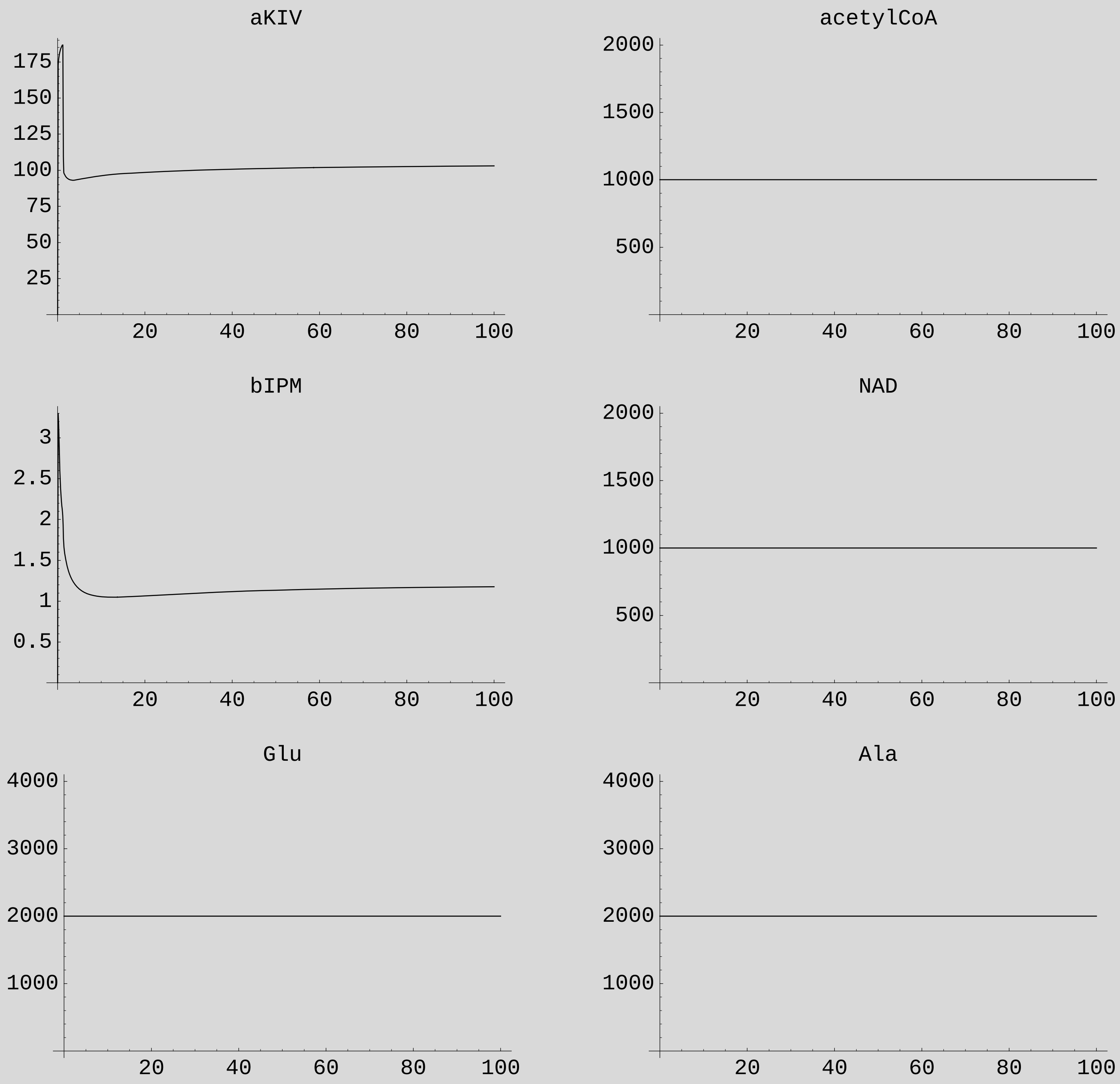
aKIV
175
150
125
100
75
50
25
20 40 60 80 100
acetylCoA
2000
1500
1000
500
20 40 60 80 100
bIPM
3
2.5
2
1.5
1
0.5
20 40 60 80 100
NAD
2000
1500
1000
500
20 40 60 80 100
Glu
4000
3000
2000
1000
20 40 60 80 100
Ala
4000
3000
2000
1000
20 40 60 80 100

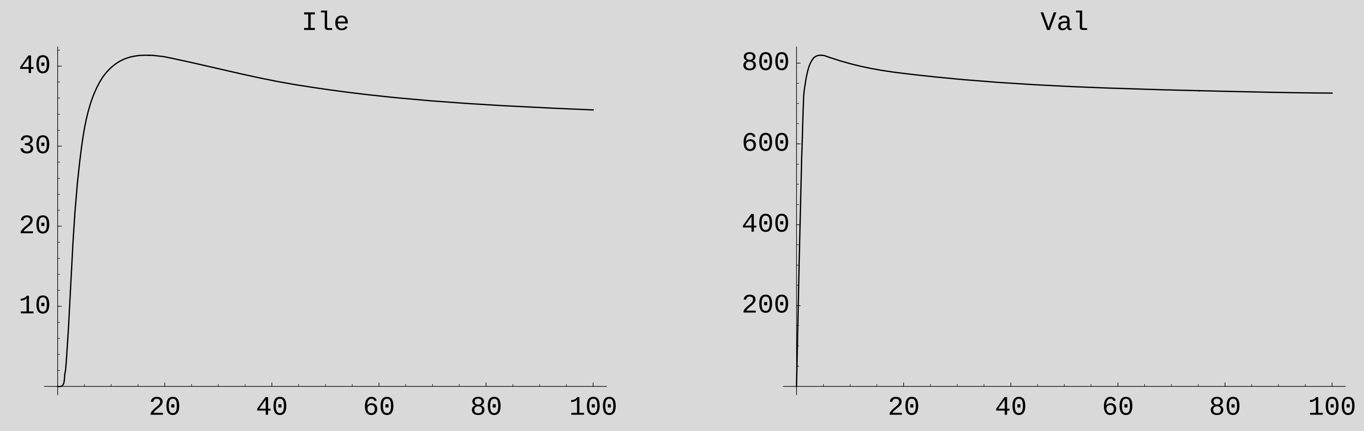


```
(*
  Display Simulations of Partition of Transaminase B and C
 X axis is Time (min), and Y axis is Concentration (μM)
 *)

displayTime = 100;
Show[
  GraphicsArray[
   Partition[
    Map[Plot[#[t] /. mySolution, {t, 0, displayTime}, PlotLabel → #, PlotRange → All,
       DisplayFunction → Identity] &, TBTCdistribution], 4]
  ]
 ];
```

```
(* Pyr[0]= 1,000 *)
```

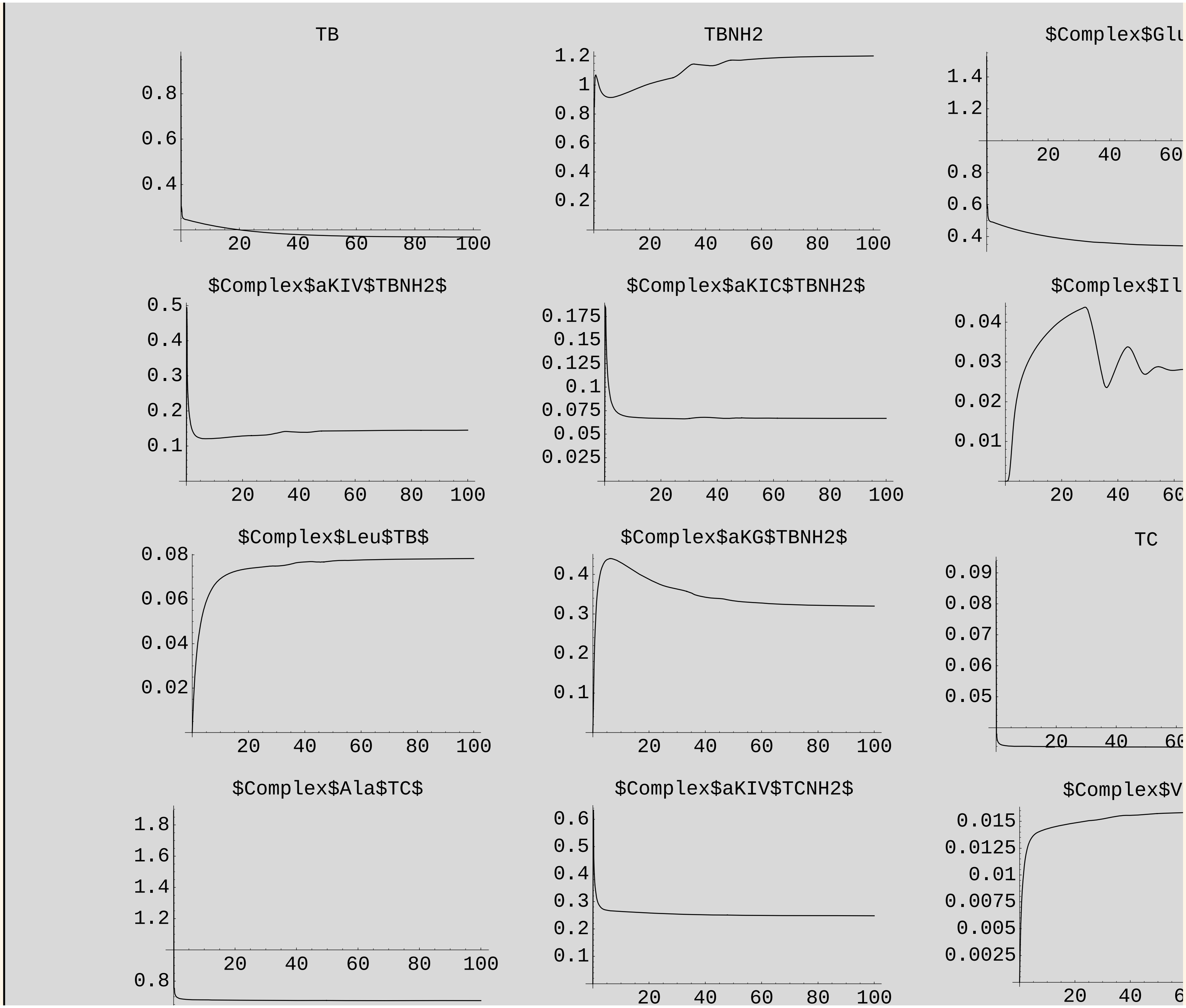
TB
0.8
0.6
0.4
20 40 60 80 100
TBNH2
1.2
1
0.8
0.6
0.4
0.2
20 40 60 80 100
$Complex$Glu
1.4
1.2
20 40 60
0.8
0.6
0.4
$Complex$aKIV$TBNH2$
0.5
0.4
0.3
0.2
0.1
20 40 60 80 100
$Complex$aKIC$TBNH2$
0.175
0.15
0.125
0.1
0.075
0.05
0.025
20 40 60 80 100
$Complex$Il
0.04
0.03
0.02
0.01
20 40 60
$Complex$Leu$TB$
0.08
0.06
0.04
0.02
20 40 60 80 100
$Complex$aKG$TBNH2$
0.4
0.3
0.2
0.1
20 40 60 80 100
TC
0.09
0.08
0.07
0.06
0.05
20 40 60
$Complex$Ala$TC$
1.8
1.6
1.4
1.2
20 40 60 80 100
0.8
$Complex$aKIV$TCNH2$
0.6
0.5
0.4
0.3
0.2
0.1
20 40 60 80 100
$Complex$V
0.015
0.0125
0.01
0.0075
0.005
0.0025
20 40

(* Pyr[0]= 10,000 *)

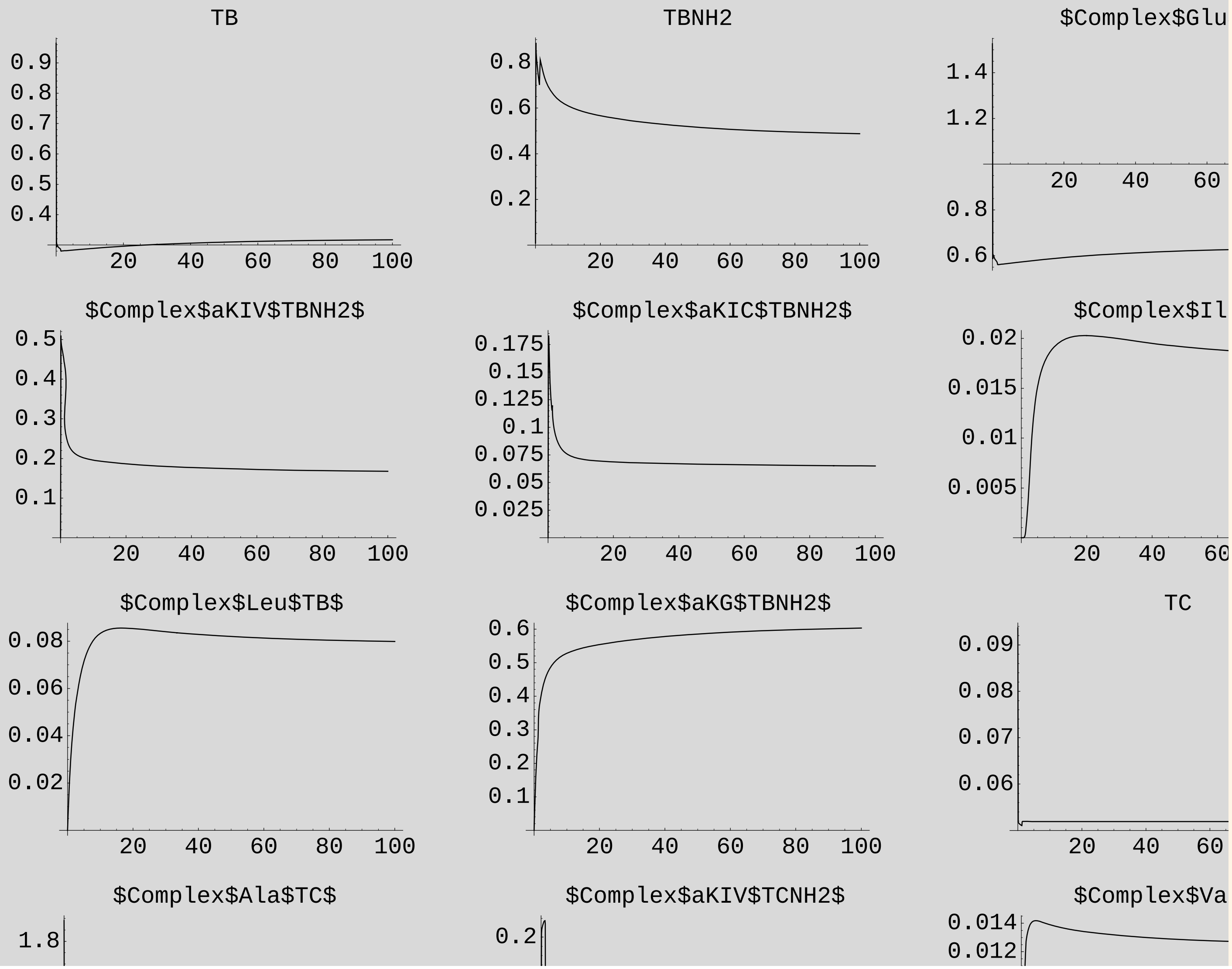
TB
TBNH2
$Complex$Glu
$Complex$aKIV$TBNH2$
$Complex$aKIC$TBNH2$
$Complex$Il
$Complex$Leu$TB$
$Complex$aKG$TBNH2$
TC
$Complex$Ala$TC$
$Complex$aKIV$TCNH2$
$Complex$Va

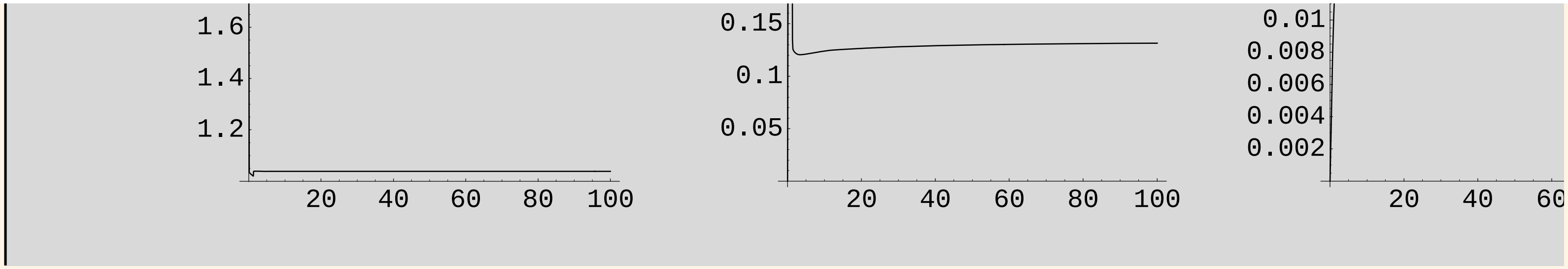
1.6
1.4
1.2
20 40 60 80 100
0.15
0.1
0.05
20 40 60 80 100
0.01
0.008
0.006
0.004
0.002
20 40 60
