# Supplementary material for: An enzyme-centric approach for modelling non-linear biological complexity
Source: BMC Syst Biol. 2008 Aug 1;2:70. doi: 10.1186/1752-0509-2-70 (PMC3146071; doi:10.1186/1752-0509-2-70)
Supplement: Additional file 2 — Threonine Deaminase in Salmonella typhimurium. [file 1752-0509-2-70-S2.pdf]

Yang, Additional File 1

A. Curve fitting for the allosteric parameters of threonine deaminase in *Salmonella typhimurium.*

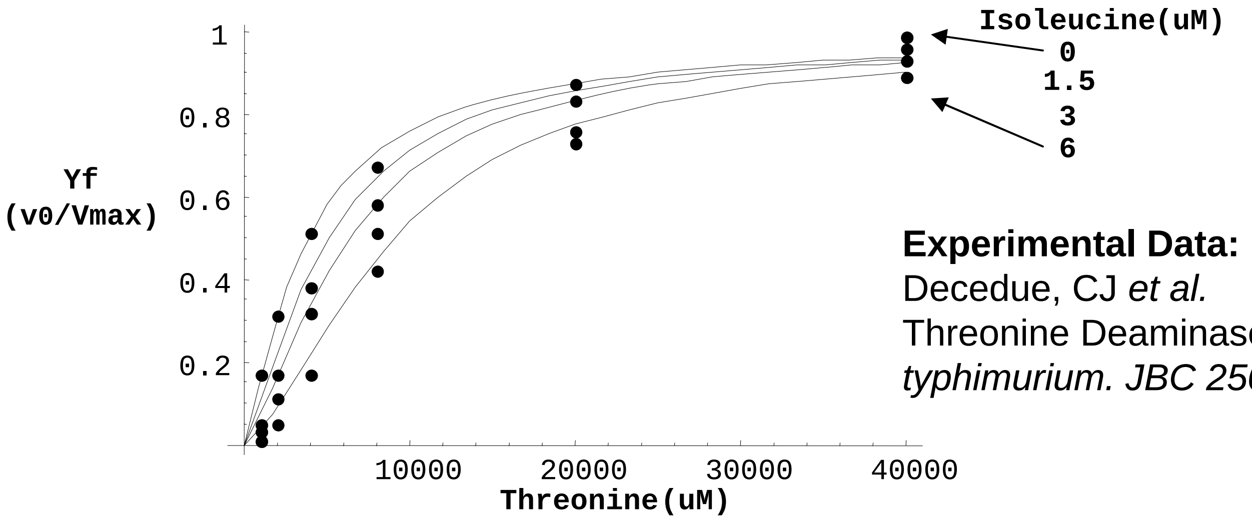

Experimental Data:
Decedue, CJ *et al.*
Threonine Deaminase from *Salmonella typhimurium. JBC 250(4):1563-70, 1975*

B. Toxic Accumulation of α-Ketobutyrate Caused by Inhibition of the Branched-Chain Amino Acid Biosynthetic Enzyme Acetolactate Synthase in *Salmonella typhimurium*.

LaRossa *et al*. DuPont Co. Inc. *J. Bact.* 169: 1372-1378 (1987)

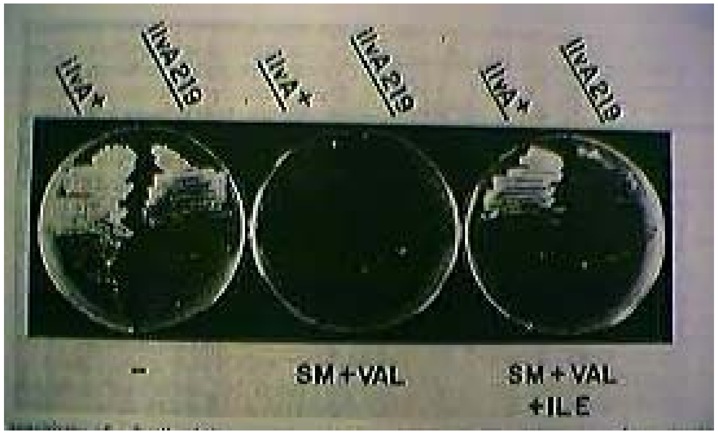

ilvA+: wild-type

ilvA219: threonine deaminase mutant, resistant to isoleucine feedback inhibition

Isoleucine Rescue of Sulfometuron Methyl and Valine Growth Inhibition
