# Supplementary material for: An enzyme-centric approach for modelling non-linear biological complexity
Source: BMC Syst Biol. 2008 Aug 1;2:70. doi: 10.1186/1752-0509-2-70 (PMC3146071; doi:10.1186/1752-0509-2-70)
Supplement: Additional file 3 — The Lambda (Λ) approximation and Langevin equation. [file 1752-0509-2-70-S3.pdf]

Yang, Additional File 3: Λ Approximation and Langevin Equation

A. Λ **Approximation** (Yang et al. *Bioinformatics* 2005, 21(6):774-780) :
While increasing the values of Λ, reaction curves converge to the *Michaelis-Menten* equation

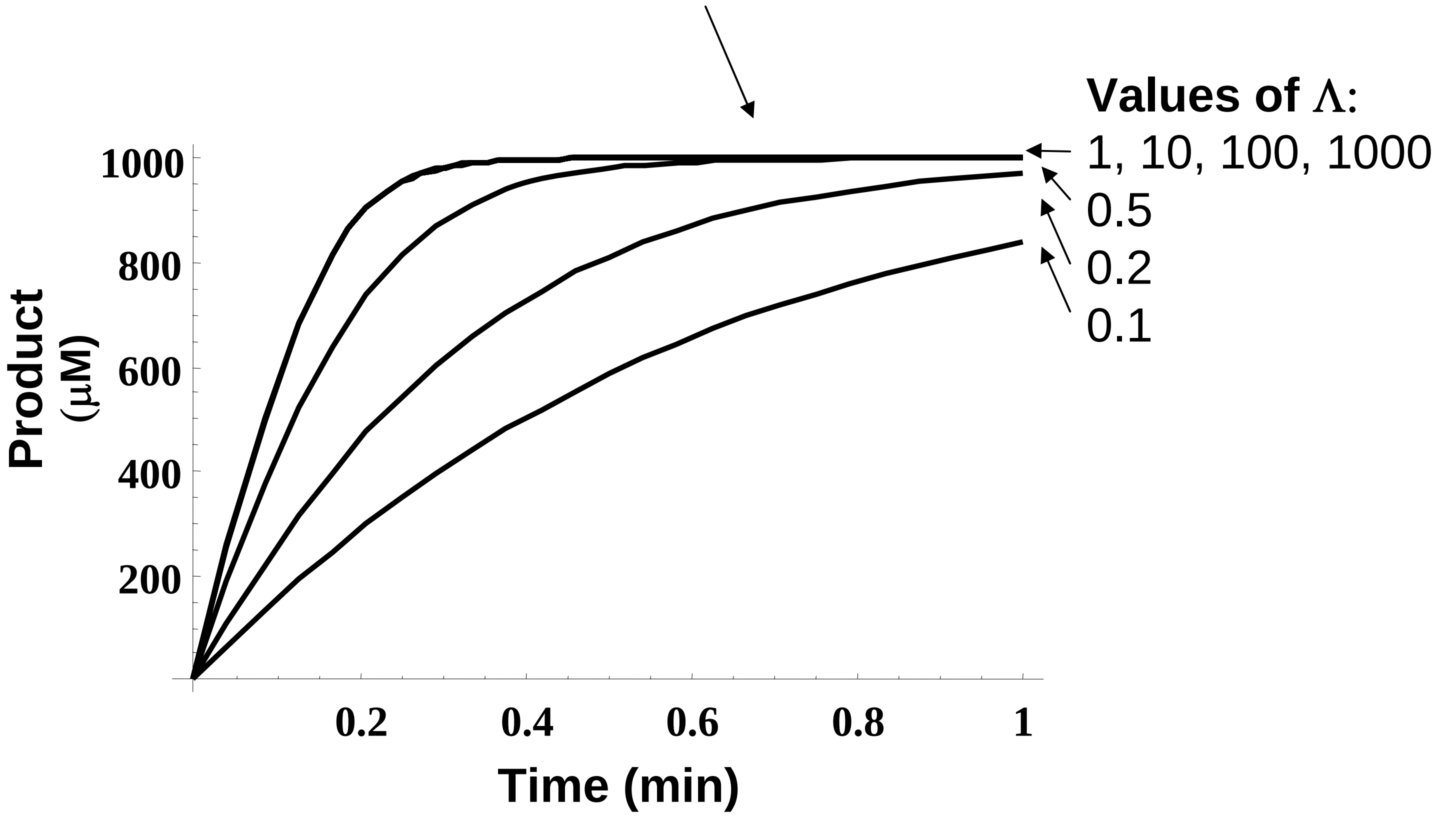

B. Langevin Equation: the probabilistic perturbations of transcription and splicing rates to simulate the experimental variations shown in Figure 4D.

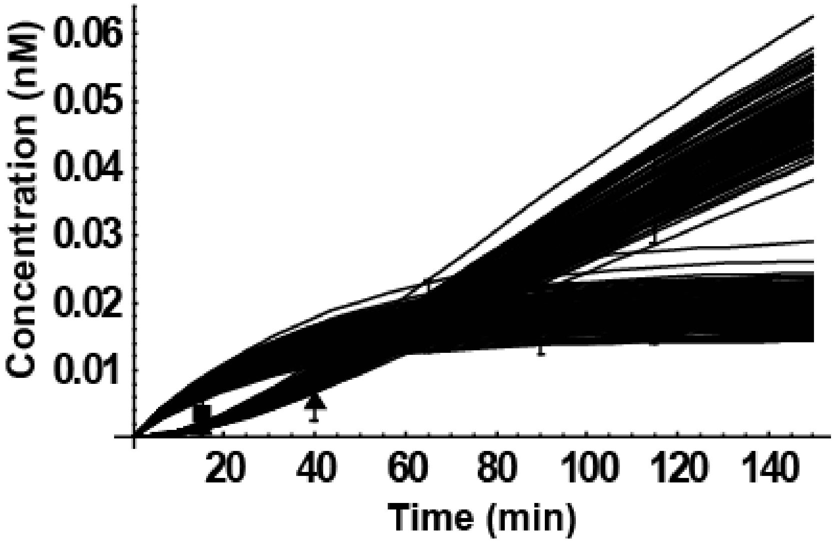

Notes:
1. Each line represents one stochastic simulation over time.
2. The overlapping lines represent the range of variations corresponding to the experimental error bars in Figure 4D.
