# Supplementary material for: An enzyme-centric approach for modelling non-linear biological complexity
Source: BMC Syst Biol. 2008 Aug 1;2:70. doi: 10.1186/1752-0509-2-70 (PMC3146071; doi:10.1186/1752-0509-2-70)
Supplement: Additional file 4 — Mathematica™ codes and parameter values for the RNA Splicing model. [file 1752-0509-2-70-S4.pdf]

```
<< myPadRight.m;
<< cellerator.m;
<< kMech_Rev.m;
Off[General::"spell1"];
Off[General::"spell"];
```

```
myPadRight Version 0.3 for Cellerator Loaded.
```

```
Cellerator™ 1.4.6 (30-July-2004) loaded 8-Nov-2004 15:38
  using Mathematica Version 5.0 for Microsoft Windows (June 11, 2003)
```

```
 kMech is loaded
```

```
(* Start mRNA Splcing Simulation *)
(* NE1: RNA polymerase; NEs: spliceosome; NEd: degradation enzymes *)

mRNASplcing = Union[
  Enz[{DNA, NTP} ⇄(NE1) {premRNA, DNA}, BiBi[kf1, kr2, kcat3]],
  {{premRNA + NEs ⇄ $Complex$NEs$premRNA$, kf4, kr5}},
  {{$Complex$NEs$premRNA$ → NEs + mRNA, kcat6}},
  {{premRNA ⇄(NEd) NMP, kf7, kr8, kcat9}},
  {{$Complex$NEs$premRNA$ ⇄(NEd) NMP, kf10, kr11, kcat12}},
  {{mRNA ⇄(NEd) NMP, kf13, kr14, kcat15}}
 ]

{{$Complex$NE1$DNA$NTP$ → DNA + NE1 + premRNA, kcat3},
 {$Complex$NEs$premRNA$ → mRNA + NEs, kcat6}, {DNA + NE1 + NTP ⇄ $Complex$NE1$DNA$NTP$, kf1, kr2},
 {NEs + premRNA ⇄ $Complex$NEs$premRNA$, kf4, kr5}, {mRNA ⇄(NEd) NMP, kf13, kr14, kcat15},
 {premRNA ⇄(NEd) NMP, kf7, kr8, kcat9}, {$Complex$NEs$premRNA$ ⇄(NEd) NMP, kf10, kr11, kcat12}}
```

```
{myODEs,myVars}=interpret[mRNASplcing]

{{DNA′[t] ==
    -kf1 DNA[t] NE1[t] NTP[t] + kcat3 $Complex$NE1$DNA$NTP$[t] + kr2 $Complex$NE1$DNA$NTP$[t],
  mRNA′[t] == -kf13 mRNA[t] NEd[t] + kr14 $Complex$mRNA$NEd$[t] + kcat6 $Complex$NEs$premRNA$[t],
  NE1′[t] ==
    -kf1 DNA[t] NE1[t] NTP[t] + kcat3 $Complex$NE1$DNA$NTP$[t] + kr2 $Complex$NE1$DNA$NTP$[t],
  NEd′[t] == -kf13 mRNA[t] NEd[t] - kf7 NEd[t] premRNA[t] + kcat15 $Complex$mRNA$NEd$[t] +
     kr14 $Complex$mRNA$NEd$[t] - kf10 NEd[t] $Complex$NEs$premRNA$[t] +
     kcat9 $Complex$premRNA$NEd$[t] + kr8 $Complex$premRNA$NEd$[t] +
     kcat12 $Complex$$Complex$NEs$premRNA$$NEd$[t] + kr11 $Complex$$Complex$NEs$premRNA$$NEd$[t],
  NEs′[t] == -kf4 NEs[t] premRNA[t] + kcat6 $Complex$NEs$premRNA$[t] + kr5 $Complex$NEs$premRNA$[t],
  NMP′[t] == kcat15 $Complex$mRNA$NEd$[t] + kcat9 $Complex$premRNA$NEd$[t] +
     kcat12 $Complex$$Complex$NEs$premRNA$$NEd$[t],
  NTP′[t] == -kf1 DNA[t] NE1[t] NTP[t] + kr2 $Complex$NE1$DNA$NTP$[t],
  premRNA′[t] == -kf7 NEd[t] premRNA[t] - kf4 NEs[t] premRNA[t] + kcat3 $Complex$NE1$DNA$NTP$[t] +
     kr5 $Complex$NEs$premRNA$[t] + kr8 $Complex$premRNA$NEd$[t], $Complex$mRNA$NEd$′[t] ==
    kf13 mRNA[t] NEd[t] - kcat15 $Complex$mRNA$NEd$[t] - kr14 $Complex$mRNA$NEd$[t],
  $Complex$NE1$DNA$NTP$′[t] == kf1 DNA[t] NE1[t] NTP[t] - kcat3 $Complex$NE1$DNA$NTP$[t] -
     kr2 $Complex$NE1$DNA$NTP$[t], $Complex$NEs$premRNA$′[t] ==
    kf4 NEs[t] premRNA[t] - kcat6 $Complex$NEs$premRNA$[t] - kr5 $Complex$NEs$premRNA$[t] -
     kf10 NEd[t] $Complex$NEs$premRNA$[t] + kr11 $Complex$$Complex$NEs$premRNA$$NEd$[t],
  $Complex$premRNA$NEd$′[t] == kf7 NEd[t] premRNA[t] - kcat9 $Complex$premRNA$NEd$[t] -
     kr8 $Complex$premRNA$NEd$[t], $Complex$$Complex$NEs$premRNA$$NEd$′[t] ==
    kf10 NEd[t] $Complex$NEs$premRNA$[t] - kcat12 $Complex$$Complex$NEs$premRNA$$NEd$[t] -
     kr11 $Complex$$Complex$NEs$premRNA$$NEd$[t]},
 {DNA, mRNA, NE1, NEd, NEs, NMP, NTP, premRNA, $Complex$mRNA$NEd$,
  $Complex$NE1$DNA$NTP$, $Complex$NEs$premRNA$,
  $Complex$premRNA$NEd$, $Complex$$Complex$NEs$premRNA$$NEd$}}
```

```
Lamda = 1;

myKs = {

  KmNE1$DNA = 40; (*2;*) KmNE1$NTP = 1000; kcat3 = 0.1; (*0.6;*)
  kf1 → Kf2S[KmNE1$DNA, KmNE1$NTP, kcat3, Lamda],
  kr2 → Kr[kcat3, Lamda],

  KmNEs$premRNA = 3.5; (*15;*) kcat6 = 0.03; (*0.1;*)
  kf4 → Kf[KmNEs$premRNA, kcat6, Lamda],
  kr5 → Kr[kcat6, Lamda],

  KmNEd$premRNA = 2000000 (*10 1*); kcat9 = 30000 (*3000*);
  kf7 → Kf[KmNEd$premRNA, kcat9, Lamda],
  kr8 → Kr[kcat9, Lamda],

  KmNEd$Complex$NEs$premRNA = 30000000; kcat12 = 30000;
  kf10 → Kf[KmNEd$Complex$NEs$premRNA, kcat12, Lamda],
  kr11 → Kr[kcat12, Lamda],

  KmNEd$mRNA = 30000000; (* 20 X KmNEd$premRNA *) kcat15 = 30000;
  kf13 → Kf[KmNEd$mRNA, kcat15, Lamda],
  kr14 → Kr[kcat15, Lamda]
 }
```

{kf1 → 2.5×10^{-6}, kr2 → 0, kf4 → 0.00857143, kr5 → 0,
kf7 → 0.015, kr8 → 0, kf10 → 0.001, kr11 → 0, kf13 → 0.001, kr14 → 0}

```
myICs = {
   DNA[0] == 12,
   NTP[0] == 25000,
   premRNA[0] == 0,
   mRNA[0] == 0,
   NMP[0] == 0,

   NE1[0] == 0.025, (*0.003,*)
   NEs[0] == 5, (*0.006,*)
   NEd[0] == 3.5, (*1 0.2,*)

   $Complex$NE1$DNA$NTP$[0] == 0,
   $Complex$NEs$premRNA$[0] == 0,
   $Complex$premRNA$NEd$[0] == 0,
   $Complex$$Complex$NEs$premRNA$$NEd$[0] == 0,
   $Complex$mRNA$NEd$[0] == 0
  };
```

```
tmax = 165;
mySolution = NDSolve[Join[myODEs /. myKs, myICs], myVars, {t, 0, tmax}, MaxSteps → 3000]

{{DNA → InterpolatingFunction[{{0., 165.}}, <>],
  mRNA → InterpolatingFunction[{{0., 165.}}, <>], NE1 → InterpolatingFunction[{{0., 165.}}, <>],
  NEd → InterpolatingFunction[{{0., 165.}}, <>], NEs → InterpolatingFunction[{{0., 165.}}, <>],
  NMP → InterpolatingFunction[{{0., 165.}}, <>], NTP → InterpolatingFunction[{{0., 165.}}, <>],
  premRNA → InterpolatingFunction[{{0., 165.}}, <>],
  $Complex$mRNA$NEd$ → InterpolatingFunction[{{0., 165.}}, <>],
  $Complex$NE1$DNA$NTP$ → InterpolatingFunction[{{0., 165.}}, <>],
  $Complex$NEs$premRNA$ → InterpolatingFunction[{{0., 165.}}, <>],
  $Complex$premRNA$NEd$ → InterpolatingFunction[{{0., 165.}}, <>],
  $Complex$$Complex$NEs$premRNA$$NEd$ → InterpolatingFunction[{{0., 165.}}, <>]}}
```

```
orderedVars={DNA,NTP,premRNA,mRNA,NMP,NE1,NEs,NEd,$Complex$NE1$DNA$NTP$,
$Complex$NEs$premRNA$,$Complex$premRNA$NEd$,$Complex$mRNA$NEd$};

Show[GraphicsArray[
    Partition[
      Map[Plot[#[t]/.mySolution,{t,0,tmax},PlotLabel→#,
            PlotRange→All,DisplayFunction→Identity]&,orderedVars],3]
    ]
  ];
```

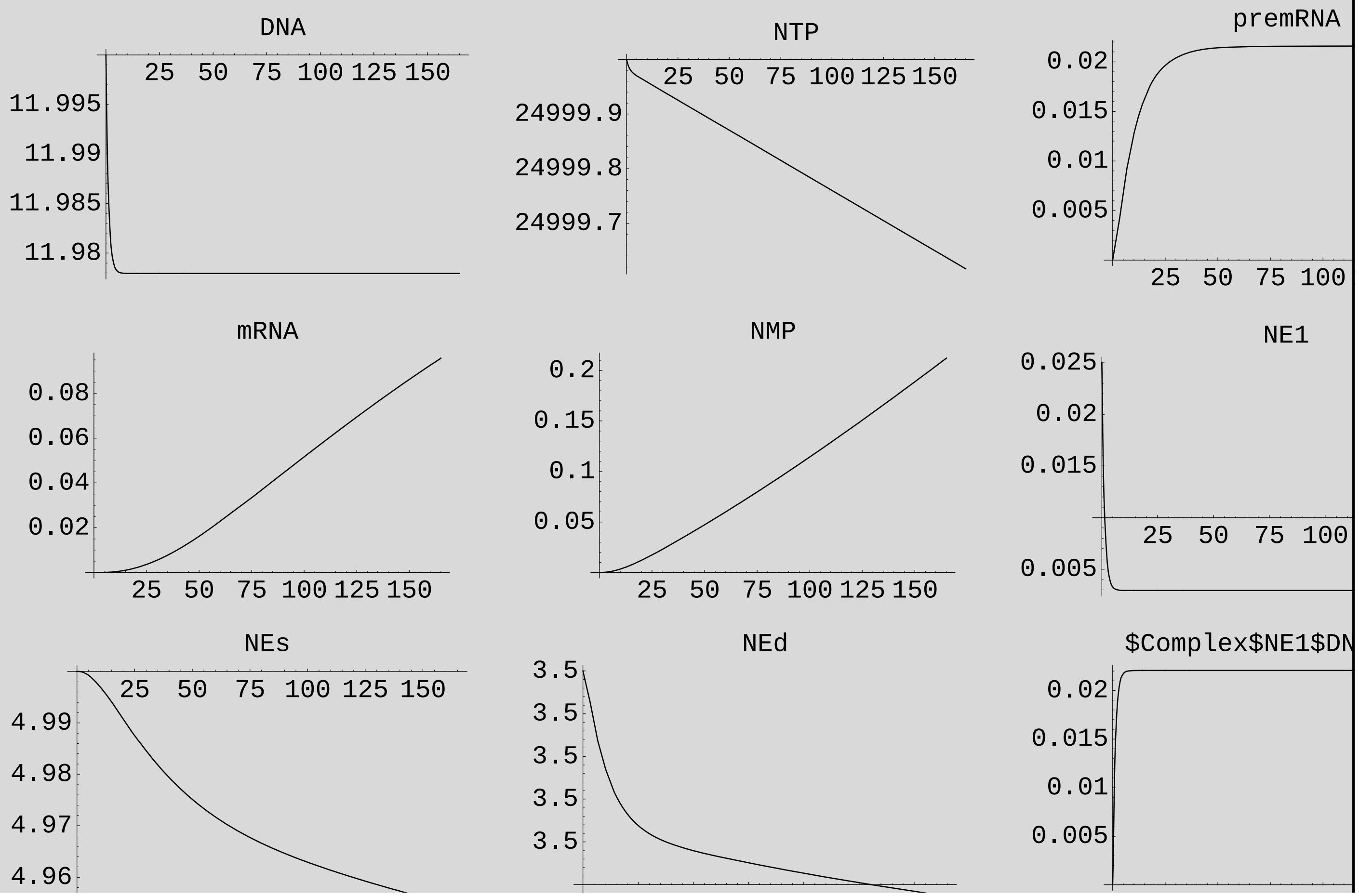

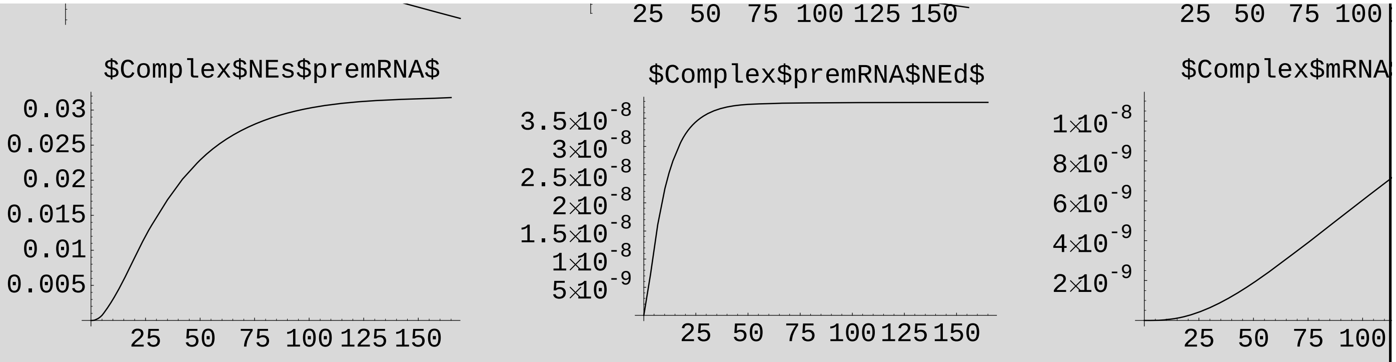


```
totalPremRNA[t_] = premRNA[t] + $Complex$NEs$premRNA$[t] + $Complex$premRNA$NEd$[t];
```

```
P1 = Plot[mRNA[t] /. mySolution, {t, 0, tmax}, PlotRange → All, DisplayFunction → Identity];
P2 = Plot[totalPremRNA[t] /. mySolution,
   {t, 0, tmax}, PlotRange → All, DisplayFunction → Identity];
Show[P1, P2, AxesLabel → {"Time (min) ", nM}, PlotLabel → "RNA Splicing",
  DisplayFunction → $DisplayFunction];
```

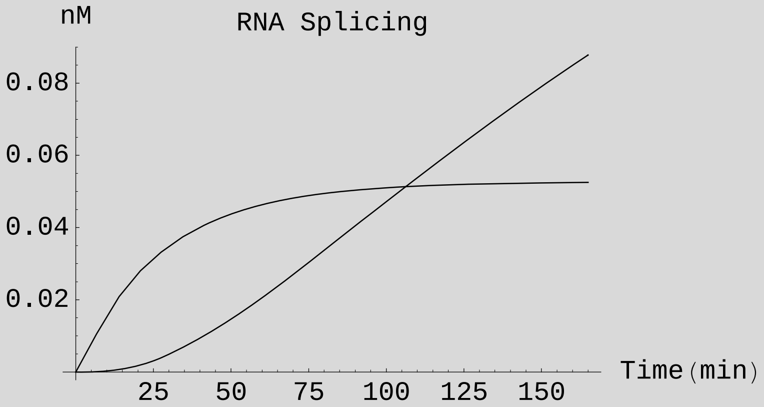

```
P3 = ListPlot[{

    (* Time points (min): 15,40,65,90,115,140,165 *)
    (* mRNA concentration (nM) *)
    {15, 0.0019}, {40, 0.0081},
    {65, 0.024}, {90, 0.041}, {115, 0.057}, {140, 0.0725}, {165, 0.084},

    (* pre-mRNA concentration (nM) *)
    {15, 0.0139}, {40, 0.04}, {65, 0.056}, {90, 0.053}, {115, 0.052}, {140, 0.055}, {165, 0.056}

  }, Prolog → AbsolutePointSize[5]];
```

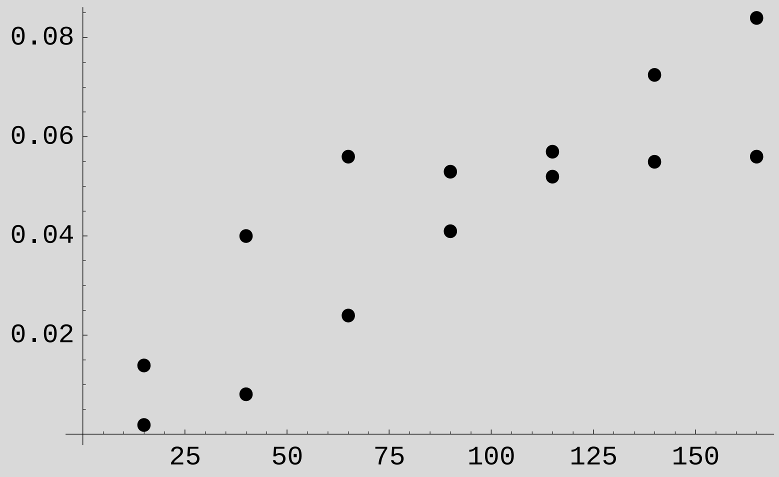

```
Show[P3, P1, P2, AxesLabel → {"Time(min)", nM},
  PlotLabel → "RNA Splicing", DisplayFunction → $DisplayFunction];
```

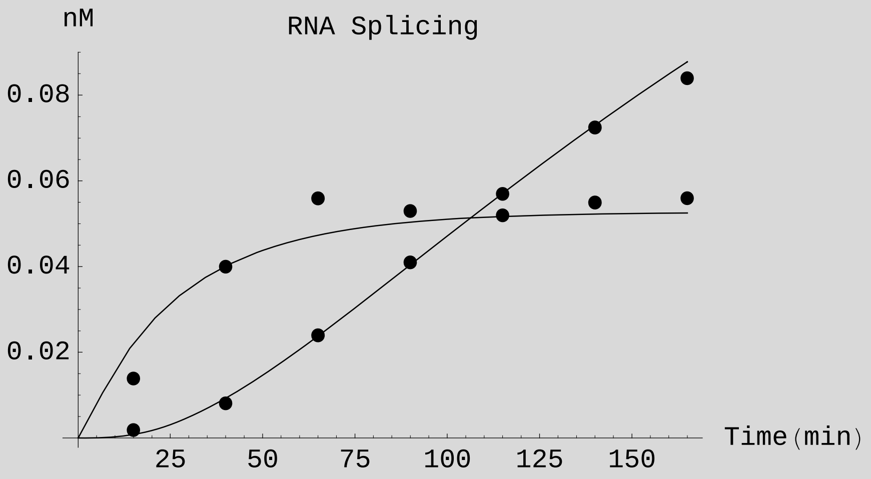

```
<< Graphics`MultipleListPlot`

(* mRNA concentration (nM) *)
mRNA = {
   {{15, 0.0019}, ErrorBar[0.001]},
   {{40, 0.0081}, ErrorBar[0.004]},
   {{65, 0.024}, ErrorBar[0.006]},
   {{90, 0.041}, ErrorBar[0.003]},
   {{115, 0.057}, ErrorBar[0.013]},
   {{140, 0.0725}, ErrorBar[0.012]},
   {{165, 0.084}, ErrorBar[0.0]}
  };

(* pre-mRNA concentration (nM) *)
premRNA = {
   {{15, 0.0139}, ErrorBar[0.0067]},
   {{40, 0.04}, ErrorBar[0.0085]},
   {{65, 0.056}, ErrorBar[0.02]},
   {{90, 0.053}, ErrorBar[0.022]},
   {{115, 0.052}, ErrorBar[0.015]},
   {{140, 0.055}, ErrorBar[0.012]},
   {{165, 0.056}, ErrorBar[0.0]}
  };

P5 = MultipleListPlot[premRNA, mRNA,
   AxesLabel → {"Time(min)", nM}, PlotLabel → "RNA Splicing",
   SymbolShape → {PlotSymbol[Diamond], PlotSymbol[Box]},
   SymbolStyle → {RGBColor[1, 0, 0], RGBColor[0, 1, 0]}
  ];
```

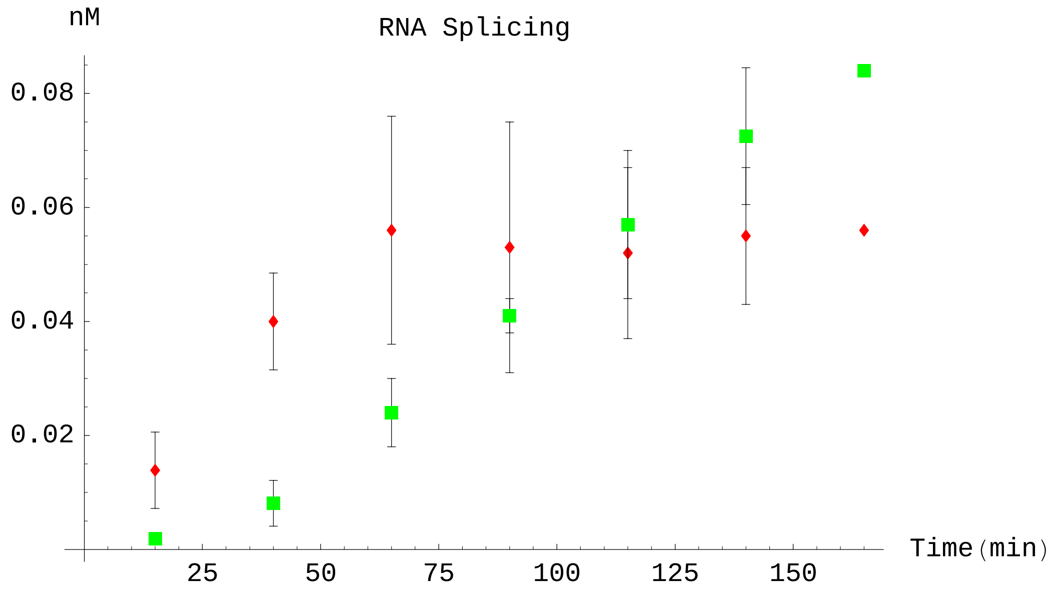
nM
RNA Splicing
0.08
0.06
0.04
0.02
25
50
75
100
125
150
Time(min)

```
Show[P5, P1, P2, AxesLabel → {"Time(min)", nM},
  PlotLabel → "RNA Splicing", DisplayFunction → $DisplayFunction];
```

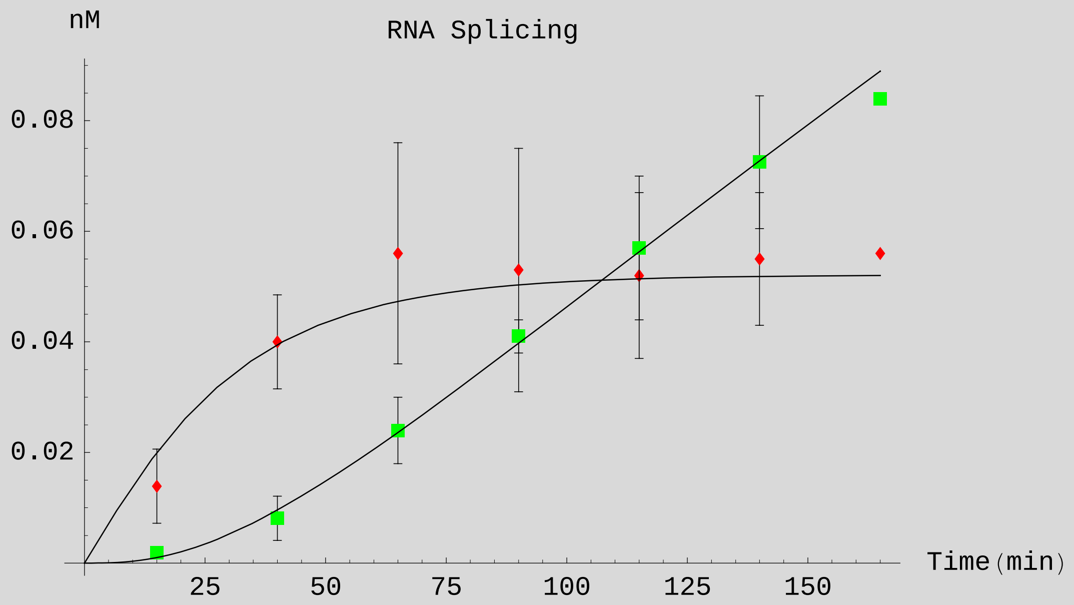

```
(* KmNEs$premRNA=1; KmNEd$premRNA=50 *)

P11 = Plot[mRNA[t] /. mySolution, {t, 0, tmax},
   PlotRange → All, PlotStyle → {RGBColor[0, 1, 0]}, DisplayFunction → Identity];
P21 = Plot[totalPremRNA[t] /. mySolution, {t, 0, tmax}, PlotRange → All,
   PlotStyle → {RGBColor[1, 0, 0]}, DisplayFunction → Identity];
Show[P11, P21, AxesLabel → {"Time(min)", nM}, PlotLabel → "RNA Splicing",
  DisplayFunction → $DisplayFunction];
```

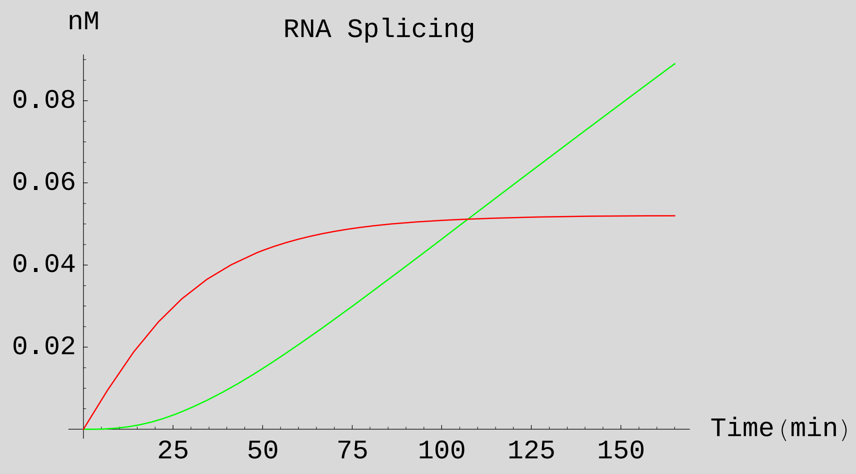

```
Show[P5, P11, P21, AxesLabel → {"Time(min)", nM},
  PlotLabel → "RNA Splicing", DisplayFunction → $DisplayFunction];
```

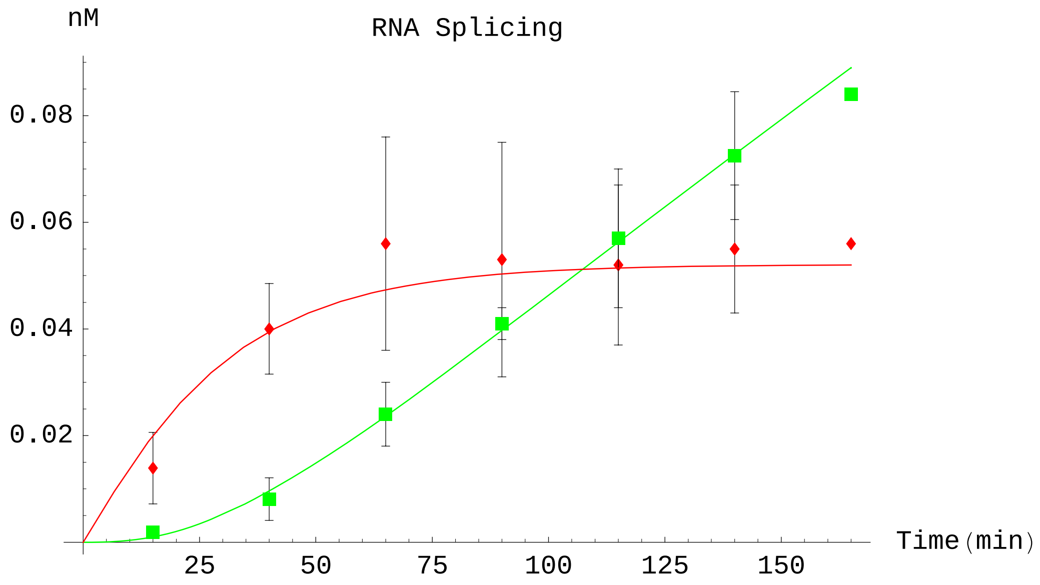

```
(* KmNEs$premRNA=10; KmNEd$premRNA=50 *)

P110 = Plot[mRNA[t] /. mySolution, {t, 0, tmax},
   PlotRange → All, PlotStyle → {RGBColor[0, 1, 0]}, DisplayFunction → Identity];
P210 = Plot[totalPremRNA[t] /. mySolution, {t, 0, tmax}, PlotRange → All,
   PlotStyle → {RGBColor[1, 0, 0]}, DisplayFunction → Identity];
Show[P110, P210, AxesLabel → {"Time(min)", nM}, PlotLabel → "RNA Splicing",
  DisplayFunction → $DisplayFunction];
```

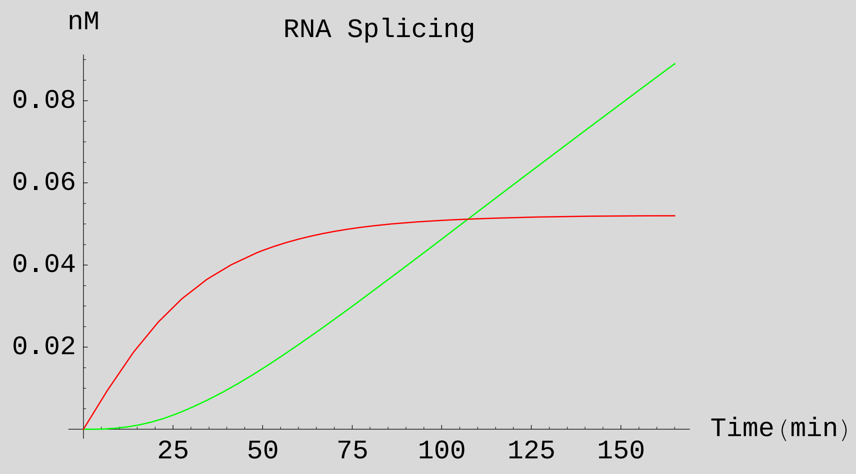

```
Show[P5, P110, P210, AxesLabel → {"Time(min)", nM},
  PlotLabel → "RNA Splicing", DisplayFunction → $DisplayFunction];
```

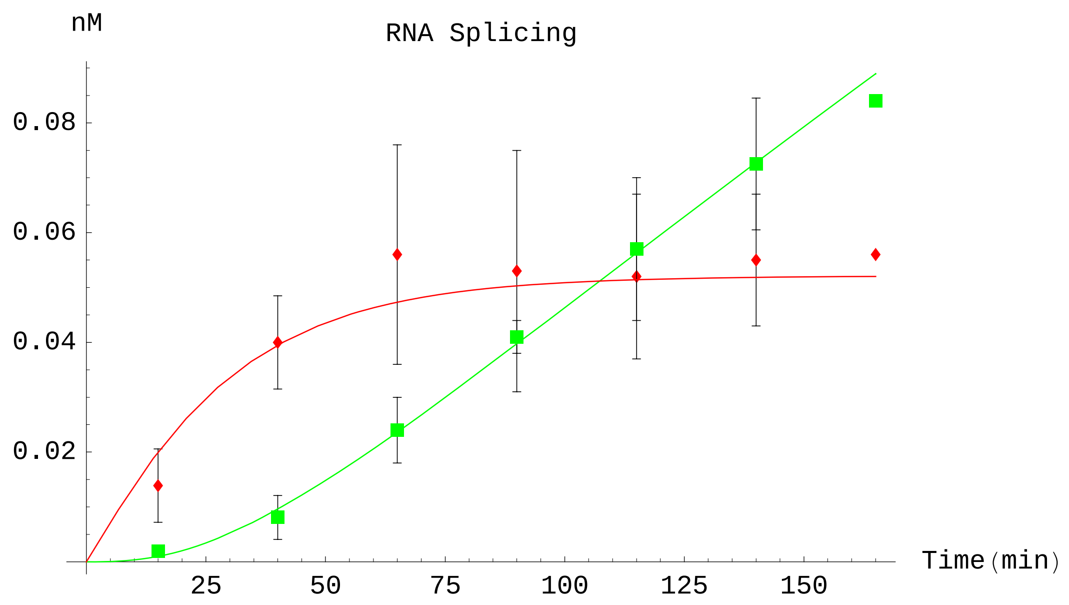

```
(* KmNEs$premRNA=50; KmNEd$premRNA=50 *)

P150 = Plot[mRNA[t] /. mySolution, {t, 0, tmax},
   PlotRange → All, PlotStyle → {RGBColor[0, 1, 0]}, DisplayFunction → Identity];
P250 = Plot[totalPremRNA[t] /. mySolution, {t, 0, tmax}, PlotRange → All,
   PlotStyle → {RGBColor[1, 0, 0]}, DisplayFunction → Identity];
Show[P150, P250, AxesLabel → {"Time(min)", nM}, PlotLabel → "RNA Splicing",
  DisplayFunction → $DisplayFunction];
```

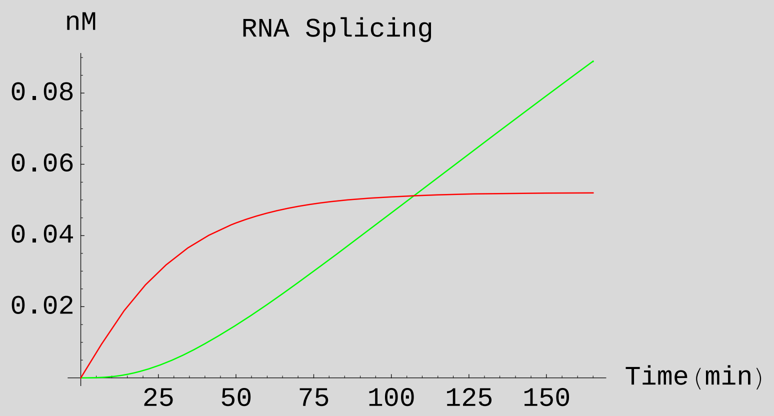

```
Show[P5, P150, P250, AxesLabel → {"Time(min)", nM},
  PlotLabel → "RNA Splicing", DisplayFunction → $DisplayFunction];
```

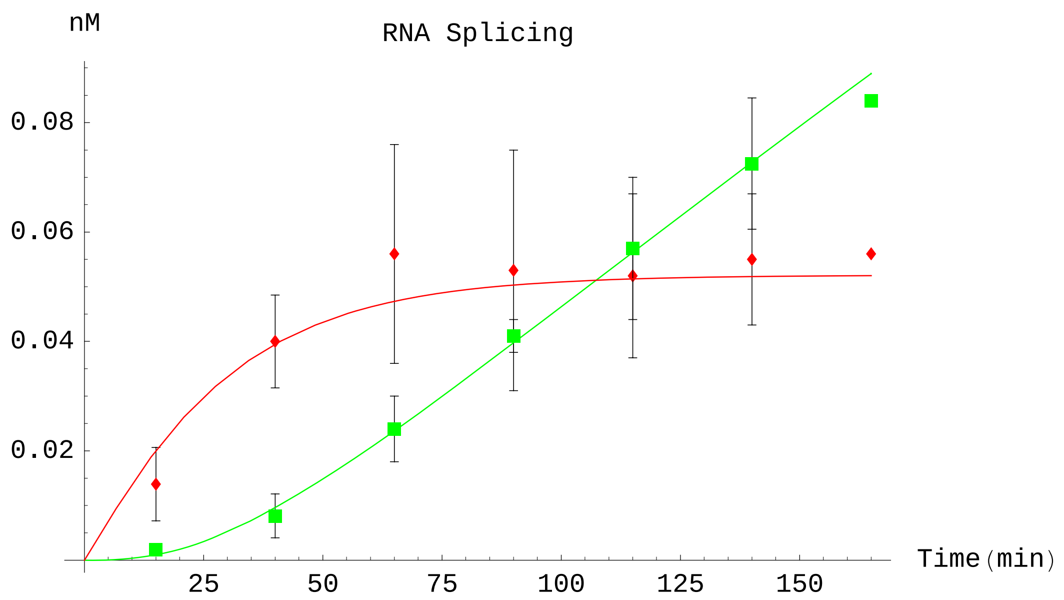

```
<< Statistics`ContinuousDistributions`
<< Graphics`MultipleListPlot`
<< myPadRight.m;
<< cellerator.m;
<< kMech_Rev.m;
Off[General::"spell1"];
Off[General::"spell"];
```

```
myPadRight Version 0.3 for Cellerator Loaded.
```

```
Cellerator™ Version 1.0 update 3.1002, loaded at Feb. 4, 2004, 00:45:44
©2001,2002 Jet Propulsion Laboratory, California Institute of
  Technology.  U.S. Government Sponsorship Acknowledged. All rights reserved.
Patent Pending (USPTO App 09993291).
The contents of this file may not be
  copied, distributed or transferred without written permission.
```

```
Cellerator™ 1.0 update 3.1002 load (using Mathematica Version 4.2
   for Microsoft Windows (June 5, 2002)) complete at February 4, 2004 00:45:46
```

```
 kMech is loaded
```

```
(* Start mRNA Splcing Simulation *)
(* NE1: RNA polymerase; NEs: spliceosome; NEd: degradation enzymes *)

mRNASplcing = Union[
  Enz[{DNA, NTP} ⇄(NE1) {premRNA, DNA}, BiBi[kf1, kr2, kcat3]],
  {{premRNA + NEs ⇄ $Complex$NEs$premRNA$, kf4, kr5}},
  {{$Complex$NEs$premRNA$ → NEs + mRNA, kcat6}},
  {{premRNA ⇄(NEd) NMP, kf7, kr8, kcat9}},
  {{$Complex$NEs$premRNA$ ⇄(NEd) NMP, kf10, kr11, kcat12}},
  {{mRNA ⇄(NEd) NMP, kf13, kr14, kcat15}}
 ]

{{$Complex$NE1$DNA$NTP$ → DNA + NE1 + premRNA, kcat3},
 {$Complex$NEs$premRNA$ → mRNA + NEs, kcat6}, {DNA + NE1 + NTP ⇄ $Complex$NE1$DNA$NTP$, kf1, kr2},
 {NEs + premRNA ⇄ $Complex$NEs$premRNA$, kf4, kr5}, {mRNA ⇄(NEd) NMP, kf13, kr14, kcat15},
 {premRNA ⇄(NEd) NMP, kf7, kr8, kcat9}, {$Complex$NEs$premRNA$ ⇄(NEd) NMP, kf10, kr11, kcat12}}
```

```
interpret[mRNASplcing]
```

```
{{DNA′[t] == -kf1 DNA[t] NE1[t] NTP[t] + kcat3 $Complex$NE1$DNA$NTP$[t] + kr2 $Complex$NE1$DNA$NTP$[t],
  mRNA′[t] == -kf13 mRNA[t] NEd[t] + kr14 $Complex$mRNA$NEd$[t] + kcat6 $Complex$NEs$premRNA$[t],
  NE1′[t] == -kf1 DNA[t] NE1[t] NTP[t] + kcat3 $Complex$NE1$DNA$NTP$[t] + kr2 $Complex$NE1$DNA$NTP$[t],
  NEd′[t] == -kf13 mRNA[t] NEd[t] - kf7 NEd[t] premRNA[t] + kcat15 $Complex$mRNA$NEd$[t] +
    kr14 $Complex$mRNA$NEd$[t] - kf10 NEd[t] $Complex$NEs$premRNA$[t] +
    kcat9 $Complex$premRNA$NEd$[t] + kr8 $Complex$premRNA$NEd$[t] +
    kcat12 $Complex$$Complex$NEs$premRNA$$NEd$[t] + kr11 $Complex$$Complex$NEs$premRNA$$NEd$[t],
  NEs′[t] == -kf4 NEs[t] premRNA[t] + kcat6 $Complex$NEs$premRNA$[t] + kr5 $Complex$NEs$premRNA$[t],
  NMP′[t] == kcat15 $Complex$mRNA$NEd$[t] +
    kcat9 $Complex$premRNA$NEd$[t] + kcat12 $Complex$$Complex$NEs$premRNA$$NEd$[t],
  NTP′[t] == -kf1 DNA[t] NE1[t] NTP[t] + kr2 $Complex$NE1$DNA$NTP$[t],
  premRNA′[t] == -kf7 NEd[t] premRNA[t] - kf4 NEs[t] premRNA[t] +
    kcat3 $Complex$NE1$DNA$NTP$[t] + kr5 $Complex$NEs$premRNA$[t] + kr8 $Complex$premRNA$NEd$[t],
  $Complex$mRNA$NEd$′[t] == kf13 mRNA[t] NEd[t] - kcat15 $Complex$mRNA$NEd$[t] -
    kr14 $Complex$mRNA$NEd$[t], $Complex$NE1$DNA$NTP$′[t] ==
   kf1 DNA[t] NE1[t] NTP[t] - kcat3 $Complex$NE1$DNA$NTP$[t] - kr2 $Complex$NE1$DNA$NTP$[t],
  $Complex$NEs$premRNA$′[t] == kf4 NEs[t] premRNA[t] - kcat6 $Complex$NEs$premRNA$[t] -
    kr5 $Complex$NEs$premRNA$[t] - kf10 NEd[t] $Complex$NEs$premRNA$[t] +
    kr11 $Complex$$Complex$NEs$premRNA$$NEd$[t], $Complex$premRNA$NEd$′[t] ==
   kf7 NEd[t] premRNA[t] - kcat9 $Complex$premRNA$NEd$[t] - kr8 $Complex$premRNA$NEd$[t],
  $Complex$$Complex$NEs$premRNA$$NEd$′[t] == kf10 NEd[t] $Complex$NEs$premRNA$[t] -
    kcat12 $Complex$$Complex$NEs$premRNA$$NEd$[t] - kr11 $Complex$$Complex$NEs$premRNA$$NEd$[t]},
 {DNA, mRNA, NE1, NEd, NEs, NMP, NTP, premRNA, $Complex$mRNA$NEd$, $Complex$NE1$DNA$NTP$,
  $Complex$NEs$premRNA$, $Complex$premRNA$NEd$, $Complex$$Complex$NEs$premRNA$$NEd$}}
```

```
graphList1 = {};
graphList2 = {};

For[iter = 1, iter < 100,
```

```
{myODEs, myVars} =
  {{DNA′[t] == -kf1 DNA[t] NE1[t] NTP[t] + kcat3 $Complex$NE1$DNA$NTP$[t] + kr2 $Complex$NE1$DNA$NTP$[t],
    mRNA′[t] == -kf13 mRNA[t] NEd[t] + kr14 $Complex$mRNA$NEd$[t] +
      kcat6 $Complex$NEs$premRNA$[t] + Random[NormalDistribution[0, 0.00001]],

    NE1′[t] == -kf1 DNA[t] NE1[t] NTP[t] + kcat3 $Complex$NE1$DNA$NTP$[t] + kr2 $Complex$NE1$DNA$NTP$[t],
    NEd′[t] == -kf13 mRNA[t] NEd[t] - kf7 NEd[t] premRNA[t] + kcat15 $Complex$mRNA$NEd$[t] +
      kr14 $Complex$mRNA$NEd$[t] - kf10 NEd[t] $Complex$NEs$premRNA$[t] +
      kcat9 $Complex$premRNA$NEd$[t] + kr8 $Complex$premRNA$NEd$[t] +
      kcat12 $Complex$$Complex$NEs$premRNA$$NEd$[t] + kr11 $Complex$$Complex$NEs$premRNA$$NEd$[t],
    NEs′[t] == -kf4 NEs[t] premRNA[t] + kcat6 $Complex$NEs$premRNA$[t] + kr5 $Complex$NEs$premRNA$[t],
    NMP′[t] == kcat15 $Complex$mRNA$NEd$[t] + kcat9 $Complex$premRNA$NEd$[t] +
      kcat12 $Complex$$Complex$NEs$premRNA$$NEd$[t],
    NTP′[t] == -kf1 DNA[t] NE1[t] NTP[t] + kr2 $Complex$NE1$DNA$NTP$[t],
    premRNA′[t] == -kf7 NEd[t] premRNA[t] - kf4 NEs[t] premRNA[t] +
      kcat3 $Complex$NE1$DNA$NTP$[t] + kr5 $Complex$NEs$premRNA$[t] +
      kr8 $Complex$premRNA$NEd$[t] + Random[NormalDistribution[0, 0.00001]],

    $Complex$mRNA$NEd$′[t] == kf13 mRNA[t] NEd[t] -
      kcat15 $Complex$mRNA$NEd$[t] - kr14 $Complex$mRNA$NEd$[t], $Complex$NE1$DNA$NTP$′[t] ==
     kf1 DNA[t] NE1[t] NTP[t] - kcat3 $Complex$NE1$DNA$NTP$[t] - kr2 $Complex$NE1$DNA$NTP$[t],
    $Complex$NEs$premRNA$′[t] == kf4 NEs[t] premRNA[t] - kcat6 $Complex$NEs$premRNA$[t] -
      kr5 $Complex$NEs$premRNA$[t] - kf10 NEd[t] $Complex$NEs$premRNA$[t] +
      kr11 $Complex$$Complex$NEs$premRNA$$NEd$[t], $Complex$premRNA$NEd$′[t] ==
     kf7 NEd[t] premRNA[t] - kcat9 $Complex$premRNA$NEd$[t] - kr8 $Complex$premRNA$NEd$[t],
    $Complex$$Complex$NEs$premRNA$$NEd$′[t] == kf10 NEd[t] $Complex$NEs$premRNA$[t] -
      kcat12 $Complex$$Complex$NEs$premRNA$$NEd$[t] - kr11 $Complex$$Complex$NEs$premRNA$$NEd$[t]},
   {DNA, mRNA, NE1, NEd, NEs, NMP, NTP, premRNA, $Complex$mRNA$NEd$, $Complex$NE1$DNA$NTP$,
    $Complex$NEs$premRNA$, $Complex$premRNA$NEd$, $Complex$$Complex$NEs$premRNA$$NEd$}};

Lamda = 1;
```

```
 myKs = {

   KmNE1$DNA = 2; KmNE1$NTP = 1000; kcat3 = Random[NormalDistribution[0.6, 0.05]];
   kf1 → Kf2S[KmNE1$DNA, KmNE1$NTP, kcat3, Lamda],
   kr2 → Kr[kcat3, Lamda],

   KmNEs$premRNA = 1; kcat6 = Random[NormalDistribution[140, 15]];
   kf4 → Kf[KmNEs$premRNA, kcat6, Lamda],
   kr5 → Kr[kcat6, Lamda],

    KmNEd$premRNA = 50; kcat9 = 10;
   kf7 → Kf[KmNEd$premRNA, kcat9, Lamda],
   kr8 → Kr[kcat9, Lamda],

   KmNEd$Complex$NEs$premRNA = 1000; kcat12 = 10;
   kf10 → Kf[KmNEd$Complex$NEs$premRNA, kcat12, Lamda],
   kr11 → Kr[kcat12, Lamda],

   KmNEd$mRNA = 50; kcat15 = 10;
   kf13 → Kf[KmNEd$mRNA, kcat15, Lamda],
   kr14 → Kr[kcat15, Lamda]
  };

myICs = {
   DNA[0] == 12,
   NTP[0] == 25000,
   premRNA[0] == 0,
   mRNA[0] == 0,
   NMP[0] == 0,

   NE1[0] == 0.001,
```

```
    NEs[0] == 0.0002,
    NEd[0] == 0.02,

    $Complex$NE1$DNA$NTP$[0] == 0,
    $Complex$NEs$premRNA$[0] == 0,
    $Complex$premRNA$NEd$[0] == 0,
    $Complex$$Complex$NEs$premRNA$$NEd$[0] == 0,
    $Complex$mRNA$NEd$[0] == 0
   };

tmax = 150;
 mySolution = NDSolve[Join[myODEs /. myKs, myICs], myVars, {t, 0, tmax}, MaxSteps → 3000];

 graphList1 = Join[graphList1,
   {Plot[mRNA[t] /. mySolution, {t, 0, tmax}, PlotRange → All, DisplayFunction → Identity]}];

 totalPremRNA[t_] = premRNA[t] + $Complex$NEs$premRNA$[t] + $Complex$premRNA$NEd$[t];
 graphList2 = Join[graphList2,
   {Plot[totalPremRNA[t] /. mySolution, {t, 0, tmax}, PlotRange → All, DisplayFunction → Identity]}];

 iter++];

(* mRNA concentration (nM) *)
mRNA = {
   {{15, 0.0013}, ErrorBar[0.0012]},
   {{40, 0.0051}, ErrorBar[0.0027]},
   {{65, 0.0155}, ErrorBar[0.0029]},
   {{90, 0.0262}, ErrorBar[0.0037]},
   {{115, 0.0360}, ErrorBar[0.0072]},
   {{140, 0.0461}, ErrorBar[0.0025]}
  };
```

```
(* pre-mRNA concentration (nM) *)
premRNA = {
   {{15, 0.0034}, ErrorBar[0.0026]},
   {{40, 0.0137}, ErrorBar[0.0025]},
   {{65, 0.0187}, ErrorBar[0.0047]},
   {{90, 0.0173}, ErrorBar[0.0050]},
   {{115, 0.0179}, ErrorBar[0.0040]},
   {{140, 0.0188}, ErrorBar[0.0012]}
  };

DataPlot = MultipleListPlot[premRNA, mRNA,
   SymbolShape → {PlotSymbol[Diamond], PlotSymbol[Box]},
   SymbolStyle → {RGBColor[1, 0, 0], RGBColor[0, 1, 0]}
  ];

Show[DataPlot, graphList1, graphList2, AxesLabel → {"Time(min)", nM},
  PlotLabel → "RNA Splicing", DisplayFunction → $DisplayFunction];

Clear[graphList1, graphList2]
```

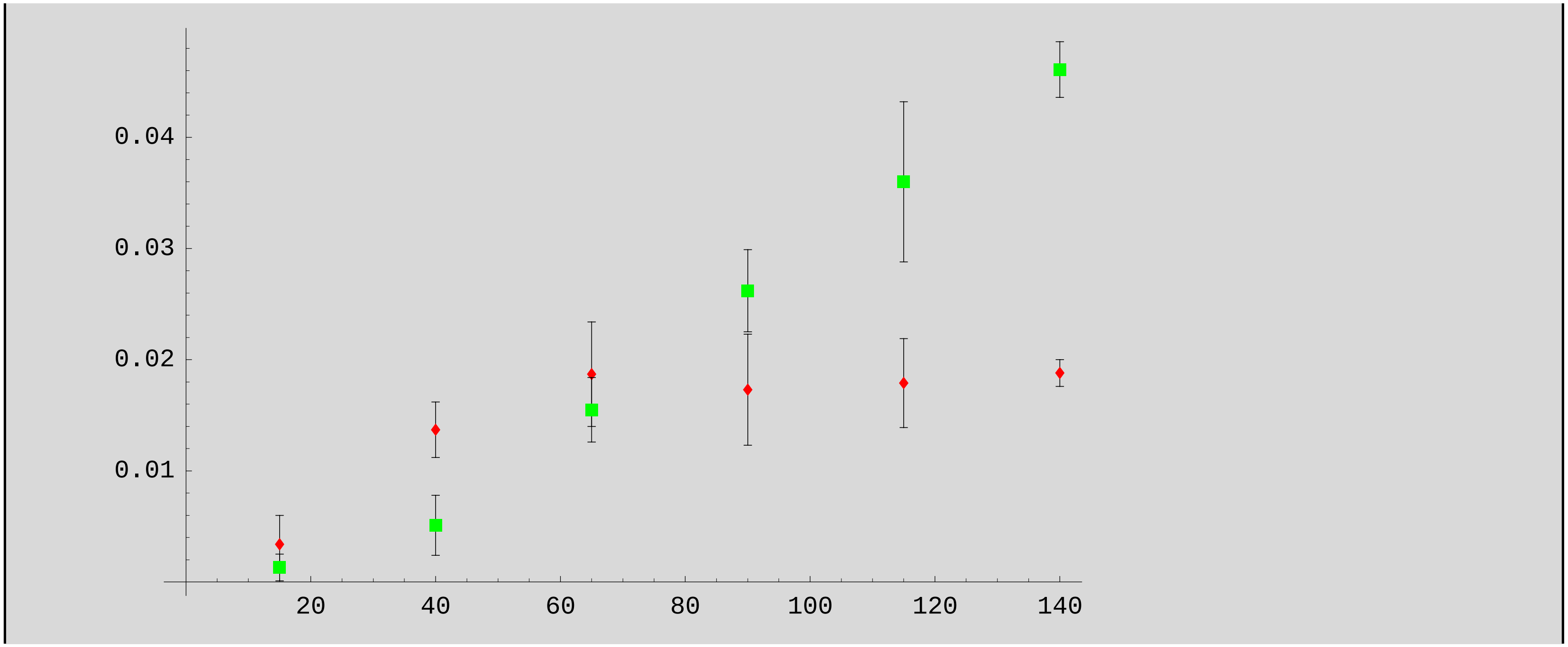
0.04
0.03
0.02
0.01
20
40
60
80
100
120
140

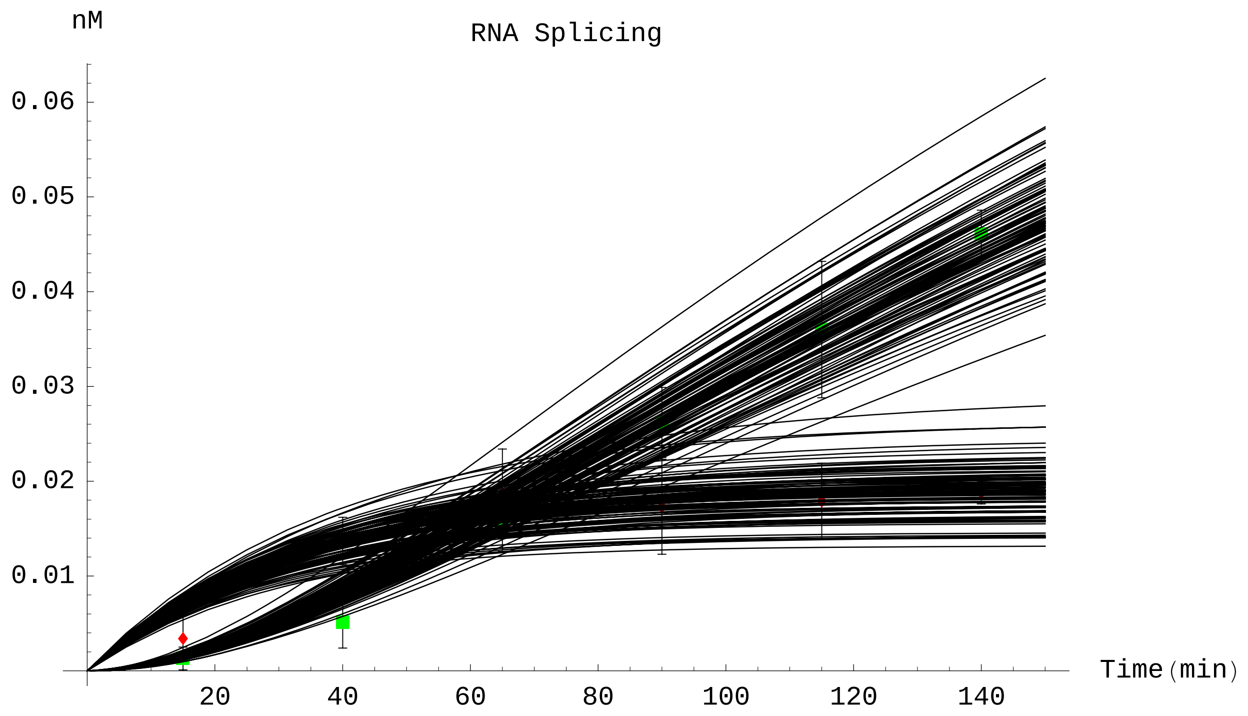
nM
RNA Splicing
0.06
0.05
0.04
0.03
0.02
0.01
20
40
60
80
100
120
140
Time(min)

```
myODEs

{DNA′[t] == -kf1 DNA[t] NE1[t] NTP[t] + 0.386444 $Complex$NE1$DNA$NTP$[t] + kr2 $Complex$NE1$DNA$NTP$[t],
 mRNA′[t] ==
  0.000012116 - kf13 mRNA[t] NEd[t] + kr14 $Complex$mRNA$NEd$[t] + 105.435 $Complex$NEs$premRNA$[t],
 NE1′[t] == -kf1 DNA[t] NE1[t] NTP[t] + 0.386444 $Complex$NE1$DNA$NTP$[t] + kr2 $Complex$NE1$DNA$NTP$[t],
 NEd′[t] == -kf13 mRNA[t] NEd[t] - kf7 NEd[t] premRNA[t] + 10 $Complex$mRNA$NEd$[t] +
    kr14 $Complex$mRNA$NEd$[t] - kf10 NEd[t] $Complex$NEs$premRNA$[t] +
    10 $Complex$premRNA$NEd$[t] + kr8 $Complex$premRNA$NEd$[t] +
    10 $Complex$$Complex$NEs$premRNA$$NEd$[t] + kr11 $Complex$$Complex$NEs$premRNA$$NEd$[t],
 NEs′[t] == -kf4 NEs[t] premRNA[t] + 105.435 $Complex$NEs$premRNA$[t] + kr5 $Complex$NEs$premRNA$[t],
 NMP′[t] ==
  10 $Complex$mRNA$NEd$[t] + 10 $Complex$premRNA$NEd$[t] + 10 $Complex$$Complex$NEs$premRNA$$NEd$[t],
 NTP′[t] == -kf1 DNA[t] NE1[t] NTP[t] + kr2 $Complex$NE1$DNA$NTP$[t],
 premRNA′[t] == 8.74921 × 10^-7 - kf7 NEd[t] premRNA[t] - kf4 NEs[t] premRNA[t] +
    0.386444 $Complex$NE1$DNA$NTP$[t] + kr5 $Complex$NEs$premRNA$[t] + kr8 $Complex$premRNA$NEd$[t],
 $Complex$mRNA$NEd$′[t] == kf13 mRNA[t] NEd[t] - 10 $Complex$mRNA$NEd$[t] - kr14 $Complex$mRNA$NEd$[t],
 $Complex$NE1$DNA$NTP$′[t] ==
  kf1 DNA[t] NE1[t] NTP[t] - 0.386444 $Complex$NE1$DNA$NTP$[t] - kr2 $Complex$NE1$DNA$NTP$[t],
 $Complex$NEs$premRNA$′[t] == kf4 NEs[t] premRNA[t] - 105.435 $Complex$NEs$premRNA$[t] -
    kr5 $Complex$NEs$premRNA$[t] - kf10 NEd[t] $Complex$NEs$premRNA$[t] +
    kr11 $Complex$$Complex$NEs$premRNA$$NEd$[t], $Complex$premRNA$NEd$′[t] ==
  kf7 NEd[t] premRNA[t] - 10 $Complex$premRNA$NEd$[t] - kr8 $Complex$premRNA$NEd$[t],
 $Complex$$Complex$NEs$premRNA$$NEd$′[t] == kf10 NEd[t] $Complex$NEs$premRNA$[t] -
    10 $Complex$$Complex$NEs$premRNA$$NEd$[t] - kr11 $Complex$$Complex$NEs$premRNA$$NEd$[t]}
```
